# Citizen preferences for online hate speech regulation

**DOI:** 10.1093/pnasnexus/pgaf032

**Published:** 2025-02-12

**Authors:** Simon Munzert, Richard Traunmüller, Pablo Barberá, Andrew Guess, JungHwan Yang

**Affiliations:** Data Science Lab, Hertie School, 10017 Berlin, BE, Germany; School of Social Sciences, University of Mannheim, 68159 Mannheim, BW, Germany; Department of Political Science and International Relations, University of Southern California, Los Angeles, CA 90089, USA; Department of Politics and School of Public and International Affairs, Princeton University, Princeton, NJ 08544, USA; Department of Communication, University of Illinois at Urbana-Champaign, Champaign, IL 61820, USA

**Keywords:** hate speech, experiment, preferences, moderation, regulation

## Abstract

The shift of public discourse to online platforms has intensified the debate over content moderation by platforms and the regulation of online speech. Designing rules that are met with wide acceptance requires learning about public preferences. We present a visual vignette study using a sample (n=2,622) of German and US citizens that were exposed to 20,976 synthetic social media vignettes mimicking actual cases of hateful speech. We find people’s evaluations to be primarily shaped by message type and severity, and less by contextual factors. While focused measures like deleting hateful content are popular, more extreme sanctions like job loss find little support even in cases of extreme hate. Further evidence suggests in-group favoritism among political partisans. Experimental evidence shows that exposure to hateful speech reduces tolerance of unpopular opinions.

**Significance Statement**

In a world that is increasingly digitally connected, hate speech has become a central concern across the globe. Yet, whether and how to restrict speech considered offensive or promoting hate toward particular groups is highly contentious. Using thousands of synthetic social media posts and putting participants into the role of a content moderator, this study breaks ground by providing rich evidence on public preferences for what should or should not be allowed online. The results confirm that even in two contexts with different traditions of free expression, there is a consensus to sanction extreme forms of hateful speech but not speakers based on their identity. At the same time, people’s evaluations are biased toward their own ideological camp.

## Introduction

The shift of public discourse to online platforms and the resulting visibility of hateful content have intensified the debate over the regulation of online speech (1–3). Since social media companies practice content moderation opaquely and largely without independent oversight (4, 5), policymakers worldwide have started to take action on online hate speech (6, 7). But these efforts are largely uninformed by public preferences. While survey evidence suggests strong cross-national variation in support for freedom of expression (8, 9), citizens’ preferences for online hate speech regulation remain poorly understood.

Our research aims to study what citizens think about the limits of public speech online, as well as how they balance the goals of freedom, group equality, and the prevention of harm. In particular, we are guided by the following overarching questions: (i) What do citizens deem acceptable or unacceptable speech in online public discourse?, (ii) How do citizens want online speech to be handled and regulated?, and (iii) Which factors determine these preferences, and how do cultural and policy context matter?

**Fig. 1.**
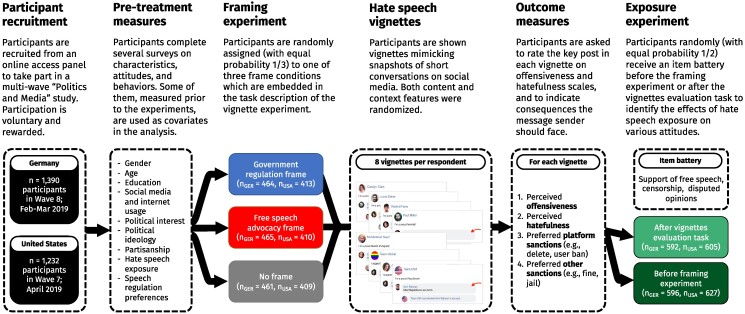
Overview of study design. Subjects in both a German (n=1,390) and a US (n=1,232) sample were randomly assigned to one of three framing conditions and then sequentially shown 8 vignettes with evaluation prompts according to various criteria. Downstream consequences of hate post exposure on issue attitudes were measured with between-subjects randomization of the vignette task placement.

To answer these questions, we implemented (i) a vignette study combined with (ii) a framing and (iii) an exposure experiment with a sample (n=2,622) of German and US citizens (Fig. 1; see Materials and methods for details). Studying these two countries allows us to explore preferences for hate speech regulation under two ideal-typical conditions. The United States and Germany not only differ in their cultural tradition concerning the norms of free expression but also in their actual hate speech legislation (10, 11). Although much of the platform law is shaped by US-based companies, there are no hate speech laws in the United States and its tradition of the First Amendment is understood to offer broader protection to all forms of public expression. Germany, in contrast, legally sanctions acts of speech such as incitement to hatred, insult, or defamation and has been at the forefront of the European approach to mandating that social media companies tackle harmful content—including hate speech—online (12).

Next to struggles over definitions and the adequate regulatory response to hate speech, a key challenge in the study of citizens’ preferences is the fundamental context dependence of these questions (13). Classic survey instruments like “Would you support a law that prohibits hateful statements about [group]” focus on target group characteristics as a core defining feature of hate speech but ignore who speaks what and in which context—information that is critical for assessing the legitimacy of speech (14, 15). We offer a way to study the role of many of these content- and context-related features at once. Our citizens-as-moderators experiment exposed participants to 20,976 fictitious social media posts, visually and content-wise mimicking cases of hateful speech to bolster external validity (16, 17).

Our main findings corroborate and extend existing evidence: First, even without training, respondents make nuanced judgments, relying on heuristics consistent with theoretical understandings of speech types and their severity. As the severity of the attack increases, so does the level of support for sanctions (16, 17). Varying speech across several topics, we can furthermore show that hateful speech targeting marginalized and vulnerable groups, such as women and Muslim immigrants, is perceived as more problematic than speech targeting political partisans. Identities of senders and targets of speech, on the other hand, seem to matter less (18). At the same time, capitalizing on a broad range of possible sanctions we are able to show that there is no broad support for severe sanctions (such as permanent bans, job loss, or prison sentences) even in cases of extreme hate speech. Citizens in both countries seem to prefer either no sanctions at all or action targeted at the message (postdeletion) rather than the sender. Furthermore, our experimental setup allows us to shed new light on exposure effects of hate speech: we find that short-term exposure to hateful speech in an arbitration scenario reduces tolerance for extreme viewpoints and decreases support for an unrestricted, censorship-free internet, while at the same time exerting a desensitizing effect (19, 20).

Learning about public preferences is necessary in order to design regulations that are met with wide acceptance from general publics. We believe that the findings we present in this paper provide a fruitful starting point for an evidence-based approach to online content regulation in general.

## Hypotheses

What determines citizens’ evaluations of hateful speech and their preference for its regulation online? We divide our preregistered hypotheses into three separate blocks for analysis, each linked to an experimental component in the study setup. Experiment 1 focuses on content- and participant-level factors determining speech perceptions and action preferences. Experiment 2 evaluates a framing treatment to explore the role of societal context for preference formation. Experiment 3 evaluates the downstream effects of hate speech exposure on attitudes related to hate speech regulation.

### Experiment 1

**Fig. 2.**
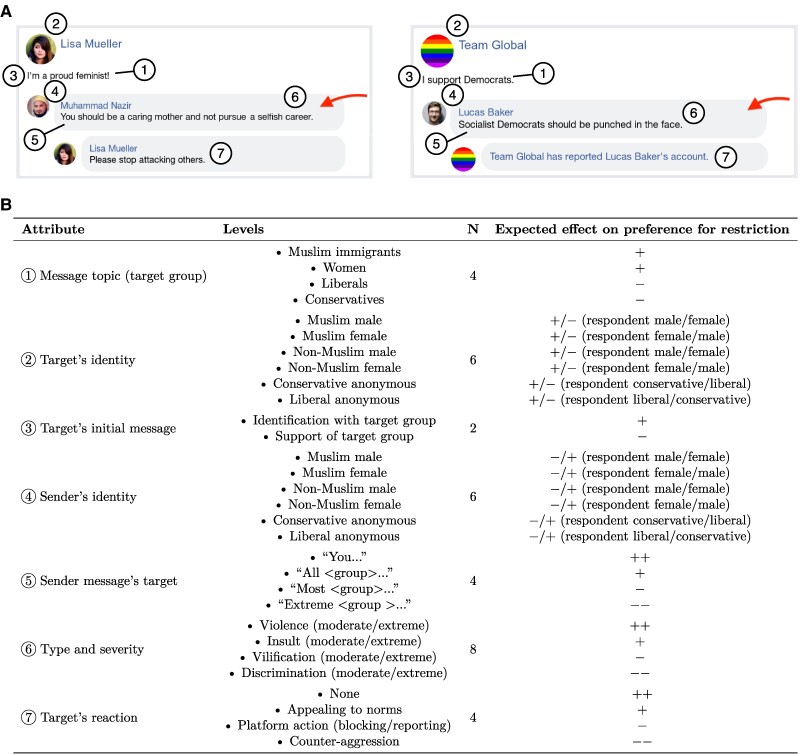
Vignette attributes, levels, and preregistered expectations. In A), the left example vignette showcases extreme discrimination against women by a male Muslim targeted at a female non-Muslim, with the target reacting by appealing to norms. The right example vignette showcases moderate violence against Liberals by a male non-Muslim targeted at a liberal anonymous account, with the target reacting by blocking the sender. B) Vignette attributes, levels, preregistered expectations. See Tables S1 to S4 for a full overview of attribute values by country and topic.

In a first set of expectations, we hypothesize that perceptions of speech as well as preferences toward its sanctioning are influenced by (i) content- and context-specific factors of the message itself and (ii) respondent-level characteristics. The expectations are summarized in Fig. 2 and correspond to specific vignette attributes.

#### Content and context

Existing hate speech regulations differ in terms of how speech restrictions are justified, i.e. by the particular manner of speech or by the likelihood of harmful consequences (3, 21, 22). In some countries, speech is prohibited that offends, insults or degrades a social group. In others, speech is prohibited if it is likely to have harmful effects such as violence or public disorder. Differences in type and severity are also considered in recent classification schemes of online hate speech (23, 24). To learn about citizens’ preferences for hate speech regulation, we therefore distinguish between different *types and degrees of severity* of speech. Since variations in severity of incivility and hate speech have been shown to matter for people’s perceptions (25–27), we expect stronger reactions and greater support for restrictions when respondents are confronted with more extreme versions (moderate vs. extreme) and types (discrimination, vilification, insult, and violence) of speech.

A core defining feature of hate speech (1, 3) is the targeting of disadvantaged or historically marginalized groups (e.g. as reflected in laws which are designed to protect racial or religious minorities.) Yet, there remains considerable disagreement over the specific groups that should be included in these categories. In our experimental setup, we therefore consider the role of *target groups* addressed in the message. Given the existence of strong social norms, we expect that content is perceived as more problematic and demanding of regulation when it targets vulnerable groups or groups defined by immutable characteristics, such as immigrants, religious minorities, or women. Conversely, we expect less evaluations as hateful or offensive, and fewer calls for regulation, when the message content involves the targeting of larger groups with mutable characteristics, such as partisan camps, where no such norms exist (28). Consistent with our reasoning about group identities, we expect respondents to react more strongly when the *target’s original message* expresses an explicit identification with (“I’m a…) rather than mere support of (“I support”) the target group. Last, we also consider the *addressing scope*, i.e. whether speech directly addresses the target personally (“You…”) or whether it makes generalizations about entire groups (“All <group>…,” “Most <group>…”). We expect that respondents will be more sensitive toward the former than the latter.

*Counterspeech* can be a promising strategy to curb hate speech by directly engaging with the perpetrator (29–31). However, it may make top-down interventions seem less urgent. We consider counterspeech by the target and expect respondents’ willingness to restrict speech to be reduced when it is present (although that could vary across different forms of counterspeech, which are tested in our setup).

#### Respondent characteristics

Both the perception of harmful speech and preferences for online regulation may differ across individuals. Thus, we study characteristics that have been shown to structure attitudes toward free speech and its regulation in general. These factors include gender (32, 33), age (34), education, political interest (35), social media usage (36), political ideology (37–39), experience with hate speech (19), and perceptions of free speech norms.

Finally, respondent characteristics can interact with target and sender attributes (40). Consistent with evidence of in-group favoritism in citizens’ judgments of attitudes and speech (41, 42), we hypothesize that citizens will be biased in favor of their own group identity. In the context of our study, this implies that if the respondent shares the gender or ideological identity of the target, they will be more prone to judge the controversial content as hateful and to penalize it harder. We expect effects in the opposite direction if the respondent shares identity characteristics with the sender of the message.

### Experiment 2

Second, we hypothesize that respondents’ perceptions of speech and their preferred regulatory response are shaped by normative principles that frame the problem and implications of hate speech regulation in specific ways. Two such conflicting normative principles are at the heart of the hate speech debate. Whereas the first principle is rooted in the liberal tradition that emphasizes the importance of protecting free speech against the intrusion of state authority, the second principle argues that free speech must be balanced against competing values and that speech should be restricted if it harms equal dignity of others (43). While the first principle is the dominant way of framing the free speech problem in the United States, the second characterizes the German approach to hate speech regulation (10). It has been shown that principles which manifest in government and civil society action can have measurable feedback effects on societal norms, e.g. in the cases of same-sex marriage policies (44) and smoking bans (45).

To assess the effect of these competing normative principles, we embed the vignette task in a framing experiment (46). We derive two major expectations from our setup: If the task is motivated by looming government regulation to censor offensive or hateful social media content protecting potential victims of hate speech, respondents, sensitized toward the interests of potential victims, will perceive the content shown as more offensive and hateful and be more prone to support tougher actions. If the task is motivated by civil liberties groups advocating for the right of free speech and against censorship online, respondents, sensitized toward the adverse effects of potential censorship, will perceive the content shown as less offensive and hateful and thus be less prone to support tougher actions. See Materials and methods for more details about the setup of the framing experiment.

### Experiment 3

Finally, we consider downstream consequences of exposure to hateful content as part of the evaluation task. A key concern with exposure to hate speech is that it induces psychological harm and stress among victims (47, 48) and encourages offline hate crimes by perpetrators (49–51). In addition, exposure to hate speech may lead to desensitization which changes the perception of hateful content and shapes preferences for its regulation (19).

In contrast to endogenous exposure to hate speech in the wild, we exploit our controlled setup and study the (short-term) effects of exogenous exposure to hateful content. An important limitation of this induced type of exposure is that it happens in the context of a moderation-style task where participants take the role of a judge, not target. This might affect their perceptions of as well as their willingness to tolerate such speech. We preregistered the following set of hypotheses: We expect that exposure to hateful speech reduces tolerance of such speech, that is, people expressing unpopular opinions that are deeply offensive to others. Second, we hypothesize that hate speech exposure also increases support for government censorship of content online. Third, we expect that exposure reduces people’s propensity to openly support derogatory statements about specific groups. The rationale behind our expectations is that the exposure, in particular in the context of the moderation-style task, boosts the salience of hateful speech as a problem, making people less tolerant of it and becoming aware of the socially undesirable nature of such statements.

## Design

###  

#### Data collection and samples

To test our hypotheses, we combine a framing and a vignette experiment with a hate speech exposure experiment (Fig. 1). They were embedded in two multiwave panel surveys fielded on initially n=1,551 respondents recruited for the YouGov US Pulse panel and n=1,516 respondents recruited for the YouGov German Pulse panel, which were also used for research unrelated to this study (52, 53). The waves in which the experiments were embedded were fielded from 2019 February 19 to 2019 March 13 in Germany (wave 8 of the study; n=1,390) and from 2019 April 1 to April 9 in the United States (wave 7 of the study; n=1,232).

#### Experimental setup

After surveying them about characteristics, attitudes, and behaviors in prior waves and the experimental wave, as part of *experiment 2* respondents were randomly assigned to one of three framing conditions—a government regulation frame, a free speech advocacy frame, and a neutral frame (54)—which were used to introduce the evaluation task. After the frame, respondents were shown eight vignettes in combination with several outcome measures (*experiment 1*). Furthermore, they were assigned to one of two conditions in the exposure experiment, which randomized the item order (*experiment 3*). Under the control condition, the respondents were shown a battery of items asking them about their support of free speech rights, internet censorship, and some extreme opinions before the framing experiment. Under the treatment condition, the respondents were shown the same item battery after the vignettes task.

To study individual perceptions and preferences, we use vignettes that are constructed in a way that mimics posts on a popular social media platform. At the core of the vignettes is a message posted by a sender of varied identity in response to a statement posted by the target. The messages were designed by the researchers so as to represent both moderate and extreme forms of discriminating, vilifying, insulting, and threatening speech. For instance, for speech against Muslim immigrants, the statements representing moderate and extreme discrimination were “Muslims should be banned from coming into our country” and “Muslims should be deported.” Importantly, “moderate” and “extreme” bears no absolute meaning here but is used to distinguish statements according to the graveness of their policy implications or extremity, providing more variation in the types of speech that are studied. Insulting (“[Target group members] are dumb” and “[Target group members] are stupid animals”) and threatening messages (“[Target group members] should be punched in the face” and “[Target group members] should be killed”) are kept constant across topics whereas discriminating and vilifying statements are adapted to the respective target group (see Tables S1 to S8 for an overview of all message modules by topic and sample). Post features such as time stamp and interaction buttons are not displayed, but we keep (and vary) features that represent relevant cues for the assessment task and that correspond to our content- and message context-related hypotheses. These attributes include substantive issues, sender as well as target characteristics, and sender message and target message characteristics. Figure 2 provides an overview of the attributes and attribute levels together with two vignette examples, as well as a summary of preregistered expectations for the vignette evaluation task. We then use hierarchical linear modeling with person, vignette deck, and country random effects (in pooled analyses) to estimate *average marginal component effects* (AMCE), which identify (55) the causal effect of randomly varied attributes on respondents’ reported perceptions and preferences. See Materials and methods for further details about the construction of vignettes and vignette decks, and the statistical analysis.

The combination of a vignette design with authentic features of social media conversations and actual instances of hateful speech provides us with a powerful instrument to measure citizens’ perceptions of hateful speech. In contrast to real-life hate messages, we have full control over key features, such as target and sender characteristics as well as subject, type, and severity of the message. This allows us to disentangle the influence of different components of posts which are confounded in real-world exposure. At the same time, the naturalistic setting has higher ecological validity than abstract statements about speech acts, which are commonly used to measure preferences about free speech regulation.

#### Outcome measures

The outcome measures can be grouped into three categories. First, we elicit perceptions of the severity of speech using two five-point offensiveness and hatefulness scales. These measurements allow us, first, to validate that the underlying posts are perceived by participants as cases of hateful speech and, second, to explore what factors structure perceptions of severity. Second, we elicit preferences for nuanced forms of platform action. These include sanctions against the speech act itself (deletion) as well as sanctions against the sender (preventing additional posts to the target, a temporary ban, and a permanent platform ban). Third, we measure preferences for further sanctions, including dismissal by the sender’s employer, a fine, and a prison sentence. Noticeably, in Germany but not in the United States there are statutory bases (incitement of the people, insult, defamation; §130, 185, and 187 of the German Criminal Code) that provide for both fines and imprisonment for hate speech and other speech-based offenses, which are also applied in practice. With this range of options, we can measure not only the perceived legitimacy of speech, but preferences that have direct implications for platform policies and regulatory action.

## Results

**Fig. 3.**
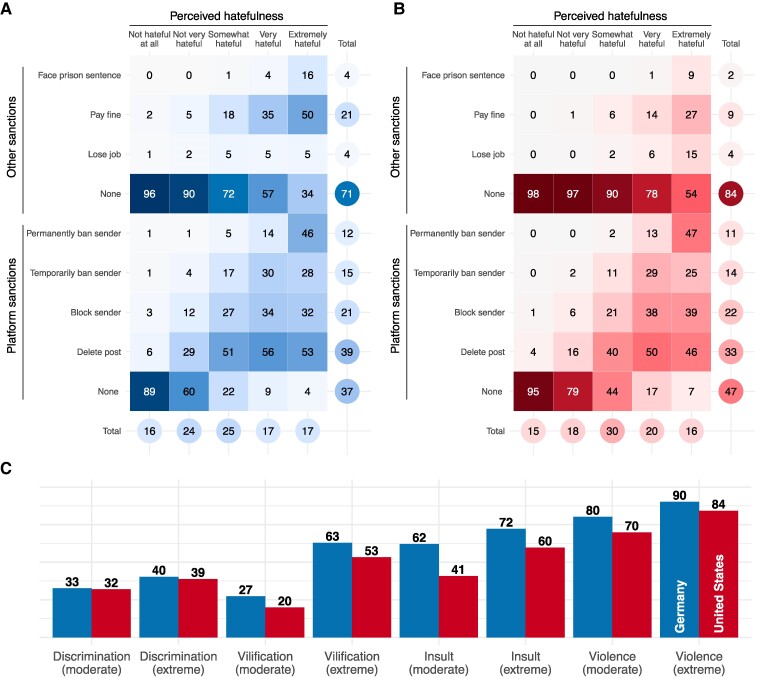
Perceptions and sanction preferences across all n=20,976 hate speech vignettes, by country (Germany: 1,390 respondents, United States: 1,232 respondents). All values represent rounded percentages. Values in squared cells represent column percentages, i.e. the proportion a certain sanction has been selected conditional on the level of perceived hatefulness; multiple sanctions could be selected per post. A) Preferred sanctions by hatefulness, Germany, B) Preferred sanctions by hatefulness, United States, C) Choice to sanction sender by type and severity of speech, by country.

We first characterize the key outcome measures of experiments 1 and 2, the evaluation scales for the speech vignettes (Fig. 3). A majority of the messages (over 60%) are perceived to be at least “somewhat hateful” by the respondents in both countries. There is substantial variation within the ratings, indicating that the posts, which were designed to be on the spectrum between potentially controversial to strongly offensive or hateful, are indeed exhausting the full evaluation scales. There are no major differences between samples. Perceived offensiveness and hatefulness ratings are strongly correlated (Pearson correlation of r=0.79 in the German sample and r=0.89 in the US sample; see also Fig. S12). The share of perceptions of messages as being very or extremely hateful range between 2/5% (“Women are just different from men”; US/GER sample) and 83/87% (“Women should be killed” US/GER sample) (see Figs. S9 and S10).

We find considerable variation in the sanctions that the respondents selected for the posts (Fig. 3). For 47% of the posts in the US sample (German sample: 37%), the respondents saw no need for action by the platform provider. On the other hand, for 11% of the posts (German sample: 12%), the respondents would have liked to see the sender of the message banned permanently from the platform. The other options are somewhere in between, while the order mirrors a plausible ladder of escalation. With regard to offline consequences for the sender, about 84% (German sample: 71%) of all social media posts were deemed as not requiring any further action. However, in about 4% of the cases respondents decided that the sender should lose their job, and for 9% (German sample: 21%) they chose a fine. A prison sentence was rarely considered an appropriate sanction (US sample: 2%; German sample: 4%). While the rankings of different actions are similar in Germany and the United States, country-level differences could indicate that cultural context or legal tradition matters for perceptions and preferences toward hateful content. For instance, fines for hate speech are not uncommon in Germany, and while custodial sentences are rare, they do happen (often on probation). The choice to impose any sanction (regardless of which platform or other sanctions were selected) on the sender is both related to the type and severity of speech and the country sample (Fig. 3C). For a majority of posts that were classified as cases of moderate or extreme discrimination and moderate vilification, respondents chose not to sanction them. On the other hand, 84% (German sample: 90%) of the posts classified as cases of extreme violence were sanctioned. These findings indicate that respondents clearly distinguish between different types and degrees of hateful speech, and that behavior that takes a strong position in defense of free speech (i.e. not sanction speech in the face of extreme violence), is fairly rare. Notably, respondents in the US sample were substantially more hesitant to sanction posts.

### Experiment 1: determinants of speech evaluations

Turning to the evidence of Experiment 1, we find that some content characteristics of posts matter a lot to respondents’ judgments, whereas context characteristics do not matter much.

**Fig. 4.**
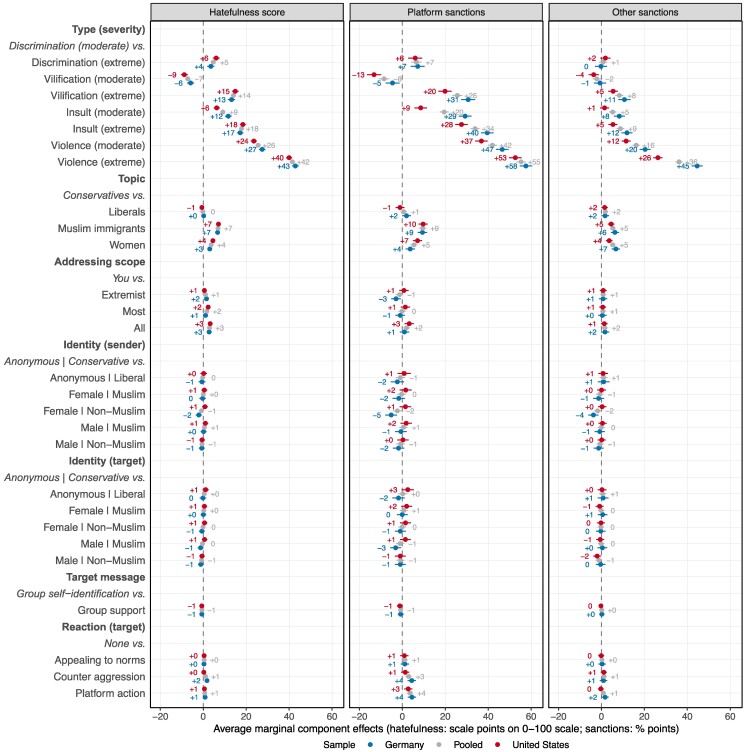
Estimated average marginal component effects of content and context characteristics of social media vignettes on citizens’ perceptions and preferred platform-side and other action, by country. Estimates from hierarchical linear models with person, vignette deck, and country random effects. Perceived hatefulness is rescaled to a 0–100 scale (originally 5-point scale). The choice of platform sanctions is encoded as a binary decision that takes the value 1 if the respondent chose at least one of the following options: delete post, block sender, temporarily ban sender, permanently ban sender. The choice of other sanctions is encoded as a binary decision that takes the value 1 if the respondent chose at least one of the following options: lose job, pay fine, face prison sentence. Error bars represent 95% CI. See Table S15 for detailed regression results.

In our analysis of AMCEs of content and context features on perceptions and preferred platform actions, we find very similar patterns across most evaluation criteria, which is why we focus on the hatefulness score and pool platform and other sanctions, respectively (Fig. 4). Also, we focus on the results for the pooled sample in the text.

First, in line with our expectations, we find that the type and severity of speech matter a lot for perceptions and preferences: perceived hatefulness differs by 42scale points (b=0.42; 95% CI=0.40–0.43; P<0.001; d.f.=18,411; $2\log_{e}\;[\text{BF}]=5,358$) on a 0–100 scale between posts of extreme violence compared to posts of moderate discrimination. Comparing AMCE sizes across type and severity attributes, extreme insults are 34 percentage points more likely (b=0.34; 95% CI = 0.32–0.36; P<0.001; d.f.=18,857; $2\log_{e}\;[\text{BF}]=1,018$), and extremely violent posts are 55 percentage points (b=0.55; 95% CI = 0.53–0.57; P<0.001; d.f.=18,869; $2\log_{e}\;[\text{BF}]=2,569$) more likely to elicit support for platform sanctions than moderately discriminatory posts. Substantively speaking, while the effects are massive, it is important to note that they do not imply consensus on the hatefulness or necessity of sanctions given certain levels of severity. To illustrate, “Women should be punched in the face” ranks among the six most hateful statements in both samples, however, only about 60% of participants perceive it to be “very” or “extremely hateful,” and no more than 52% prefer it to be deleted from the platform. The consensus we find in the data is rather that those factors systematically and strongly drive perceptions and preferences.

We also find another content-related feature to matter, which is the subject of hate speech: Posts directed toward women and Muslim immigrants are consistently perceived as more offensive and hateful than content directed toward partisan camps, and they trigger more support for platform action and other consequences. On average, perceived hatefulness increases by 7scale points for posts targeted at Muslim immigrants (b=0.07; 95% CI = 0.06–0.08; P<0.001; d.f.=18,057; $2\log_{e}\;[\text{BF}]=301$) and by 4scale points for posts targeted women (b=0.04; 95% CI = 0.03–0.04; P<0.001; d.f.=18,063; $2\log_{e}\;[\text{BF}]=81$) as compared to posts targeted at Conservatives. Furthermore, there is evidence for moderate effects of the addressing scope, with unrestricted scopes (“All <group>”) being perceived as more hateful than the sender addressing the target directly (“You”) (b=0.03; 95% CI = 0.02–0.04; P<0.001; d.f.=18,523; $2\log_{e}\;[\text{BF}]=45$).

Looking into context-related attributes, we find them to have remarkably little influence on people’s perceptions and preferences. There is positive to strong evidence that none of the identity-related sender and target characteristics affect the outcomes ($2\log_{e}\;[\text{BF}]<-4$ for all identity features). We do find a moderate increase in participants’ preferences for platform action when the target requests such action (b=0.04; 95% CI = 0.02–0.05; P<0.001; d.f.=18,328; $2\log_{e}\;[\text{BF}]=17$) or engages in counter-aggression (b=0.03; 95% CI = 0.02–0.04; P<0.001; d.f.=18,328; $2\log_{e}\;[\text{BF}]=8$), but have strong evidence for a null effect of the target appealing to norms ($2\log_{e}\;[\text{BF}]<-7$).

In the analysis of respondent characteristics, two robust patterns emerge (Fig. S18): Women (vs. men) as well as people who ideologically self-identify as left or center (vs. right) are on average more likely to perceive posts as more offensive and hateful and to support action against the controversial posts. On average, perceived hatefulness is 3scale points (b=0.03; 95% CI = 0.02–0.04; P<0.001; d.f.=2,137; $2\log_{e}\;[\text{BF}]=11$) higher for women than for men, and 6scale points (b=0.06; 95% CI=0.05–0.08; P<0.001; d.f.=2,103; $2\log_{e}\;[\text{BF}]=46$) higher for participants located ideologically left vs. right. Importantly, the gender difference is not driven by the vignettes displaying speech against women; the difference remains robust across topics (see Table S30). In addition, for the US sample we compare respondents identifying as white vs. nonwhite and find that nonwhites tend to have a stronger preference for platform and other sanctions, in particular when Muslim immigrants and women are targeted (Table S36; Fig. S42). Lacking racial markers for the German sample, we cannot perform similar analyses there. Also, we are not sufficiently powered to explore patterns for intersectional subgroups (e.g. black women), which is a limitation given their heightened risk of facing harassment online (56).

Furthermore, we find that evaluations are dependent on the interaction between topic statements and respondents’ ideology (Table S33; Fig. S39). On average, left-leaning respondents perceive messages targeted at women, Muslim immigrants, and the political Left (but not the political Right) to be more hateful than right-leaning respondents. Importantly, the overall differences between left- and right-leaning respondents are not entirely driven by our topic selection, which is biased towards conservative talking points: perceived hatefulness remains significantly lower on average for right-leaning respondents when the sample is restricted to insulting and violent statements—statements where the content of the message no longer has any substance, and where we would expect positional alignment to matter less (Fig. S22).

Other respondent characteristics are not as clearly correlated with either perceptions or preferences. We have positive to strong evidence of null effects for all other characteristics in the pooled sample ($2\log_{e}\;[\text{BF}]<-2$). Counter-intuitively, those who report having been target of hate speech themselves or having witnessed acts of hate speech online are no more likely to opt for sanctions. Overall, the effects look similar in both country samples, with somewhat more pronounced differences between ideological camps in the US sample. Respondents who identify with right-populist parties (AfD in Germany, Republicans in the United States) exhibit reduced perceptions of hatefulness and are significantly less likely to opt for platform and other sanctions.

In order to test for in- and out-group bias, we study interactions between respondent and sender/target characteristics, focusing on gender and ideology. While we do not find evidence for gender bias (Table S24), there is evidence for bias related to ideological cues. In line with our expectations, respondents sharing their ideological identity with the target but not with the sender perceive attacks as relatively more hateful than if their identity is different from the target. Our model predicts a 10scale-points (b=0.10; 95% CI=0.03–0.16; P<0.01; d.f.=579) difference on the hatefulness scale for ideologically left vs. right respondents, holding the target/sender ideology constant (Table S27). As with the main effects, the conditional ideology effects are more pronounced in the US sample and insignificant in the German sample (although the effect direction is as expected; see Tables S28 and S29). See Supplementary material D.2 for the full results disaggregated by outcome for Experiment 1 as well as for Bayes factors for all effects.

### Experiment 2: framing effects

We next discuss results from the framing experiment. Figure S43 summarizes the treatment effects of the free speech advocacy and government regulation frames vs. the neutral frame on respondents’ perceptions and preferred platform-side and other action. Also see Supplementary material D.4 for a tabular overview of results as well as Bayes factors for the individual effects.

In contrast to our expectations, both frames (instead of just the free speech advocacy frame) reduced perceptions of offensiveness ($B_{\mathrm{Pooled}}^{\mathrm{Freespeech}}=-0.03\;\text{scale points}$; 95% CI = −0.04 to −0.01; P<0.001; d.f.=2,173; $2\log_{e}\;[\text{BF}]=6$ and $B_{\mathrm{Pooled}}^{\mathrm{Gov}.\mathrm{reg}}=-0.03\;\text{scale points}$; 95% CI = −0.05 to −0.02; P<0.001; d.f.=2,180; $2\log_{e}\;[\text{BF}]=10$) and hatefulness ($B_{\mathrm{Pooled}}^{\mathrm{Freespeech}}=-0.02\;\text{scale points}$; 95% CI = −0.04 to −0.01; P<0.01; d.f.=2,174; $2\log_{e}\;[\text{BF}]=1$ and $B_{\mathrm{Pooled}}^{\mathrm{Gov}.\mathrm{reg}}=-0.03\;\text{scale points}$; 95% CI = −0.04 to −0.01; P<0.01; d.f.=2,182; $2\log_{e}\;[\text{BF}]=3$). One possible explanation for this pattern could be that, regardless of whether the frame introduced hate speech or censorship as a problem, both made people aware of hate speech in the public discourse, making the shown posts seem less extreme.

Moving to the preferences for action, most of the effects are not statistically significantly different from zero, and only one survives *P*-value adjustment for multiple testing: We do find evidence in the US sample for a positive effect of the free speech frame on the preference for no platform action ($B_{\mathrm{USsample}}^{\mathrm{Freespeech}}=0.07\;\text{scale points}$; 95% CI = 0.03–0.12; P<0.01; d.f.=974; $2\log_{e}\;[\text{BF}]=3$). We do not find evidence for effects of the free speech frame in the German sample ($2\log_{e}\;[\text{BF}]<-6$ across all actions), and no evidence for effects of the government regulation frame in either of the samples ($2\log_{e}\;[\text{BF}]<-8$ across all actions). The results from this experiment are very similar when the sample is reduced to those who pass the predefined attention checks (see Fig. S44) or when no covariates are used (see Fig. S45). In the Fig. S49, we also explore heterogeneous effects by a variety of preregistered covariates, but do not find evidence for substantial heterogeneity.

### Experiment 3: consequences of hate speech exposure

Finally, we consider the effects of hate speech exposure on attitudes toward free speech and censorship norms as well as support for controversial opinions. Figure S50 reports the estimated effects by sample. Respondents who were first shown the vignettes and then asked to state their support of the listed statements were on average $B_{\mathrm{Pooled}}^{\mathrm{Exposure}}=-0.08\;\text{ percentage points}$ (95% CI = −0.12 to −0.04; P<0.001; d.f.=1,909; $2\log_{e}\;[\text{BF}]=12$) less likely to support the statement “People should be able to express unpopular opinions publicly, even if others find those opinions deeply offensive.” Support for the statement “It is important that people can use the internet without government censorship” was $B_{\mathrm{Pooled}}^{\mathrm{Exposure}}=0.04\;\text{ percentage points}$ (95% CI = −0.07 to −0.00; P<0.05; d.f.=1,909; $2\log_{e}\;[\text{BF}]=-3$) lower under the exposure condition. We do not find evidence for significant effects on support for the other two items in the pooled sample. However, there is a striking difference between both country samples: The dampened support for the no-censorship norm is exclusively driven by the German sample, for which we also find a significant reduction in the proportion of respondents supporting the anti-Muslim statement ($B_{\mathrm{Germansample}}^{\mathrm{Exposure}}=-0.05\;\text{ percentage points}$; 95% CI = −0.09 to −0.01; P<0.05; d.f.=980; $2\log_{e}\;[\text{BF}]=6$). While we are not well-powered to test for effect heterogeneity across respondent characteristics and the framing condition, we provide preliminary evidence in Fig. S54, which does not indicate substantial differences between subgroups. See Supplementary material D.5 for a tabular overview of all results as well as Bayes factors for the individual effects.

Besides those experimental findings, the sequential order of evaluated vignettes provides another opportunity to explore the effects of hate speech exposure. Consistent with previous evidence on desensitization through hate speech exposure (19, 20), we find that perceived offensiveness and hatefulness is lower for the 2nd through 8th shown vignettes compared to the first, whereas the propensity to delete the post increases (see Fig. S21).

In sum, the results suggest that exogenous exposure to offensive and hateful speech can reduce tolerance of unpopular and offensive opinions and make an internet without government censorship less attractive. This contrasts our observational finding from above where we do not find evidence for a significant effect of reported hate speech exposure on perceptions and regulation preferences.

## Discussion

Free expression and its regulation have always been controversial (57). Current practices of online content moderation and regulation have fueled a debate in which different worldviews regarding the potential harm of speech and its suppression collide (58, 59). As a fundamentally normative issue, the moderation of speech is not a problem that is easily solved by technocratic means. At the same time, there is pressure on regulators and platforms to mitigate the negative consequences of hate speech and to protect public health and discourse. Our study aims to inform the debate with empirical evidence on public preferences on the issue. By exposing participants to concrete acts of speech and putting them into a moderator-style role, we shed light on how certain aspects of speech—both content-specific and contextual—structure public preferences.

Taken together, our results indicate that the type and severity of hateful speech matter for people’s perceptions and preferences. Untrained respondents are able to make nuanced decisions and seem to apply heuristics that are consistent with theoretical accounts of speech types and their severity. Speech perceptions and preferred sanctions are meaningfully related: Content that is perceived as more hateful or offensive also elicits support for more severe sanctions (Fig. S11), a finding that is consistent with other recent evidence (16, 17). More generally, we find that the content much more than the context of speech (i.e. who speaks to whom and whether this triggers a reaction) matters for it to be perceived as hateful or to trigger preferences in favor of sanctions (18). On the other hand, speech against threatened and marginalized groups (women, Muslim immigrants) is perceived as more problematic than speech against political partisans. In addition, we find evidence for in-group/out-group bias on ideological features: Respondents in the US sample were more sensitive towards hate speech against and more tolerant towards hate speech distributed by their ideological in-group.

At the respondent level, we find women to perceive the messages as more hateful and right-leaning respondents to perceive them as less so. These findings are consistent with experimental evidence on hate speech restriction preferences in the United States and Denmark (17) and on moderation of misinformation on social media (60, 61). Going beyond existing evidence, we find that, in the short run, exposure to hateful speech in an arbiter scenario makes people less tolerant of extreme views and also tends to lower support for a censorship-free internet, while at the same time exerting desensitizing effects (19, 20).

While the effects are not substantial in size (*Cohen’s d* consistently below 0.25), we argue that they are meaningful because they relate to fundamental values and highlighting the potential for exposure to offensive content to gradually erode the foundations of open expression.

The United States and Germany are two relevant and important cases to study. But future research should cast a wider net, encompassing a diverse range of cultures and countries, in particular the Global South. We also did not consider satire, memes and other visual content, limited the depth of the fictitious conversations, and focused only on four topics. Future research should consider additional forms and contents of speech to assess the robustness of our results.

Although our experimental setting makes the displayed social media posts more likely cases for consensus, we observe considerable variation of what people think should be done with them, in particular when they are not of the most extreme form. Real speech online is much more complex to detect and moderate. Taken together, this casts doubt on the potential for automation. If human judgments are not consistent, it is problematic to use them as a gold standard for automated moderation at scale, highlighting the severity of the challenge of content moderation.

Automation aside, our results have direct relevance to policy debates as well as to regulatory frameworks designed to make platforms’ content policies accountable to the public. The case of the Digital Services Act (DSA) in the European Union illustrates this: Since some details of its implementation will be determined at the national level, policymakers will need to pay close attention to the contours of public opinion in member states. As our results indicate, Germans are somewhat more supportive than Americans of punishing users for hateful or violent posts, and there may be further variation within the EU. Empirical evidence is therefore needed to continually inform the application of laws such as the DSA and the Online Safety Bill in the United Kingdom. Finally, our research suggests the possibility of unintended consequences of proposed laws, such as those in the US states of Texas and Florida, designed to prevent platforms from removing or downranking content. If the effect of such policies would be to confront users with more hate speech, this could result in greater support for censoring such content.

## Materials and methods

The study was preregistered at EGAP (https://egap.org/registration/5944). Data on the experiments were not made accessible to the researchers before the publication of the preanalysis plan. In analyzing the data, we deviated from the preanalysis plan in some aspects, which we report in Supplementary material.

### Sampling and participants

The Pulse panel is a subset of YouGov’s traditional survey panels, where respondents opt in to install tracking software on their devices. This happened independently of the recruitment into our study. In both surveys, panelists that installed the web tracking software RealityMine on their computers and cell phones agreed to participate in a “Politics and Media” study with multiple survey waves. Their voluntary participation was rewarded using YouGov’s proprietary point system.

For both samples, respondents were selected from YouGov’s large online participant panel according to demographic/political targets. The US sampling frame was based on the US Census Bureau’s 2016 American Community Survey 1-year sample. Weights were estimated using propensity scores which were then poststratified on presidential vote choice, gender, race (four categories), age (four categories), and education (four categories). The German sampling frame was based on the Best for Planning (B4p) study (62), and target marginals were matched on gender, age (four categories), and education (four categories). The German sample was refreshed before wave 5 of the study with 1,203 new participants to compensate for attrition, as wave 1 had been launched in June 2017 and thus about a year earlier than with the US sample. See Table S11 for descriptive statistics of main respondent covariates in both samples. The sample sizes used in this study were 1,232 (United States) and 1,390 respondents (Germany), respectively.

### Vignette experiment (experiment 1)

The construction of the vignette universe involves creating all combinations of attribute levels and removing illogical or implausible ones. Artificial variation is minimized by discarding duplicates differing only in sender names and applying rules such as ensuring the sender and target are different people and maintaining out-group dynamics in hate speech scenarios. See Tables S1 to S8 for an overview of all message modules by topic and sample, and Tables S9 and S10 for an overview of sender and target identities. The final dataset contains 40,960 unique vignettes. Vignette decks of eight vignettes each are then formed with balanced distributions of attributes like topic, sender gender, religion, and message category. Female and Muslim senders are initially oversampled to ensure the sender represents an out-group, and decks are filtered to maintain diversity in avatars, resulting in a balanced sample for analysis (see Figs. S5 and S6). The procedure to construct the vignettes is described in detail in Supplementary material.

We use a set of questions that respondents answer for each vignette. To measure *perceived offensiveness*, we ask: “Looking at the post marked with a red arrow, what do you think, how offensive is this post?” The answering options are (i) Extremely offensive, (ii) Very offensive, (iii) Somewhat offensive, (iv) Not very offensive, and (v) Not offensive at all. *Perceived hatefulness* is measured analogously replacing “offensive” with “hateful.” Next, respondents are asked what *consequences the posts or the author of the post should face*: “What actions should be taken by the platform providers? Select all that you find appropriate in this case.” Options were (i) No action should be taken, (ii) The post should be deleted, (iii) The sender of the message should be blocked from posting to the target of this message, (iv) The sender of the message should be temporarily banned from the platform, and (v) The sender of the message should be permanently banned from the platform. Furthermore, we ask: “What other actions should be taken? Select all that you find appropriate in this case.” Options were (i) No further action should be taken, (ii) The sender of the message should lose his/her job, (iii) A fine should be forced on the sender of the message, and (iv) A prison sentence should be forced on the sender of the message.

### Framing experiment (experiment 2)

The vignettes were preceded by an introduction that described the evaluation task. Respondents were randomly assigned to one of three versions which framed the task differently. We achieved balance on the marginal distributions of our set of pretreatment respondent covariates across all three conditions (see Table S37). The *neutral* version of the task was worded as follows: “In the following, you will see a couple of messages posted online by social media users. Some of these messages, marked with a red arrow, are potentially problematic. We want you to take a close look at these messages and then answer a few questions. We also want to point out that the contents of some of these messages may be unpleasant or repugnant to you. If you do not want to see any more such messages, you can skip these questions without answering.” The *government regulation* frame added the following text to the beginning of the neutral version: “As you may have heard, the government is serious about tackling online hate speech. Potential victims of online hate speech should be protected. This means that a large number of social media messages containing offensive or hateful content will be deleted and prosecuted.” The *free speech advocacy* frame added the following text to the beginning of the neutral version: “As you may have heard, civil society organizations are struggling to counter censorship of content on the Net. The right to freedom of expression should be protected. This means that social media messages containing offensive or hateful content should not be deleted or prosecuted.”

To check whether respondents actually take the time to carefully read the fictitious hate speech law, we ran an attention check just before the experimental manipulation (63). In addition, we used an alternative attention check (not preregistered) based on the time spent on the vignette introduction. Passing this check was defined as spending at least 10 s on the neutral condition or at least 15 s on one of the frame treatment conditions. In Supplementary material, we contrast the results of the framing experiment between the full sample and the reduced sample of respondents who passed either of the attention checks, showing no substantive differences (Fig. S44).

### Exposure experiment (experiment 3)

To investigate the potential downstream consequences of hate speech exposure, we coupled the vignette experiment with a split-half before-and-after design. Half of the sample was asked to express their support or opposition towards a set of four issues directly before the vignette experiment, the other half got this task after the vignettes. We achieved balance on the marginal distributions of our set of pretreatment respondent covariates across the two conditions (see Table S41). The issue attitudes were queried as follows: “Here you can find several statements made on social media that some people support while others oppose. Do you support or oppose these statements?” The individual statements were: (i) “People should be able to practice their religion freely in our country.” (ii) “Muslims out of United States. Protect the American People!” (iii) “It is important that people can use the Internet without government censorship.” (iv) “People should be allowed to express unpopular opinions in public, even those that are deeply offensive to other people.” People could state whether they (i) oppose or (ii) support each of those statements.

### Measurement of covariates

*Age* is measured continuously in years, but has been coarsened for the analyses into four categories (18–29 years, 30–49 years, 50–69 years, 70+ years). *Gender* is measured using a binary variable (male = 0, female = 1). *Education* is encoded in three categories (low = [did not finish school (yet), or finished school but holds no qualification to pursue education to satisfy university entrance requirements], intermediate = [finished school with qualification to pursue further education to satisfy university entrance requirements], high = [finished school achieving university entrance requirements, and/or holds university degree and/or postgraduate degree]). *Political interest* is measured on a five-point scale (from 0 = no interest at all to 4 = very much interest) and has been coarsened into three categories (low, medium, high), combining the two lowest and highest categories into one. *Political ideology* is measured on a five-point scale (from 0 = very liberal to 4 = very conservative) in the US survey and on an eleven-point scale (from 0 = very left to 10 = very left) in the German survey. We map both scales onto a three category scheme (United States: “very liberal”/“liberal” → liberal/left, “moderate” → moderate/center, and “conservative”/“very conservative” → conservative/right; Germany: 0–3 → liberal/left, 4–6 → moderate/center, 7–10 → conservative/right). *Party identification* is measured using three categories in the United States (Democrat, Republican, Independent; participants who answered “Other” and “Not sure” are excluded from the analysis) and using reported party vote intentions in Germany (CDU/CSU, SPD, Greens, FDP, Left, AfD, Others). *Personal experience with hate speech* is measured using a binary indicator (“I have personally been verbally attacked with hate speech online”; no = 0, yes = 1). *Witnessing hate speech* is measured using a binary indicator (“I have experienced how others have been verbally attacked with hate speech online.”; no = 0, yes = 1). *Feeling towards discussing politics with others* is measured using a binary indicator (0 = “I don’t feel free to discuss politics with anyone” or “I feel free to discuss politics with only a few people,” 1 = “I feel free to discuss politics with most people” or “I feel free to discuss politics with anyone”; observations agreeing with statement “I never discuss politics with other people” are disregarded in the analysis). *Social media usage* is measured with a binary indicator taking the value 1 if the respondent reports to have an account on any of the platforms Twitter, Facebook, Instagram, Reddit, or Snapchat, otherwise 0. All survey instruments used in this study are documented in Section E of Supplementary material.

### Data handling

“Don’t know” responses were considered missing data for our outcome measures. Missing covariates were treated as missing. We performed list-wise deletion in our analyses. Following the recommendation by Ref. (64), we analyzed the experiments without survey weights.

### Statistical analysis of the vignette experiment

To analyze our vignette survey experiment, we use hierarchical linear modeling with person, vignette deck, and country random effects (in pooled analyses). By including varying intercepts for individual respondents, we accommodate the nesting of vignette judgments within individuals, producing meaningful standard errors. Random intercepts for decks help us account for any deck-specific effects. Using a linear model specification for binary outcomes (action chosen = 1 vs. action not chosen = 0) yields a straightforward interpretation of coefficients in terms of probabilities of agreeing with the perception or treatment of controversial or hate speech. To test for the moderating effect of respondent characteristics on hate speech regulation preferences, we interact the relevant attributes with respondents’ background characteristics. In addition, we test for subgroup differences by both interacting pretreatment covariates with relevant attributes and reporting mean differences in the outcome variables by subgroup. We also provide Bayes factors assessing the pairwise ratio of marginal likelihoods for two models, one of which is specified with the predictor of interest and the other one without (which is the baseline model). We present twice the natural logarithm of the Bayes factor ($2\log_{e}\;[\text{BF}]$), which is on the same scale as a likelihood ratio test statistic (65), and which we use to interpret the evidence as follows: (BF≤−10)= very strongly in favor of the Null; (−10>BF≤−6)= strongly in favor of the Null; (−6>BF≤−2)= positively in favor of the Null; (−2>BF≤0)= weakly in favor of the Null; (0>BF≤2)= weakly against the Null; (2>BF≤6)= positively against the Null; (6>BF≤10)= strongly against the Null; (BF>10)= very strongly against the Null. We do this for all analyses presented in the main text (not preregistered). A formal power analysis suggests that formal, given our sample size, we are able to detect AMCEs as small as 0.05 with conventional power of 0.8 and an alpha of 0.05 (see Supplementary material).

### Statistical analysis of the framing experiment

To test the hypotheses on the role of governmental and civil action for hate speech preferences, we include the treatment conditions as dummy variables in the linear hierarchical models and use P<0.05 as a criterion for statistical significance after controlling for the false discovery rate (66). We run models both with and without vignette and respondent characteristics. We also explore potential interaction effects between the framing dummies and vignette or the respondent characteristics mentioned above to test for potential causal heterogeneity.

### Statistical analysis of the exposure experiment

To test the hypotheses on the downstream consequences of hate speech exposure, we rely on both simple differences-in-means and OLS estimators to compare the answers given before and after the vignette task (note that the question order is randomized, yielding a between-subjects design). We look at the four stated preference items separately and use P<0.05 as a criterion for statistical significance after controlling for the false discovery rate. For the OLS estimator, we run models both with and without respondent characteristics. We also explore potential interaction effects between a question order dummy and the respondent characteristics mentioned above to test for potential causal heterogeneity.

### Consent and ethics

For the data analyzed in this study, we followed a strict protocol informed by IRB guidance and best practices (67). This study was approved by the Institutional Review Boards of Princeton University (protocols 8,327, 10,014, and 10,041) and the University of Southern California (UP-17-00513) and authorized by the University of Illinois via a designated IRB agreement.

Sharing both the survey data and web visits with researchers is done with informed consent. People who join the Pulse panel are told about the nature of the data collected, that it is kept anonymous, and that data are not shared with third parties. Before joining this specific study, respondents additionally agreed to a separate consent statement informing them, “Your participation is voluntary. Participation involves completion of a short survey and voluntary tracking of online media consumption. You may choose not to answer any or all questions. Furthermore, you are free to opt out of web tracking, which you may have previously agreed to participate in as part of the YouGov Pulse panel, at any time.”

Our study design is based on the principle that understanding individuals’ perceptions and preferences requires observing judgments of actual cases, which inherently involves presenting realistic examples of such content. To address potential harms for participants, we recognize two primary risks: psychological distress that may arise if participants, particularly those belonging to targeted groups, experience anxiety or discomfort, and the normalization of hate speech through repeated exposure, which carries a risk of desensitizing participants or reinforcing harmful attitudes. To mitigate these risks, we implemented several measures. Prior to exposing respondents to the set of hate speech vignettes, participants received a content warning and were provided an opt-out option, allowing them to skip questions containing potentially distressing material: “We also want to point out that the contents of some of these messages may be unpleasant or repugnant to you. If you do not want to see any more such messages, you can skip these questions without answering.” Furthermore, we avoided extreme content, such as graphic depictions of violence, and framed the messages within a context that highlighted their potentially problematic nature.

## Supplementary Material

pgaf032_Supplementary_Data

## Data Availability

The datasets used in this study are shared with permission of the data provider and in compliance with GDPR requirements at https://doi.org/10.4232/1.13979 (German data file) and https://doi.org/10.4232/1.13980 (US data file). Information that would have made subjects directly identifiable was deliberately withheld by the survey provider. In addition, we removed information that could potentially be used to indirectly identify subjects (including zip code). The computer code and processed data for this study is available at https://osf.io/mwe8h/?view_only=dce91ed546de42ebb2ac69c1659a164d.
