# Supplementary material for: Citizen preferences for online hate speech regulation
Source: PNAS Nexus. 2025 Feb 12;4(2):pgaf032. doi: 10.1093/pnasnexus/pgaf032 (PMC11840861; doi:10.1093/pnasnexus/pgaf032)
Supplement: pgaf032_Supplementary_Data [file pgaf032_supplementary_data.pdf]

Citizen Preferences for Online Hate Speech Regulation

Simon Munzert, Richard Traunmüller, Pablo Barberá, Andrew Guess, JungHwan Yang

Supplemental Material

Contents

A Pre-analysis plan and deviations **2**

B Vignette construction **4**

B.1 Vignette design and attributes 4

B.2 Construction of vignette universe 15

B.3 Construction of vignette decks 15

C Additional descriptive evidence **25**

D Additional results **34**

D.1 Statistical power of vignette design 34

D.2 Vignette evaluations, mixed-effects models 37

D.3 Vignette evaluations, interaction effects 63

D.4 Framing experiment 90

D.5 Exposure experiment 102

E Survey questionnaire **112**

E.1 Sociodemographic variables 112

E.2 Behaviors and attitudes related to hate speech 114

E.3 Instruments for vignette evaluations 118

E.4 Instruments for framing experiment 120

E.5 Instruments for exposure experiment 123

E.6 Other covariates 124

A Pre-analysis plan and deviations

This study was pre-registered on July 26, 2019, via EGAP. The pre-registration was submitted before the data was made available to the researchers by the data provider. The pre-registration as well as the accompanying pre-analysis plan are available under the following link: `https://osf.io/yvcdq`.

There were a few areas in which we had to deviate from the plan. For the sake of transparency, we report each of these changes here:

- Due to an error in the implementation of item order randomization in the German survey, which resulted in all respondents being shown the direct items after the vignettes, the experiment (along with the vignette experiment) was repeated in a new wave 9, which was fielded from 20 September 2020 to 14 October 2020. We use this wave for the evaluation of the exposure experiment in the German sample, and wave 8 for all other analyses.
- In the pre-analysis plan, we wrote: ”We will analyze the data separately for Germany and the US and test for differences between these two country contexts.” While we report our analyses both based on the pooled samples and separately by country, we do not test for differences between the countries, as this would inflate our reporting with a lot of unnecessary information that is not central for us to interpret the results.
- In the pre-analysis plan we wrote with respect to our analytic strategy of both the framing and the exposure experiment: ”We will adjust for multiple comparisons using a simple Bonferroni correction.” We do report results from multiple comparisons adjustments, but do not limit ourselves to the Bonferroni correction. Instead, we control for the false discovery rate within samples and model specifications.
- Contrary to what we stated in the pre-analysis plan, we do not consider religion and racial resentment as covariates, as they were not measured in both countries.
- In the pre-analysis plan, we wrote: ”We will also compare our results to the more common strategy of calculating average marginal component effects (AMCE) using linear regression with clustered standard errors.” We estimated such models but did not find any noticeably

differences in the results compared to the results from the hierarchical models reported in the paper. We refrain from reporting these additional results in order to not overload the appendix with more results which provide little further insight.

- In the pre-analysis plan, we wrote: "We plan to publish the results in two papers." We ultimately decided to publish the results in one paper.

B Vignette construction

B.1 Vignette design and attributes

The vignettes are constructed in a way that mimics posts on a popular social media platform (here: Facebook). Irrelevant features of the message, such as time stamp or features to interact with it, are dropped. Only features that represent relevant attributes of the vignettes are kept. These attributes cover issues, sender as well as target characteristics, and sender message's and target message's characteristics.

Tables S1 to S4 provide the detailed components of the messages across the four different issues/target groups (Muslim immigrants, women, ideological Left, ideological Right) for the US survey. Tables S5 to S8 provide the same information for the German survey. Tables S9 and S10 provide information on sender and target characteristics. The prenames and surnames were chosen based on lists of popular female and male, Muslim and non-Muslim names in the United States and Germany, respectively. The thumbnail images were taken from stock photo platforms published under creative commons licences.[1] For the non-Muslim group, only whites were used. For the Muslim group, mostly people with dark complexion and, in part, headgear, were used. It is important to note that we use the visual and name characteristics as deliberately suggestive cues to implicitly signal in-group or out-group membership for the groups "Muslim", "non-Muslim", "woman", and "no woman". Also, we use two senders/targets who are anonymous (no gender or religion cues) but provide liberal and conservative cues. The liberal anonymous account is "Team Global", featured with the rainbow flag. The conservative anonymous account is "Team USA" featured with the US flag ("Team Deutschland" with German flag in the German survey).

Figures S1 and S2 provide a random sample of vignettes used (the label IDs were not shown to the respondents).

[1]Licences and authors: CC0, CC BY 2.0, CC BY-SA 2.0, Adam Jones, Rod Waddington, PrinceMedia, stux, Russell Watkins.

Table S1: Muslim immigrant vignettes: message components, US survey

Target message	Addressing scope	Sender message	Target reply
I'm a proud Muslim immigrant! [identification]	Most Muslim immigrants	should be banned from coming into our country. [discrimination, moderate]	Please stop attacking others. [appealing to norms 1]
I support Muslim immigrants. [support]	All Muslim immigrants	should be deported. [discrimination, extreme]	Please stay civil. We all have far more in common with each other than things that divide us. [appealing to norms 2]
	Extremist Muslim immigrants	should be punched in the face. [violence, moderate]	You are so dumb! [counter-aggression 1]
	You	should be killed. [violence, extreme]	Shut up asshole! [counter-aggression 2]
		are dumb. [insult, moderate]	[target] has reported [sender]'s post. [platform action 1]
		are stupid animals. [insult, extreme]	[target] has blocked [sender]'s account. [platform action 2]
		are just different from us. [vilification, moderate]	none
		are genetically inferior to us. [vilification, extreme]	

Table S2: Women vignettes: message components, US survey

Target message	Addressing scope	Sender message	Target reply
I'm a proud feminist! [identification]	Most women	should not be allowed to serve in the army. [discrimination, moderate]	Please stop attacking others. [appealing to norms 1]
I support feminism. [support]	All women	should be caring mothers and not pursue a selfish career. [discrimination, extreme]	Please stay civil. We all have far more in common with each other than things that divide us. [appealing to norms 2]
	Extremist feminist women	should be punched in the face. [violence, moderate]	You are so dumb! [counter-aggression 1]
	You	should be killed. [violence, extreme]	Shut up asshole! [counter-aggression 2]
		are dumb. [insult, moderate]	[target] has reported [sender]'s post. [platform action 1]
		are stupid animals. [insult, extreme]	[target] has blocked [sender]'s account. [platform action 2]
		are just different from men. [vilification, moderate]	none
		are genetically inferior to men. [vilification, extreme]	

Table S3: Ideological Left vignettes: message components, US survey

Target message	Addressing scope	Sender message	Target reply
I'm a proud Democrat! [identification]	Most Democrats	should be stopped from spreading falsehoods. [discrimination, moderate]	Please stop attacking others. [appealing to norms 1]
I support Democrats. [support]	All Democrats	should be not allowed to vote. [discrimination, extreme]	Please stay civil. We all have far more in common with each other than things that divide us. [appealing to norms 2]
	Socialist Democrats	should be punched in the face. [violence, moderate]	You are so dumb! [counter-aggression 1]
	You	should be killed. [violence, extreme]	Shut up asshole! [counter-aggression 2]
		are dumb. [insult, moderate]	[target] has reported [sender]'s post. [platform action 1]
		are stupid animals. [insult, extreme]	[target] has blocked [sender]'s account. [platform action 2]
		are just not as clever as Republicans. [vilification, moderate]	none
		are genetically inferior to Republicans. [vilification, extreme]	

Table S4: Ideological Right vignettes: message components, US survey

Target message	Addressing scope	Sender message	Target reply
I'm a proud Republican! [identification]	Most Republicans	should be stopped from spreading falsehoods. [discrimination, moderate]	Please stop attacking others. [appealing to norms 1]
I support Republican. [support]	All Republicans	should be not allowed to vote. [discrimination, extreme]	Please stay civil. We all have far more in common with each other than things that divide us. [appealing to norms 2]
	Trumpist Republicans	should be punched in the face. [violence, moderate]	You are so dumb! [counter-aggression 1]
	You	should be killed. [violence, extreme]	Shut up asshole! [counter-aggression 2]
		are dumb. [insult, moderate]	[target] has reported [sender]'s post. [platform action 1]
		are stupid animals. [insult, extreme]	[target] has blocked [sender]'s account. [platform action 2]
		are just not as clever as Democrats. [vilification, moderate]	none
		are genetically inferior to Democrats. [vilification, extreme]	

Table S5: Muslim immigrant vignettes: message components, German survey

Target message	Addressing scope	Sender message	Target reply
Ich bin ein stolzer muslimischer Einwanderer! [identification]	Die meisten muslimischen Einwanderer	sollten daran gehindert werden, in unser Land zu kommen. [discrimination, moderate]	Bitte höre auf, andere anzugreifen. [appealing to norms 1]
Ich unterstütze muslimische Einwanderer. [support]	Alle muslimischen Einwanderer	sollten abgeschoben werden. [discrimination, extreme]	Bitte bleibe höflich. Wir haben alle mehr gemeinsam, als uns voneinander trennt. [appealing to norms 2]
	Extremistische muslimische Einwanderer	sollten eine reingehauen bekommen. [violence, moderate]	Du bist so blöd! [counter-aggression 1]
	Du	sollten abgeknallt werden. [violence, extreme]	Halt's Maul Arschloch! [counter-aggression 2]
		sind Idioten. [insult, moderate]	[target] hat [sender]s Nachricht angezeigt. [platform action 1]
		sind dumme Kreaturen. [insult, extreme]	[target] hat [sender]s Account blockiert. [platform action 2]
		sind einfach anders als wir. [vilification, moderate]	none
		sind uns genetisch unterlegen. [vilification, extreme]	

Table S6: Women vignettes: message components, German survey

**Target message**	**Addressing scope**	**Sender message**	**Target reply**
Ich bin ein stolzer Feminist! [identification]	Die meisten Frauen	sollten fürsorgliche Mütter sein und keine egoistische Karriere verfolgen. [discrimination, moderate]	Bitte höre auf, andere anzugreifen. [appealing to norms 1]
Ich unterstütze Feminismus. [support]	Alle Frauen	sollten nicht in der Bundeswehr dienen dürfen. [discrimination, extreme]	Bitte bleibe höflich. Wir haben alle mehr gemeinsam, als uns voneinander trennt. [appealing to norms 2]
	Extrem feministische Frauen	sollten eine reingehauen bekommen. [violence, moderate]	Du bist so blöd! [counter-aggression 1]
	Du	sollten abgeknallt werden. [violence, extreme]	Halt's Maul Arschloch! [counter-aggression 2]
		sind Idioten. [insult, moderate]	[target] hat [sender]s Nachricht angezeigt. [platform action 1]
		sind dumme Kreaturen. [insult, extreme]	[target] hat [sender]s Account blockiert. [platform action 2]
		sind einfach anders als Männer. [vilification, moderate]	none
		sind genetisch Männern unterlegen. [vilification, extreme]	

Table S7: Ideological Left vignettes: message components, German survey

Target message	Addressing scope	Sender message	Target reply
Ich bin ein stolzes Mitglied der Grünen! [identification]	Die meisten Grünen	sollten davon abgehalten werden, Lügen zu verbreiten. [discrimination, moderate]	Bitte höre auf, andere anzugreifen. [appealing to norms 1]
Ich unterstütze die Grüne. [support]	Alle Grünen	sollten nicht wählen dürfen. [discrimination, extreme]	Bitte bleibe höflich. Wir haben alle mehr gemeinsam, als uns voneinander trennt. [appealing to norms 2]
	Linksextreme Grüne	sollten eine reingehauen bekommen. [violence, moderate]	Du bist so blöd! [counter-aggression 1]
	Du	sollten abgeknallt werden. [violence, extreme]	Halt's Maul Arschloch! [counter-aggression 2]
		sind Idioten. [insult, moderate]	[target] hat [sender]s Nachricht angezeigt. [platform action 1]
		sind dumme Kreaturen. [insult, extreme]	[target] hat [sender]s Account blockiert. [platform action 2]
		sind einfach nicht so schlau wie AfDler. [vilification, moderate]	none
		sind AfDlern genetisch unterlegen. [vilification, extreme]	

Table S8: Ideological Right vignettes: message components, German survey

Target message	Addressing scope	Sender message	Target reply
Ich bin ein stolzes Mitglied der AfD! [identification]	Die meisten AfDler	sollten davon abgehalten werden, Lügen zu verbreiten. [discrimination, moderate]	Bitte höre auf, andere anzugreifen. [appealing to norms 1]
Ich unterstütze die AfD. [support]	Alle AfDler	sollten nicht wählen dürfen. [discrimination, extreme]	Bitte bleibe höflich. Wir haben alle mehr gemeinsam, als uns voneinander trennt. [appealing to norms 2]
	Rechtsextreme AfDler	sollten eine reingehauen bekommen. [violence, moderate]	Du bist so blöd! [counter-aggression 1]
	Du	sollten abgeknallt werden. [violence, extreme]	Halt's Maul Arschloch! [counter-aggression 2]
		sind Idioten. [insult, moderate]	[target] hat [sender]s Nachricht angezeigt. [platform action 1]
		sind dumme Kreaturen. [insult, extreme]	[target] hat [sender]s Account blockiert. [platform action 2]
		sind einfach nicht so schlau wie Grüne. [vilification, moderate]	none
		sind Grünen genetisch unterlegen. [vilification, extreme]	

Table S9: Sender and target characteristics, US survey

Ideology	Religion cue	Gender	Prename	Surname	Thumbnail
unknown	Muslim	female	Fatima	Abad	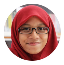
			Nazia	Karimi	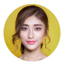
			Saba	Malek	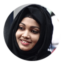
			Zainab	Omer	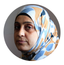
		male	Amir	Rahman	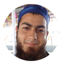
			Muhammad	Nazir	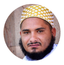
			Nadeem	Shakir	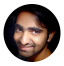
			Rashid	Farra	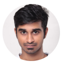
	non-Muslim	female	Anna	Krueger	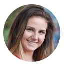
			Lisa	Mueller	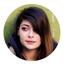
			Laura	Harris	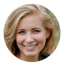
			Carolyn	Clark	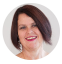
		male	Paul	Miller	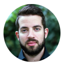
			Mark	Schmitt	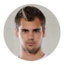
			Lucas	Baker	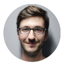
			Florian	Smith	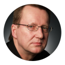
liberal	unknown	unknown	Team	Global	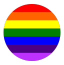
conservative			Team	USA	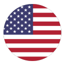

Table S10: Sender and target characteristics, German survey

Ideology	Religion cue	Gender	Prename	Surname	Thumbnail
unknown	Muslim	female	Fatima	Abad	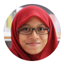
			Nazia	Karimi	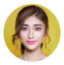
			Saba	Malek	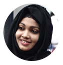
			Zeynep	Omer	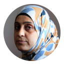
		male	Amir	Rahman	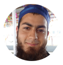
			Muhammad	Nazir	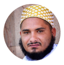
			Nadeem	Shakir	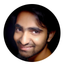
			Rashid	Farra	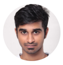
	non-Muslim	female	Anna	Schneider	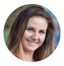
			Lisa	Meier	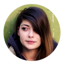
			Laura	Fischer	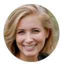
			Carolin	Weber	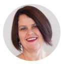
		male	Paul	Wagner	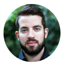
			Mark	Schmidt	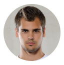
			Lukas	Becker	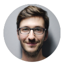
			Florian	Schulz	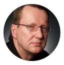
liberal	unknown	unknown	Team	Global	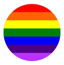
conservative			Team	Deutschland	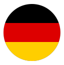

B.2 Construction of vignette universe

To construct the vignette universe, which is later used to sample from to generate the vignette decks, we create a data frame of all combinations of all attribute levels and later exclude observations that are illogical or implausible. The artificial variation across sender and message characteristics is reduced: For instance, in the case of two vignettes that are exactly equal but differ only on the sender's name (e.g., a female Muslim named Fatima Abad vs. a female Muslim named Nazia Karimi), one of the vignettes is randomly discarded. Furthermore, the following rules are implemented:

- Target and sender must be different persons.
- A sender message addressing the out-group target (e.g., a male target in hate speech addressing women or a non-Muslim target in hate speech addressing Muslims) directly ("You...") can only be a message of class *violence* or *insult*, not *discrimination* or *vilification.*
- If the message is addressing the target directly ("You..."), some messages have to be grammatically adapted (e.g., replace "You are stupid animals" with "You are a stupid animal").
- The sender has to be of type out-group. That is, in hate speech targeting Muslims, the sender cannot be Muslim, in hate speech targeting women, the sender cannot be female, in hate speech targeting the ideological Left, the sender cannot be ideologically left, and in hate speech targeting the ideological Right, the sender cannot be ideologically right.

Keeping only vignettes that comply with these rules gives us a set of 40,960 unique vignettes.

B.3 Construction of vignette decks

In the next step, we construct the vignette decks, which consist of eight individual vignettes each. The goal is to achieve approximate balance of all attribute levels in the sample of vignettes used in the surveys, to avoid repeated use of persons in individual decks as often as possible, and to maximize variation of attribute levels in individual decks. To that end, we define the a priori distribution of features within single decks ("stratification"). The a priori distribution is:

- topic: 2 Muslim immigrant, 2 woman, 2 ideological Left, 2 ideological Right
- gender, sender: 3 male, 5 female
- religion, sender: 3 non-Muslim, 5 Muslim
- ideology, sender: 6 unknown, 1 conservative, 1 liberal
- target group category: 2 most, 2 all, 2 extreme, 2, you
- target message category: 4 proud, 4 support
- sender message category: 2 discrimination, 2 insult, 2 vilification, 2 violence
- target reply category: 2 appealing to norms, 2 counter-aggression, 2 platform action, 2 none

Note that, a priori, we over-sampled female and Muslim senders because they are later replaced with male or non-Muslim senders in women and Muslim immigrant vignettes (see rule above: sender has to be of type out-group).

Based on this distribution of features, we sample actual patterns of vignette sets. In the next step, we randomly draw from all those vignettes from the vignette universe that match the sampled patterns. These rules do not guarantee yet that the occurrence of sender/target character duplicates within individual decks is minimized. Therefore, we generate a gross sample of vignette decks and keep only those with 13 or more unique characters among senders and targets.

Overall, the described procedure generates a largely balanced representation of the attribute levels in the sample of vignette decks (see Figures S3 to S8).

Figure S1: Sample of vignettes

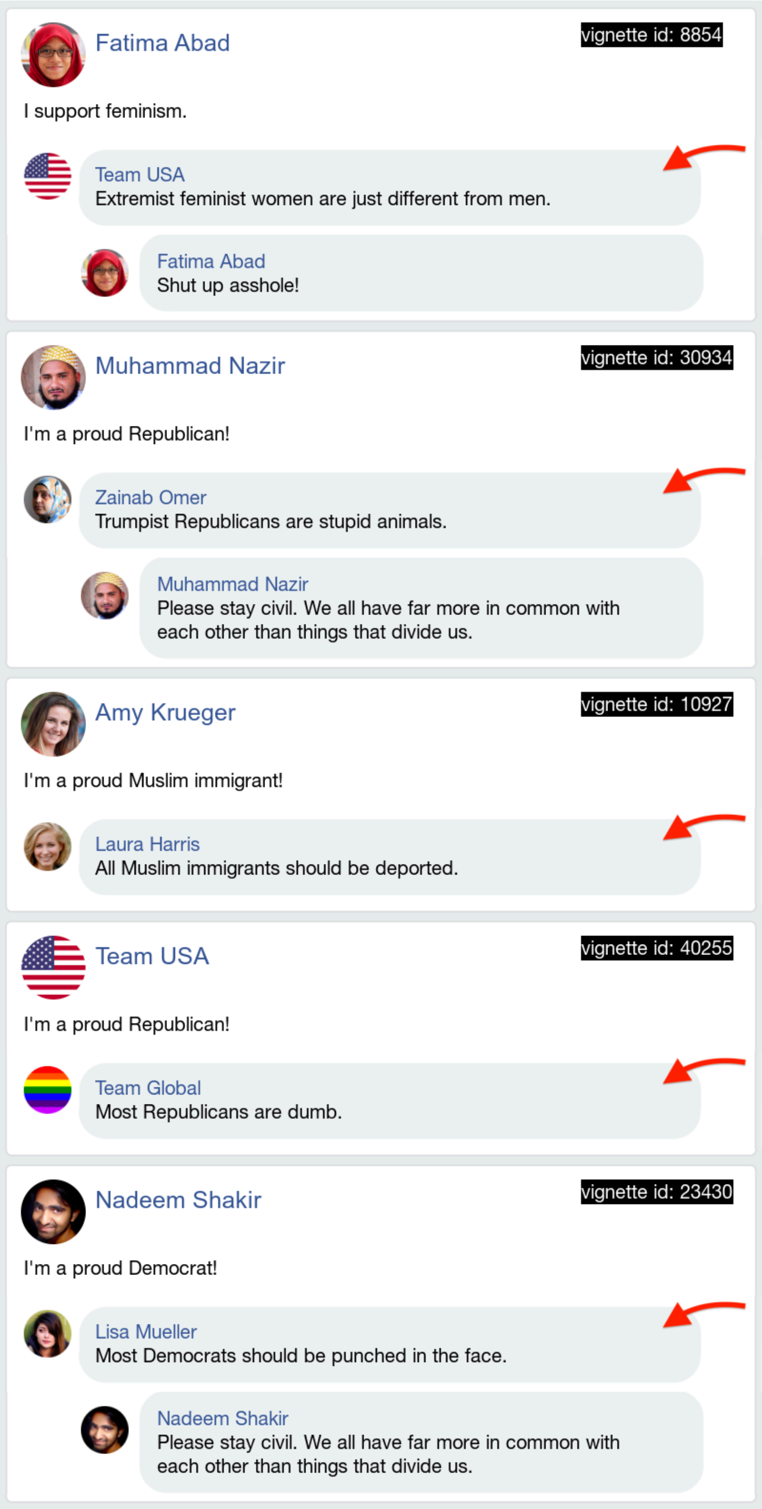

Figure S2: Sample of vignettes, *continued*

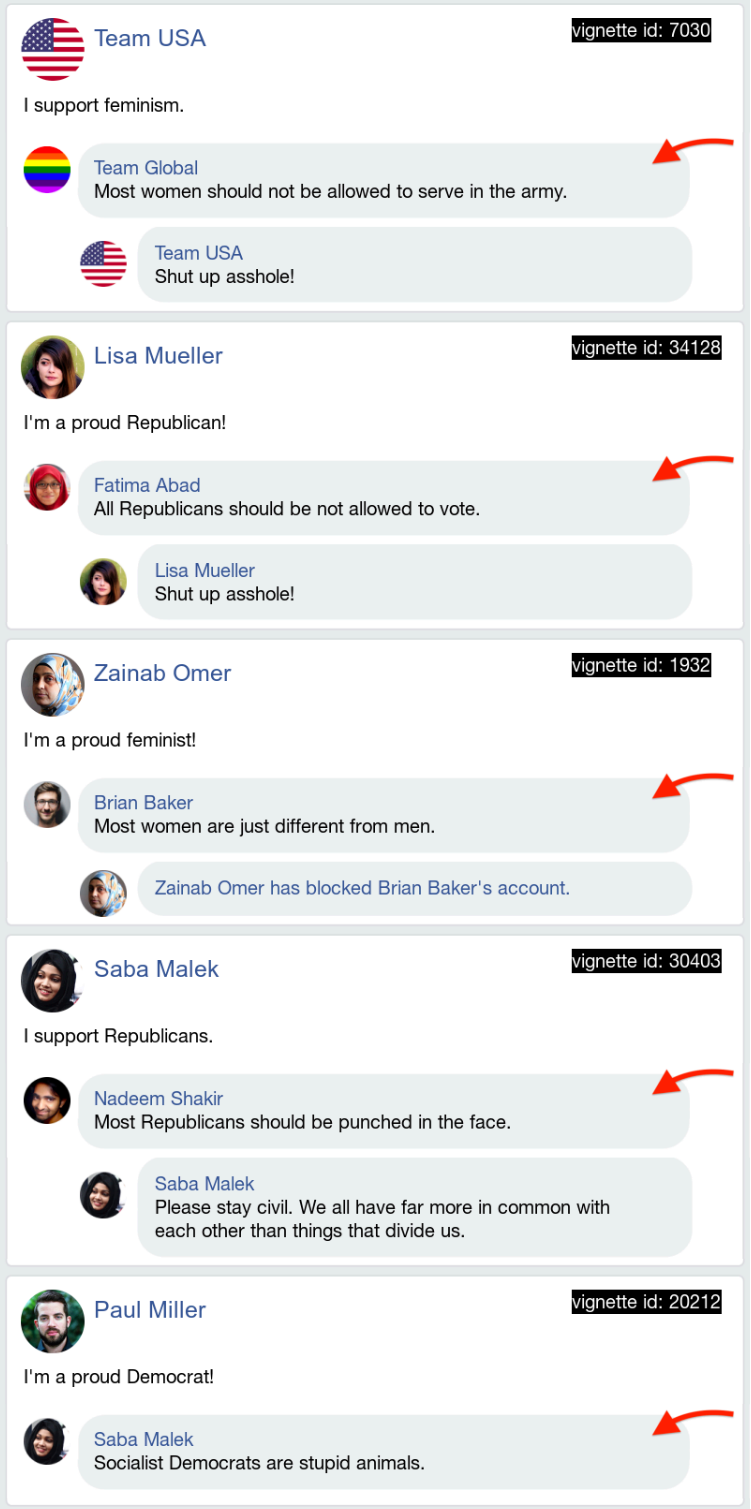

Figure S3: Balance of vignette attributes in gross sample (US survey)

No	Variable	Stats / Values	Freqs (% of Valid)	Graph	Valid	Missing
1	id<br>[integer]	mean (sd) : 18803.83 (12022.21)<br>min < med < max :<br>13 < 18443 < 40968<br>IQR (CV) : 20457.5 (0.64)	3031 distinct values	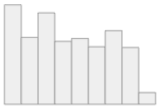	3200<br>(100%)	0<br>(0%)
2	topic<br>[character]	1. gender<br>2. ideologydems<br>3. ideologyreps<br>4. muslim	800 (25.0%)<br>800 (25.0%)<br>800 (25.0%)<br>800 (25.0%)	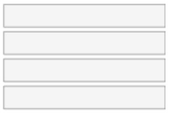	3200<br>(100%)	0<br>(0%)
3	gender_target<br>[character]	1. female<br>2. male<br>3. unknown	1195 (37.3%)<br>1205 (37.7%)<br>800 (25.0%)	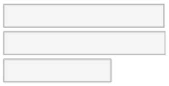	3200<br>(100%)	0<br>(0%)
4	religion_target<br>[character]	1. muslim<br>2. nonmuslim<br>3. unknown	1213 (37.9%)<br>1187 (37.1%)<br>800 (25.0%)	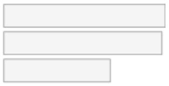	3200<br>(100%)	0<br>(0%)
5	ideology_target<br>[character]	1. conservative<br>2. liberal<br>3. unknown	402 (12.6%)<br>398 (12.4%)<br>2400 (75.0%)	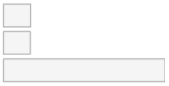	3200<br>(100%)	0<br>(0%)
6	name_target<br>[character]	1. Amir Rahman<br>2. Anna Schneider<br>3. Carolin Weber<br>4. Fatima Abad<br>5. Florian Schulz<br>6. Laura Fischer<br>7. Leyla Karimi<br>8. Lisa Meier<br>9. Lukas Becker<br>10. Mark Schmidt<br>11. Mohammed Nazir<br>12. Nadeem Shakir<br>13. Paul Wagner<br>14. Rashid Farra<br>15. Saba Malek<br>16. Team Deutschland<br>17. Team Global<br>18. Zeynep Omer	149 (4.7%)<br>118 (3.7%)<br>153 (4.8%)<br>162 (5.1%)<br>163 (5.1%)<br>137 (4.3%)<br>154 (4.8%)<br>153 (4.8%)<br>166 (5.2%)<br>151 (4.7%)<br>153 (4.8%)<br>154 (4.8%)<br>146 (4.6%)<br>123 (3.8%)<br>144 (4.5%)<br>402 (12.6%)<br>398 (12.4%)<br>174 (5.4%)	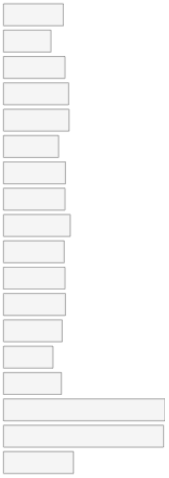	3200<br>(100%)	0<br>(0%)
7	gender_sender<br>[character]	1. female<br>2. male<br>3. unknown	1122 (35.1%)<br>1278 (39.9%)<br>800 (25.0%)	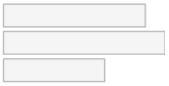	3200<br>(100%)	0<br>(0%)
8	religion_sender<br>[character]	1. muslim<br>2. nonmuslim<br>3. unknown	1121 (35.0%)<br>1279 (40.0%)<br>800 (25.0%)	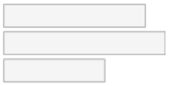	3200<br>(100%)	0<br>(0%)
9	ideology_sender<br>[character]	1. conservative<br>2. liberal<br>3. unknown	378 (11.8%)<br>422 (13.2%)<br>2400 (75.0%)	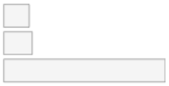	3200<br>(100%)	0<br>(0%)

Figure S4: Balance of vignette attributes in gross sample (US survey), *continued*

No	Variable	Stats / Values	Freqs (% of Valid)	Graph	Valid	Missing
10	name_sender<br>[character]	1. Amir Rahman<br>2. Anna Schneider<br>3. Carolin Weber<br>4. Fatima Abad<br>5. Florian Schulz<br>6. Laura Fischer<br>7. Leyla Karimi<br>8. Lisa Meier<br>9. Lukas Becker<br>10. Mark Schmidt<br>11. Mohammed Nazir<br>12. Nadeem Shakir<br>13. Paul Wagner<br>14. Rashid Farra<br>15. Saba Malek<br>16. Team Deutschland<br>17. Team Global<br>18. Zeynep Omer	162 (5.1%)<br>174 (5.4%)<br>168 (5.2%)<br>113 (3.5%)<br>167 (5.2%)<br>163 (5.1%)<br>117 (3.7%)<br>144 (4.5%)<br>153 (4.8%)<br>156 (4.9%)<br>165 (5.2%)<br>155 (4.8%)<br>154 (4.8%)<br>166 (5.2%)<br>117 (3.7%)<br>378 (11.8%)<br>422 (13.2%)<br>126 (3.9%)		3200<br>(100%)	0<br>(0%)
11	target_group<br>[character]	1. Alle AfDler<br>2. Alle Frauen<br>3. Alle Grünen<br>4. Alle muslimischen Einwanderer<br>5. Die meisten AfDler<br>6. Die meisten Frauen<br>7. Die meisten Grünen<br>8. Die meisten muslimischen Einwa<br>9. Du<br>10. Extrem feministische Frauen<br>11. Extremistische muslimische Ein<br>12. Linksextreme Grüne<br>13. Rechtsextreme AfDler	210 (6.6%)<br>218 (6.8%)<br>225 (7.0%)<br>217 (6.8%)<br>232 (7.2%)<br>211 (6.6%)<br>199 (6.2%)<br>206 (6.4%)<br>597 (18.7%)<br>200 (6.2%)<br>224 (7.0%)<br>227 (7.1%)<br>234 (7.3%)		3200<br>(100%)	0<br>(0%)
12	target_group_category<br>[character]	1. all<br>2. extremist<br>3. most<br>4. you	870 (27.2%)<br>885 (27.7%)<br>848 (26.5%)<br>597 (18.7%)		3200<br>(100%)	0<br>(0%)
13	target_message<br>[character]	1. Ich bin ein stolzer Feminist!<br>2. Ich bin ein stolzer muslimisch<br>3. Ich bin ein stolzes Mitglied d<br>4. Ich bin ein stolzes Mitglied d<br>5. Ich bin eine stolze Feministin<br>6. Ich bin eine stolze muslimisch<br>7. Ich unterstütze die Alternativ<br>8. Ich unterstütze die Grünen.<br>9. Ich unterstütze Feminismus.<br>10. Ich unterstütze muslimische Ei	249 (7.8%)<br>226 (7.1%)<br>422 (13.2%)<br>404 (12.6%)<br>145 (4.5%)<br>154 (4.8%)<br>378 (11.8%)<br>396 (12.4%)<br>406 (12.7%)<br>420 (13.1%)		3200<br>(100%)	0<br>(0%)
14	target_message_category<br>[character]	1. proud<br>2. support	1600 (50.0%)<br>1600 (50.0%)		3200<br>(100%)	0<br>(0%)

Figure S5: Balance of vignette attributes in gross sample (US survey), *continued*

No	Variable	Stats / Values	Freqs (% of Valid)	Graph	Valid	Missing
15	sender_message<br>[character]	1. sollten abgeknallt werden.<br>2. sind dumme Kreaturen.<br>3. sollten eine reingehauen bekom<br>4. sind Idioten.<br>5. sollten nicht wählen dürfen.<br>6. solltest eine reingehauen beko<br>7. bist eine dumme Kreatur.<br>8. bist ein Idiot.<br>9. solltest abgeknallt werden.<br>10. sind einfach nicht so schlau w<br>11. sollten abgeschoben werden.<br>12. sind AfDlern genetisch unterle<br>13. sind uns genetisch unterlegen.<br>14. sind einfach anders als wir.<br>15. sollten nicht in der Bundesweh<br>16. sind genetisch Männern unterle<br>17. sind einfach nicht so schlau w<br>18. sind Grünen genetisch unterleg<br>19. sollten davon abgehalten werde<br>20. sollten daran gehindert werde<br>[18 others]	380 (11.9%)<br>372 (11.6%)<br>362 (11.3%)<br>360 (11.2%)<br>149 (4.7%)<br>129 (4.0%)<br>126 (3.9%)<br>120 (3.8%)<br>111 (3.5%)<br>81 (2.5%)<br>81 (2.5%)<br>75 (2.3%)<br>75 (2.3%)<br>72 (2.2%)<br>72 (2.2%)<br>70 (2.2%)<br>69 (2.2%)<br>69 (2.2%)<br>68 (2.1%)<br>67 (2.1%)<br>292 (9.1%)		3200<br>(100%)	0<br>(0%)
16	sender_category<br>[character]	1. discrimination<br>2. insult<br>3. vilification<br>4. violence	615 (19.2%)<br>978 (30.6%)<br>625 (19.5%)<br>982 (30.7%)		3200<br>(100%)	0<br>(0%)
17	sender_hatescore<br>[numeric]	mean (sd) : 1.51 (0.5)<br>min < med < max :<br>1 < 2 < 2<br>IQR (CV) : 1 (0.33)	1 : 1568 (49.0%)<br>2 : 1632 (51.0%)		3200<br>(100%)	0<br>(0%)
18	target_reply<br>[character]	1. [target] hat [sender]s Account<br>2. [target] hat [sender]s Nachric<br>3. Bitte bleibe höflich. Wir habe<br>4. Bitte höre auf, andere anzugre<br>5. Du bist so blöd!<br>6. Halt's Maul Arschloch!<br>7. none	401 (12.5%)<br>399 (12.5%)<br>404 (12.6%)<br>396 (12.4%)<br>412 (12.9%)<br>388 (12.1%)<br>800 (25.0%)		3200<br>(100%)	0<br>(0%)
19	target_reply_category<br>[character]	1. appealing_to_norms<br>2. counter_aggression<br>3. none<br>4. platform_action	800 (25.0%)<br>800 (25.0%)<br>800 (25.0%)<br>800 (25.0%)		3200<br>(100%)	0<br>(0%)

Figure S6: Balance of vignette attributes in gross sample (German survey)

No	Variable	Stats / Values	Freqs (% of Valid)	Graph	Valid	Missing
1	id<br>[integer]	mean (sd) : 18803.83 (12022.21)<br>min < med < max :<br>13 < 18443 < 40968<br>IQR (CV) : 20457.5 (0.64)	3031 distinct values	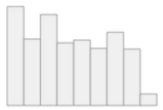	3200<br>(100%)	0<br>(0%)
2	topic<br>[character]	1. gender<br>2. ideologydems<br>3. ideologyreps<br>4. muslim	800 (25.0%)<br>800 (25.0%)<br>800 (25.0%)<br>800 (25.0%)	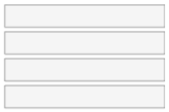	3200<br>(100%)	0<br>(0%)
3	gender_target<br>[character]	1. female<br>2. male<br>3. unknown	1195 (37.3%)<br>1205 (37.7%)<br>800 (25.0%)	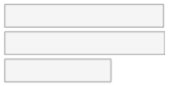	3200<br>(100%)	0<br>(0%)
4	religion_target<br>[character]	1. muslim<br>2. nonmuslim<br>3. unknown	1213 (37.9%)<br>1187 (37.1%)<br>800 (25.0%)	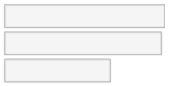	3200<br>(100%)	0<br>(0%)
5	ideology_target<br>[character]	1. conservative<br>2. liberal<br>3. unknown	402 (12.6%)<br>398 (12.4%)<br>2400 (75.0%)	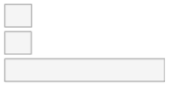	3200<br>(100%)	0<br>(0%)
6	name_target<br>[character]	1. Amir Rahman<br>2. Anna Schneider<br>3. Carolin Weber<br>4. Fatima Abad<br>5. Florian Schulz<br>6. Laura Fischer<br>7. Leyla Karimi<br>8. Lisa Meier<br>9. Lukas Becker<br>10. Mark Schmidt<br>11. Mohammed Nazir<br>12. Nadeem Shakir<br>13. Paul Wagner<br>14. Rashid Farra<br>15. Saba Malek<br>16. Team Deutschland<br>17. Team Global<br>18. Zeynep Omer	149 (4.7%)<br>118 (3.7%)<br>153 (4.8%)<br>162 (5.1%)<br>163 (5.1%)<br>137 (4.3%)<br>154 (4.8%)<br>153 (4.8%)<br>166 (5.2%)<br>151 (4.7%)<br>153 (4.8%)<br>154 (4.8%)<br>146 (4.6%)<br>123 (3.8%)<br>144 (4.5%)<br>402 (12.6%)<br>398 (12.4%)<br>174 (5.4%)	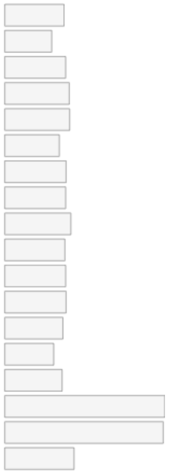	3200<br>(100%)	0<br>(0%)
7	gender_sender<br>[character]	1. female<br>2. male<br>3. unknown	1122 (35.1%)<br>1278 (39.9%)<br>800 (25.0%)	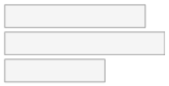	3200<br>(100%)	0<br>(0%)
8	religion_sender<br>[character]	1. muslim<br>2. nonmuslim<br>3. unknown	1121 (35.0%)<br>1279 (40.0%)<br>800 (25.0%)	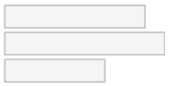	3200<br>(100%)	0<br>(0%)
9	ideology_sender<br>[character]	1. conservative<br>2. liberal<br>3. unknown	378 (11.8%)<br>422 (13.2%)<br>2400 (75.0%)	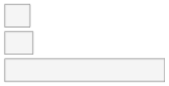	3200<br>(100%)	0<br>(0%)

Figure S7: Balance of vignette attributes in gross sample (German survey), *continued*

No	Variable	Stats / Values	Freqs (% of Valid)	Graph	Valid	Missing
10	name_sender<br>[character]	1. Amir Rahman<br>2. Anna Schneider<br>3. Carolin Weber<br>4. Fatima Abad<br>5. Florian Schulz<br>6. Laura Fischer<br>7. Leyla Karimi<br>8. Lisa Meier<br>9. Lukas Becker<br>10. Mark Schmidt<br>11. Mohammed Nazir<br>12. Nadeem Shakir<br>13. Paul Wagner<br>14. Rashid Farra<br>15. Saba Malek<br>16. Team Deutschland<br>17. Team Global<br>18. Zeynep Omer	162 (5.1%)<br>174 (5.4%)<br>168 (5.2%)<br>113 (3.5%)<br>167 (5.2%)<br>163 (5.1%)<br>117 (3.7%)<br>144 (4.5%)<br>153 (4.8%)<br>156 (4.9%)<br>165 (5.2%)<br>155 (4.8%)<br>154 (4.8%)<br>166 (5.2%)<br>117 (3.7%)<br>378 (11.8%)<br>422 (13.2%)<br>126 (3.9%)		3200<br>(100%)	0<br>(0%)
11	target_group<br>[character]	1. Alle AfDler<br>2. Alle Frauen<br>3. Alle Grünen<br>4. Alle muslimischen Einwanderer<br>5. Die meisten AfDler<br>6. Die meisten Frauen<br>7. Die meisten Grünen<br>8. Die meisten muslimischen Einwa<br>9. Du<br>10. Extrem feministische Frauen<br>11. Extremistische muslimische Ein<br>12. Linksextreme Grüne<br>13. Rechtsextreme AfDler	210 (6.6%)<br>218 (6.8%)<br>225 (7.0%)<br>217 (6.8%)<br>232 (7.2%)<br>211 (6.6%)<br>199 (6.2%)<br>206 (6.4%)<br>597 (18.7%)<br>200 (6.2%)<br>224 (7.0%)<br>227 (7.1%)<br>234 (7.3%)		3200<br>(100%)	0<br>(0%)
12	target_group_category<br>[character]	1. all<br>2. extremist<br>3. most<br>4. you	870 (27.2%)<br>885 (27.7%)<br>848 (26.5%)<br>597 (18.7%)		3200<br>(100%)	0<br>(0%)
13	target_message<br>[character]	1. Ich bin ein stolzer Feminist!<br>2. Ich bin ein stolzer muslimisch<br>3. Ich bin ein stolzes Mitglied d<br>4. Ich bin ein stolzes Mitglied d<br>5. Ich bin eine stolze Feministin<br>6. Ich bin eine stolze muslimisch<br>7. Ich unterstütze die Alternativ<br>8. Ich unterstütze die Grünen.<br>9. Ich unterstütze Feminismus.<br>10. Ich unterstütze muslimische Ei	249 (7.8%)<br>226 (7.1%)<br>422 (13.2%)<br>404 (12.6%)<br>145 (4.5%)<br>154 (4.8%)<br>378 (11.8%)<br>396 (12.4%)<br>406 (12.7%)<br>420 (13.1%)		3200<br>(100%)	0<br>(0%)
14	target_message_category<br>[character]	1. proud<br>2. support	1600 (50.0%)<br>1600 (50.0%)		3200<br>(100%)	0<br>(0%)

Figure S8: Balance of vignette attributes in gross sample (German survey), *continued*

No	Variable	Stats / Values	Freqs (% of Valid)	Graph	Valid	Missing
15	sender_message<br>[character]	1. sollten abgeknallt werden.<br>2. sind dumme Kreaturen.<br>3. sollten eine reingehauen bekom<br>4. sind Idioten.<br>5. sollten nicht wählen dürfen.<br>6. solltest eine reingehauen beko<br>7. bist eine dumme Kreatur.<br>8. bist ein Idiot.<br>9. solltest abgeknallt werden.<br>10. sind einfach nicht so schlau w<br>11. sollten abgeschoben werden.<br>12. sind AfDlern genetisch unterle<br>13. sind uns genetisch unterlegen.<br>14. sind einfach anders als wir.<br>15. sollten nicht in der Bundesweh<br>16. sind genetisch Männern unterle<br>17. sind einfach nicht so schlau w<br>18. sind Grünen genetisch unterleg<br>19. sollten davon abgehalten werde<br>20. sollten daran gehindert werde<br>[18 others]	380 (11.9%)<br>372 (11.6%)<br>362 (11.3%)<br>360 (11.2%)<br>149 (4.7%)<br>129 (4.0%)<br>126 (3.9%)<br>120 (3.8%)<br>111 (3.5%)<br>81 (2.5%)<br>81 (2.5%)<br>75 (2.3%)<br>75 (2.3%)<br>72 (2.2%)<br>72 (2.2%)<br>70 (2.2%)<br>69 (2.2%)<br>69 (2.2%)<br>68 (2.1%)<br>67 (2.1%)<br>292 (9.1%)		3200<br>(100%)	0<br>(0%)
16	sender_category<br>[character]	1. discrimination<br>2. insult<br>3. vilification<br>4. violence	615 (19.2%)<br>978 (30.6%)<br>625 (19.5%)<br>982 (30.7%)		3200<br>(100%)	0<br>(0%)
17	sender_hatescore<br>[numeric]	mean (sd) : 1.51 (0.5)<br>min < med < max :<br>1 < 2 < 2<br>IQR (CV) : 1 (0.33)	1 : 1568 (49.0%)<br>2 : 1632 (51.0%)		3200<br>(100%)	0<br>(0%)
18	target_reply<br>[character]	1. [target] hat [sender]s Account<br>2. [target] hat [sender]s Nachric<br>3. Bitte bleibe höflich. Wir habe<br>4. Bitte höre auf, andere anzugre<br>5. Du bist so blöd!<br>6. Halt's Maul Arschloch!<br>7. none	401 (12.5%)<br>399 (12.5%)<br>404 (12.6%)<br>396 (12.4%)<br>412 (12.9%)<br>388 (12.1%)<br>800 (25.0%)		3200<br>(100%)	0<br>(0%)
19	target_reply_category<br>[character]	1. appealing_to_norms<br>2. counter_aggression<br>3. none<br>4. platform_action	800 (25.0%)<br>800 (25.0%)<br>800 (25.0%)<br>800 (25.0%)		3200<br>(100%)	0<br>(0%)

C Additional descriptive evidence

Message	Hatefulness			Deletion rate	Distribution of hatefulness ratings
	Mean	SD	4+ rating		
Women should be killed.	4.38	0.89	87%	43%	
Democrats should be killed.	4.25	1.04	82%	50%	
Republicans should be killed.	4.24	1.03	80%	45%	
Muslim immigrants should be killed.	4.21	1.11	79%	46%	
Women should be punched in the face.	3.73	1.08	60%	43%	
Muslim immigrants should be punched in the face.	3.66	1.10	58%	46%	
Muslim immigrants are stupid animals.	3.49	1.12	50%	49%	
Muslim immigrants are genetically inferior to us.	3.44	1.15	53%	37%	
Women are stupid animals.	3.44	1.12	48%	44%	
Democrats should be punched in the face.	3.33	1.06	41%	42%	
Republicans should be punched in the face.	3.29	1.07	39%	44%	
Women are genetically inferior to men.	3.04	1.14	33%	33%	
Republicans are stupid animals.	2.99	1.10	29%	37%	
Democrats are stupid animals.	2.97	1.10	29%	34%	
Muslim immigrants should be deported.	2.93	1.17	32%	27%	
Democrats are genetically inferior to Republicans.	2.91	1.15	27%	34%	
Muslim immigrants should be banned from coming into our country.	2.91	1.16	30%	27%	
Muslim immigrants are dumb.	2.89	1.09	25%	35%	
Women are dumb.	2.87	1.13	27%	31%	
Republicans are genetically inferior to Democrats.	2.85	1.11	26%	30%	
Republicans should be not allowed to vote.	2.56	1.16	19%	24%	
Democrats should be not allowed to vote.	2.54	1.21	17%	22%	
Women should not be allowed to serve in the army.	2.49	1.19	19%	23%	
Republicans are dumb.	2.46	1.03	13%	25%	
Women should be caring mothers and not pursue a selfish career.	2.34	1.11	11%	22%	
Democrats are dumb.	2.29	0.93	8%	18%	
Democrats are just not as clever as Republicans.	2.10	0.98	7%	16%	
Republicans should be stopped from spreading falsehoods.	2.09	1.07	8%	18%	
Republicans are just not as clever as Democrats.	2.09	0.97	5%	17%	
Democrats should be stopped from spreading falsehoods.	1.96	0.97	6%	16%	
Muslim immigrants are just different from us.	1.78	1.00	5%	11%	
Women are just different from men.	1.65	0.87	2%	11%	

Figure S9: Hatefulness rankings of messages, U.S. sample. Addressing scope was reduced to the general target group (women, Muslim immigrants, Republicans, Democrats). The raw perceived hatefulness scale ranges from 1 = "Not hateful at all" to 5 = "Extremely hateful". For each message the table reports mean and SD of the hatefulness ratings, the share of ratings that were 4 "very hateful" or 5 "extremely hateful", and the share of decisions to opt for a deletion of the post containing the message.

Message	Hatefulness			Deletion rate	Distribution of hatefulness ratings
	Mean	SD	4+ rating		
Women should be killed.	4.42	1.00	83%	54%	
Muslim immigrants should be killed.	4.31	1.20	80%	56%	
Green voters should be killed.	4.17	1.17	74%	52%	
AfD voters should be killed.	4.15	1.16	73%	53%	
Women should be punched in the face.	3.71	1.17	59%	52%	
Muslim immigrants should be punched in the face.	3.66	1.15	56%	53%	
AfD voters should be punched in the face.	3.33	1.13	42%	47%	
Green voters should be punched in the face.	3.30	1.12	43%	45%	
Women are stupid animals.	3.19	1.06	38%	53%	
Muslim immigrants are stupid animals.	3.18	1.16	38%	48%	
Muslim immigrants are genetically inferior to us.	3.14	1.15	39%	54%	
Women are dumb.	2.92	1.15	29%	43%	
Muslim immigrants are dumb.	2.86	1.14	25%	44%	
Green voters are genetically inferior to AfD voters.	2.84	1.20	28%	43%	
AfD voters are stupid animals.	2.81	1.13	26%	42%	
Green voters are stupid animals.	2.77	1.07	23%	47%	
Women are genetically inferior to men.	2.68	1.26	23%	35%	
AfD voters are dumb.	2.62	1.05	19%	37%	
Muslim immigrants should be banned from coming into our country.	2.59	1.23	21%	27%	
Muslim immigrants should be deported.	2.59	1.24	22%	30%	
AfD voters are genetically inferior to Greeen voters.	2.47	1.19	19%	35%	
Green voters are dumb.	2.42	1.06	12%	40%	
AfD voters should be not allowed to vote.	2.30	1.09	13%	26%	
Green voters should be not allowed to vote.	2.29	1.19	15%	25%	
Green voters should be stopped from spreading falsehoods.	2.09	1.09	7%	18%	
Women should not be allowed to serve in the army.	2.07	1.18	8%	17%	
Green voters are just not as clever as AfD voters.	2.03	1.13	9%	18%	
AfD voters are just not as clever as Greeen voters.	2.01	1.05	8%	22%	
AfD voters should be stopped from spreading falsehoods.	1.92	0.99	8%	12%	
Women should be caring mothers and not pursue a selfish career.	1.90	1.03	4%	18%	
Muslim immigrants are just different from us.	1.66	0.93	4%	12%	
Women are just different from men.	1.64	1.05	5%	12%	

Figure S10: Hatefulness rankings of messages, German sample. Addressing scope was reduced to the general target group (women, Muslim immigrants, AfD voters, Green voters). The raw perceived hatefulness scale ranges from 1 = ”Not hateful at all” to 5 = ”Extremely hateful”. For each message the table reports mean and SD of the hatefulness ratings, the share of ratings that were 4 ”very hateful” or 5 ”extremely hateful”, and the share of decisions to opt for a deletion of the post containing the message.

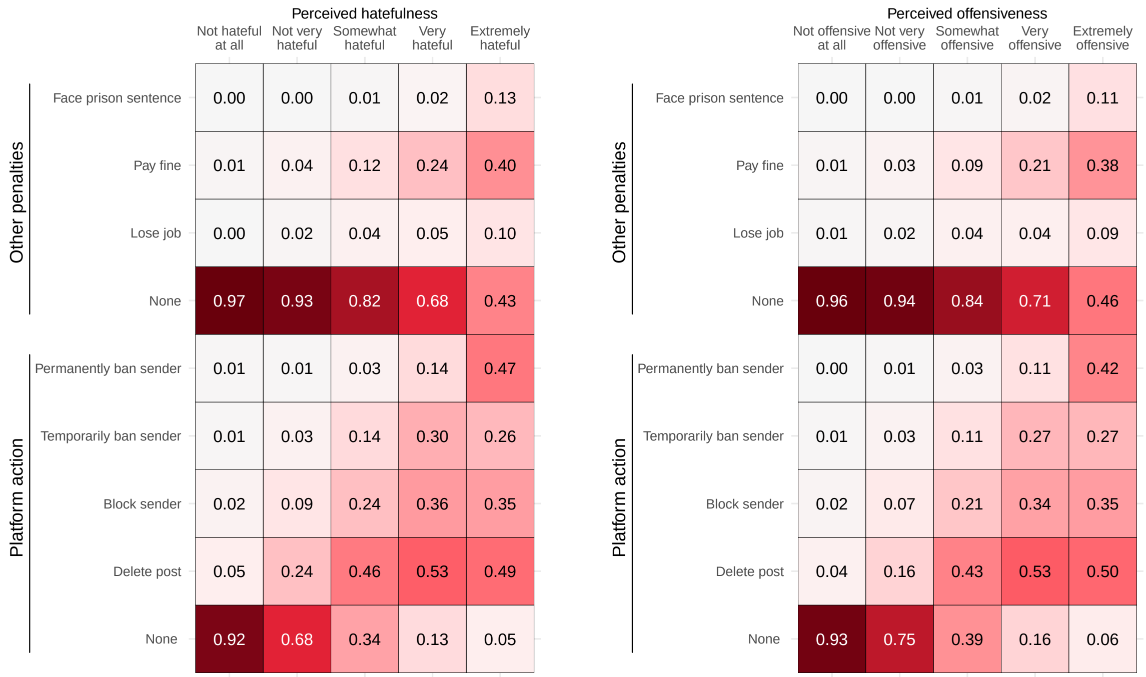

(a) Preferred action by perceived hatefulness

(b) Preferred action by perceived offensiveness

Figure S11: Relationship between perceptions of hate speech and preferences for sanctions for $n = 20,976$ hate speech vignettes (pooled sample). Cell values reflect share of selected action by perception score (e.g., for 97% of all posts that were perceived as “Not hateful at all”, respondents opted for “No further penalties”). Perceived hatefulness and offensiveness are measured on 5-point scales, preferences for platform action and further penalties are binary measures.

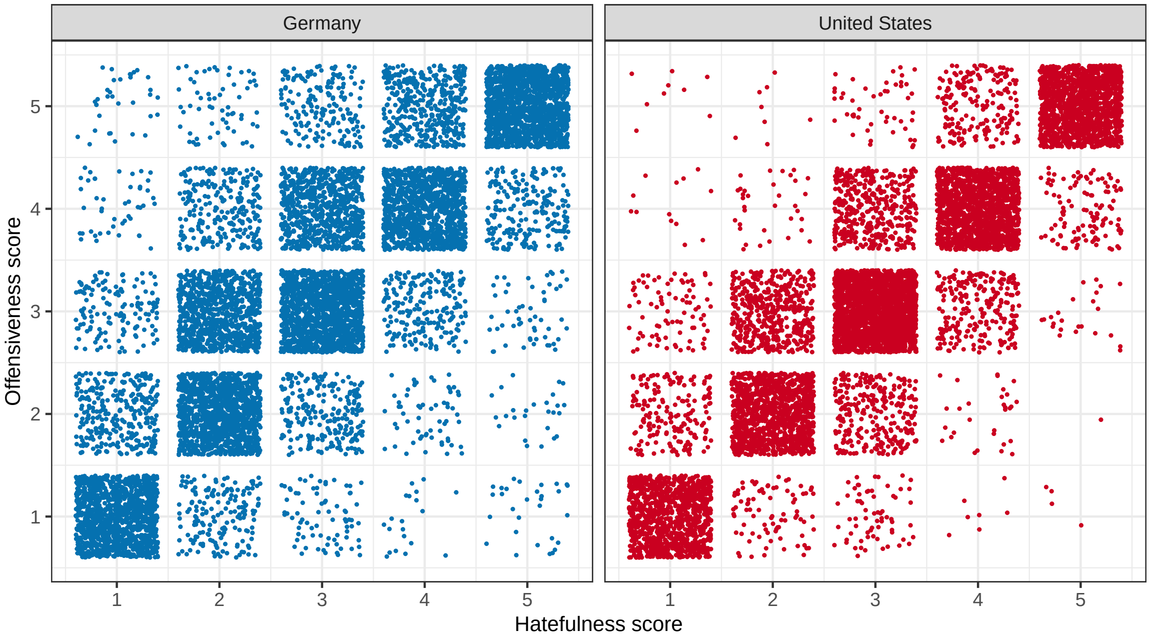

Figure S12: Hatefulness vs. offensiveness scores for $n = 20,976$ hate speech vignettes, by sample. Both scales range from 1 = "Not hateful/offensive at all" to 5 = "Extremely hateful/offensive". Pearson correlation between both measures is $r^{GER}_{hate.offense} = 0.79$ in the German sample and $r^{USA}_{hate.offense} = 0.89$ in the U.S. sample.

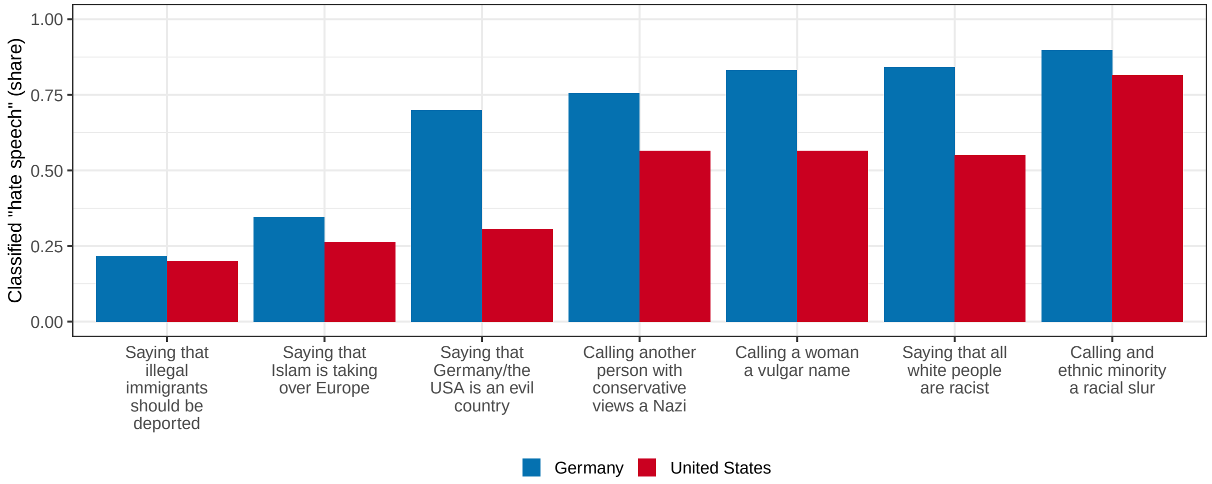

Figure S13: Hate speech classifications of various speech acts, by sample ($n^{\mathbf{GER}}$ = 1390, $n^{\mathbf{USA}}$ = 1232). Question wording: ”Which of the following would you label as hate speech?” ”Don’t know” answers excluded. As can be seen from the figure, the rankings of speech acts are similar across samples. Statements are generally perceived more hateful in the German than in the US sample. Statements not addressing a particular person (”Saying that X” vs. ”Calling someone X”) are classified as ”No hate speech” more often. One exception is ”Saying that all white people are racist”. This is probably driven by the fact that both samples are predominantly composed of white people, and respondents could project statements onto themselves.

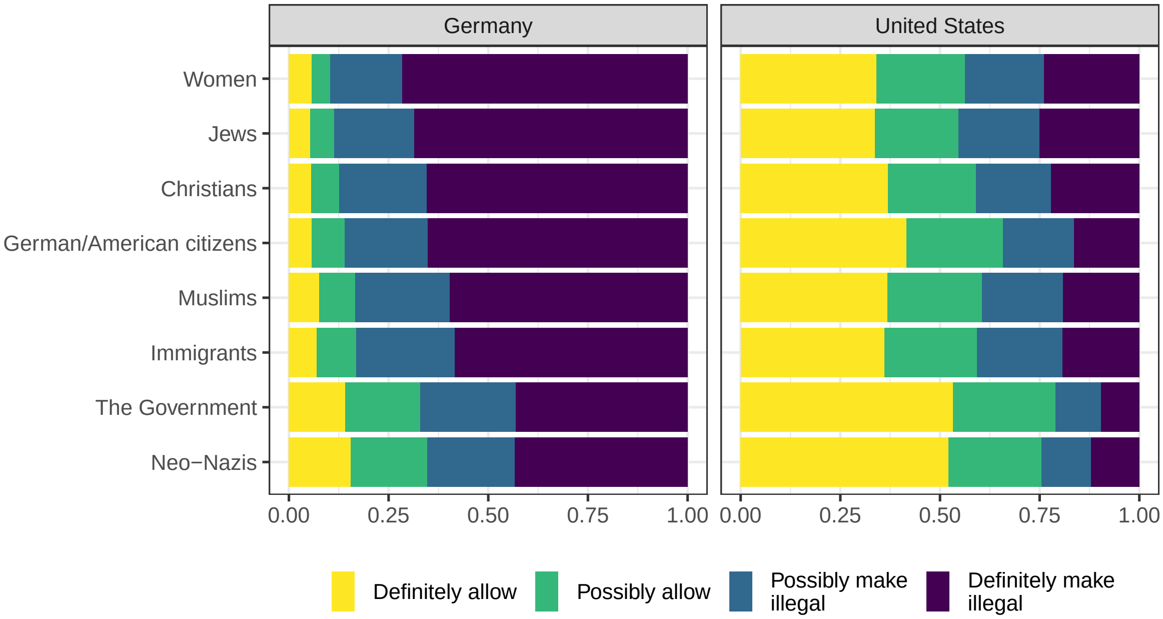

Figure S14: Support for hate speech regulation protecting specific groups, by sample ($n^{\mathbf{GER}} = 1390$, $n^{\mathbf{USA}} = 1232$). Question wording: "Would you support or oppose a law that would make it illegal to make insulting or hateful statements about [group]?" "Don't know" answers excluded. As can be seen from the figure, there is substantively more support for group-specific protection against hate speech in Germany than in the United States. The government and Neo-Nazis receive least sympathy for protection in both countries; women and Jews are perceived as being most worthy of protection.

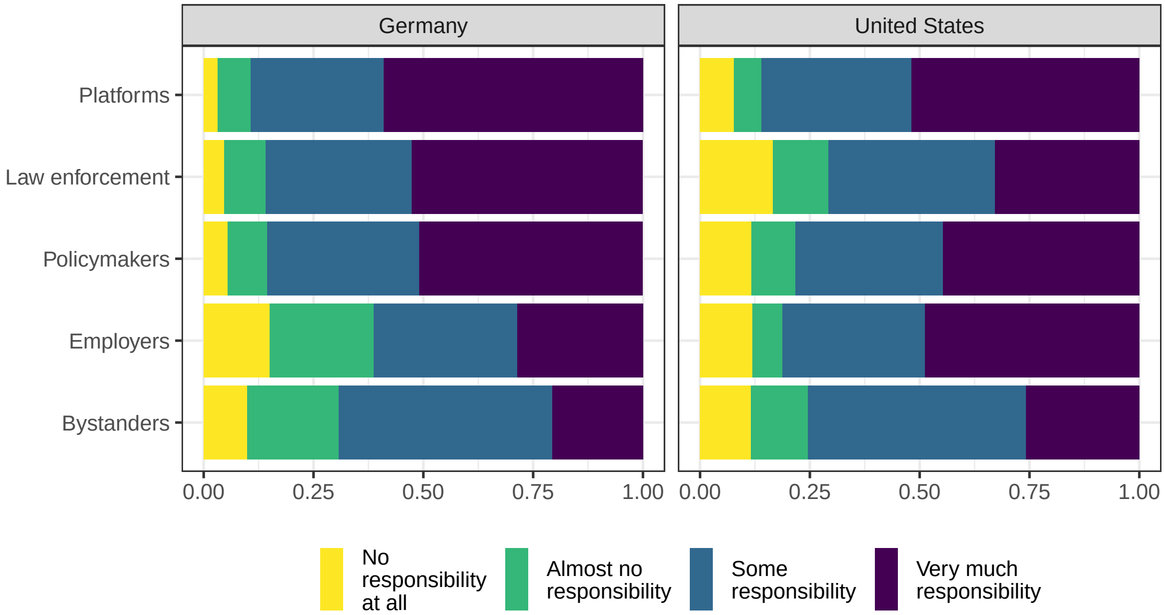

Figure S15: Attributed responsibility to take action against hate speech, by sample ($n^{\textbf{GER}} = 1390$, $n^{\textbf{USA}} = 1232$). Question wording: "To what extent, if at all, do you think each of the following groups should take responsibility in taking steps against online hate speech?" "Don't know" answers excluded. As can be seen from the figure, respondents in both samples attribute most responsibility to platforms. There is some variation across contexts: Relatively more responsibility is attributed to law enforcement in Germany and to employers in the United States. Bystanders are attributed little responsibility in both settings.

Table S11: Descriptive statistics of respondent characteristics, by country

		Germany		United States	
		N	Percent	N	Percent
Gender	Male	769	55	574	47
	Female	621	45	658	53
Age	18-29	126	9	59	5
	30-49	513	37	259	21
	50-69	622	45	557	45
	70+	87	6	257	21
Education	Low	221	16	219	18
	Intermediate	584	42	441	36
	High	581	42	572	46
Political interest	Low	164	12	158	13
	Intermediate	419	30	143	12
	High	765	55	826	67
Social media user	No	335	24	118	10
	Yes	1055	76	1011	82
Ideology	Left	387	28	444	36
	Center	645	46	368	30
	Right	214	15	391	32
Hate experience	No	1233	89	902	73
	Yes	115	8	230	19
Hate witness	No	1084	78	586	48
	Yes	264	19	546	44
Talk politics freely	No	386	28	695	56
	Yes	945	68	476	39
Party ID	CDU/CSU	217	16	0	0
	SPD	175	13	0	0
	FDP	107	8	0	0
	Greens	221	16	0	0
	Left	187	13	0	0
	AfD	196	14	0	0
	Others	50	4	0	0
	DK	0	0	0	0
Party ID	Democrat	0	0	487	40
	Republican	0	0	315	26
	Independent	0	0	357	29

D Additional results

D.1 Statistical power of vignette design

To calculate the power of the vignette experiment, the effective sample size is given by the number of respondents multiplied by the number of tasks, i.e., the number of vignettes per respondent. In our vignette experiment, each respondent received 8 vignettes, yielding effective sample sizes of 9,856 for the U.S. sample, 11,120 for the German sample, and 20,976 for the pooled sample.

To determine the power for a given AMCE, we rely on the formula provided by Schuessler & Freitag (*1*). In this context, the number of vignette *attributes* does not affect the power of a given AMCE, but the number of attribute *levels* does. The maximum number of levels in our vignette experiment is 8 (i.e., the message content), and we use this upper benchmark for our power calculation. Figure S16 in the Supplemental Material plots the size of the AMCEs against the formally calculated power separately for the U.S., German, and pooled samples. This formal power analysis suggests that, given our sample size, we are able to detect AMCEs as small as 0.05 with conventional power of 0.8 and an alpha of 0.05. Power for attributes with fewer levels is even higher. Thus, *a priori*, there is no reason to assume that we are underpowered.

Detecting interactions between attribute levels and respondent characteristics (AMCIEs) is more challenging. Using 8 attribute levels as a benchmark and assessing their interaction with respondents' gender in the pooled sample, we are only able to detect effect differences of 0.08 with power of 0.8 and an alpha of 0.05. However, we would not be able to detect effect differences between intersectional minorities, e.g., Black women (roughly 7 percent of the U.S. population), and all other demographics. For Black women in the U.S., the power for detecting an AMCIE of this size (0.08) drops to 0.177. Conversely, we would only detect a very large effect difference of 0.2 with power of 0.8 and an alpha of 0.05.

Experiment 2, i.e., the framing experiment, was embedded in the vignette experiment, so no additional power analysis is required. Experiment 3, i.e., the exposure experiment, follows a simple 1x2 between-subject design. A formal power analysis (for a two-sided t-test or, equivalently, bivariate regression) is therefore straightforward. Given conventional power of 0.8 and an alpha of 0.05, we are able to detect small effects of Cohen's $d = 0.11$ in the pooled sample (which corresponds to a difference of 4 percentage points), Cohen's $d = 0.16$ in the U.S. sample (which corresponds to a difference of 6 percentage points), and Cohen's $d = 0.15$ in the German

Figure S16: Statistical power of detecting a given AMCE for an vignette attribute with 8 levels.

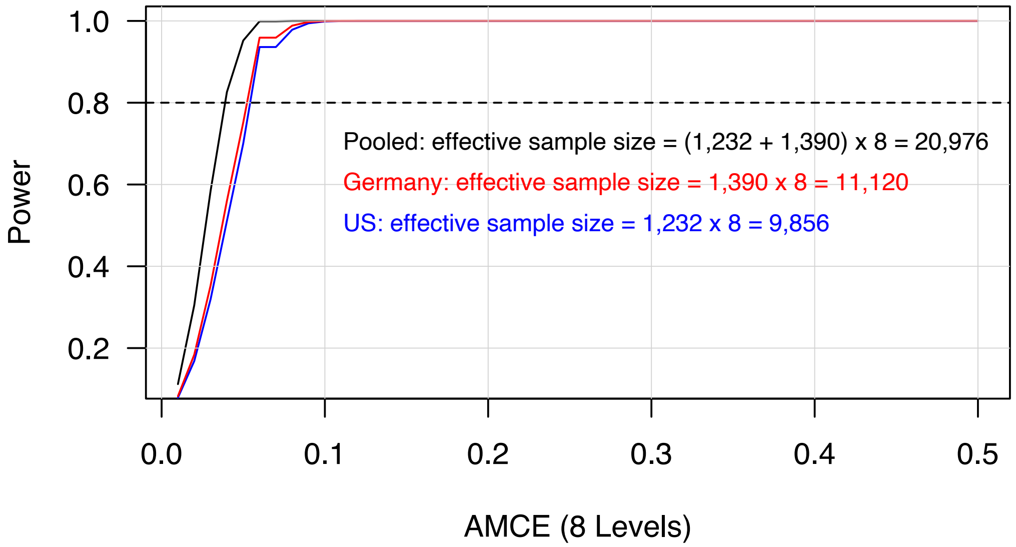

sample (which corresponds to a difference of 5 percentage points). Again, there is little reason to assume that we are underpowered when it comes to the main effects of the exposure experiment.

D.2 Vignette evaluations, mixed-effects models

	Offensive	Hateful	No action	Delete	Block	Temp. ban	Perm. ban	No penalties	Lose job	Fine	Priso
Type (severity): Discrimination (extreme)	0.05*	0.05*	−0.07*	0.04*	0.02*	0.02*	0.02	−0.01	0.00	0.01	0.00
	[0.04; 0.06]	[0.04; 0.06]	[−0.09; −0.04]	[0.02; 0.07]	[0.00; 0.04]	[0.00; 0.04]	[−0.00; 0.03]	[−0.03; 0.01]	[−0.01; 0.01]	[−0.01; 0.02]	[−0.01; [illegible]
Type (severity): Vilification (moderate)	−0.06*	−0.07*	0.08*	−0.05*	−0.05*	−0.02*	−0.00	0.02*	−0.00	−0.02*	0.00
	[−0.07; −0.05]	[−0.08; −0.06]	[0.06; 0.11]	[−0.08; −0.03]	[−0.07; −0.03]	[−0.04; −0.00]	[−0.02; 0.01]	[0.00; 0.04]	[−0.02; 0.01]	[−0.04; −0.00]	[−0.01; [illegible]
Type (severity): Vilification (extreme)	0.19*	0.14*	−0.25*	0.18*	0.10*	0.08*	0.06*	−0.09*	0.01*	0.07*	0.01*
	[0.17; 0.20]	[0.13; 0.15]	[−0.28; −0.23]	[0.15; 0.20]	[0.08; 0.12]	[0.06; 0.10]	[0.04; 0.07]	[−0.11; −0.07]	[0.00; 0.03]	[0.05; 0.08]	[0.00; [illegible]
Type (severity): Insult (moderate)	0.14*	0.09*	−0.19*	0.15*	0.05*	0.05*	0.03*	−0.05*	0.00	0.05*	0.01
	[0.13; 0.15]	[0.08; 0.10]	[−0.21; −0.17]	[0.12; 0.17]	[0.03; 0.07]	[0.03; 0.07]	[0.01; 0.04]	[−0.07; −0.03]	[−0.01; 0.01]	[0.03; 0.07]	[−0.00; [illegible]
Type (severity): Insult (extreme)	0.23*	0.18*	−0.34*	0.24*	0.13*	0.10*	0.07*	−0.09*	0.01*	0.08*	0.01*
	[0.21; 0.24]	[0.17; 0.19]	[−0.36; −0.32]	[0.22; 0.26]	[0.11; 0.14]	[0.09; 0.12]	[0.06; 0.08]	[−0.11; −0.07]	[0.00; 0.02]	[0.06; 0.10]	[0.00; [illegible]
Type (severity): Violence (moderate)	0.24*	0.26*	−0.42*	0.27*	0.16*	0.14*	0.11*	−0.16*	0.02*	0.14*	0.02*
	[0.23; 0.25]	[0.25; 0.27]	[−0.44; −0.40]	[0.24; 0.29]	[0.15; 0.18]	[0.12; 0.16]	[0.09; 0.12]	[−0.18; −0.14]	[0.01; 0.03]	[0.12; 0.15]	[0.01; [illegible]
Type (severity): Violence (extreme)	0.38*	0.42*	−0.55*	0.30*	0.20*	0.18*	0.32*	−0.37*	0.05*	0.28*	0.09*
	[0.37; 0.39]	[0.40; 0.43]	[−0.57; −0.53]	[0.28; 0.32]	[0.18; 0.22]	[0.16; 0.20]	[0.31; 0.34]	[−0.38; −0.35]	[0.04; 0.06]	[0.26; 0.30]	[0.08; [illegible]
Topic: Liberals	0.01*	−0.00	−0.01	0.00	0.01	−0.00	0.01	−0.02*	0.00	0.02*	−0.00
	[0.00; 0.02]	[−0.01; 0.01]	[−0.02; 0.01]	[−0.01; 0.02]	[−0.00; 0.03]	[−0.01; 0.01]	[−0.00; 0.02]	[−0.03; −0.01]	[−0.01; 0.01]	[0.01; 0.03]	[−0.01; [illegible]
Topic: Muslim immigrants	0.06*	0.07*	−0.09*	0.05*	0.05*	0.03*	0.05*	−0.05*	0.01*	0.05*	0.01*
	[0.05; 0.07]	[0.06; 0.08]	[−0.11; −0.08]	[0.04; 0.07]	[0.04; 0.07]	[0.01; 0.04]	[0.04; 0.06]	[−0.07; −0.04]	[0.00; 0.02]	[0.03; 0.06]	[0.00; [illegible]
Topic: Women	0.06*	0.04*	−0.05*	0.02	0.05*	0.02*	0.05*	−0.05*	0.01*	0.04*	0.01*
	[0.05; 0.07]	[0.03; 0.04]	[−0.07; −0.04]	[−0.00; 0.03]	[0.03; 0.06]	[0.00; 0.03]	[0.03; 0.06]	[−0.07; −0.04]	[0.00; 0.02]	[0.03; 0.06]	[0.00; [illegible]
Addressing scope: Extremist	−0.00	0.01*	0.01	−0.00	−0.02*	−0.01	0.00	−0.01	0.00	0.00	−0.00
	[−0.01; 0.00]	[0.00; 0.02]	[−0.01; 0.03]	[−0.02; 0.01]	[−0.03; −0.01]	[−0.02; 0.01]	[−0.01; 0.01]	[−0.02; 0.01]	[−0.00; 0.01]	[−0.01; 0.02]	[−0.01; [illegible]
Addressing scope: Most	0.02*	0.02*	−0.00	0.00	−0.02*	−0.00	0.01	−0.01	0.01	0.01	0.00
	[0.01; 0.02]	[0.01; 0.02]	[−0.02; 0.01]	[−0.01; 0.02]	[−0.04; −0.01]	[−0.02; 0.01]	[−0.00; 0.02]	[−0.02; 0.01]	[−0.00; 0.01]	[−0.01; 0.02]	[−0.01; [illegible]
Addressing scope: All	0.03*	0.03*	−0.02*	0.02*	−0.02*	−0.00	0.01*	−0.01	0.00	0.01	0.00
	[0.02; 0.03]	[0.02; 0.04]	[−0.04; −0.01]	[0.00; 0.03]	[−0.03; −0.00]	[−0.02; 0.01]	[0.00; 0.02]	[−0.02; 0.00]	[−0.00; 0.01]	[−0.00; 0.02]	[−0.00; [illegible]
Identity (sender): Anonymous | Liberal	0.00	−0.00	0.01	−0.01	−0.00	−0.01	0.01	−0.01	0.01	0.00	0.00
	[−0.01; 0.01]	[−0.01; 0.01]	[−0.02; 0.03]	[−0.03; 0.02]	[−0.02; 0.02]	[−0.03; 0.01]	[−0.01; 0.02]	[−0.03; 0.01]	[−0.00; 0.02]	[−0.02; 0.02]	[−0.01; [illegible]
Identity (sender): Female | Muslim	−0.00	0.00	0.00	−0.01	0.00	−0.01	−0.00	0.01	0.01	−0.01	−0.00
	[−0.01; 0.01]	[−0.01; 0.01]	[−0.02; 0.02]	[−0.03; 0.01]	[−0.02; 0.02]	[−0.03; 0.00]	[−0.01; 0.01]	[−0.01; 0.02]	[−0.00; 0.01]	[−0.03; 0.00]	[−0.01; [illegible]
Identity (sender): Female | Non-Muslim	−0.01	−0.01	0.02*	−0.01	−0.01	−0.00	−0.01	0.02	0.00	−0.02*	−0.01*
	[−0.01; 0.00]	[−0.02; 0.00]	[0.00; 0.04]	[−0.03; 0.01]	[−0.02; 0.01]	[−0.02; 0.01]	[−0.03; 0.00]	[−0.00; 0.03]	[−0.01; 0.01]	[−0.03; −0.00]	[−0.02; [illegible]
Identity (sender): Male | Muslim	0.00	0.01	−0.01	−0.01	0.00	−0.00	0.01	−0.00	0.00	−0.01	0.00
	[−0.01; 0.01]	[−0.00; 0.02]	[−0.03; 0.01]	[−0.03; 0.01]	[−0.02; 0.02]	[−0.02; 0.02]	[−0.01; 0.02]	[−0.02; 0.01]	[−0.00; 0.01]	[−0.03; 0.00]	[−0.01; [illegible]
Identity (sender): Male | Non-Muslim	−0.00	−0.01	0.01	0.01	−0.00	−0.00	−0.01*	0.01	0.01	−0.02*	−0.00
	[−0.01; 0.01]	[−0.02; 0.00]	[−0.01; 0.02]	[−0.01; 0.03]	[−0.02; 0.01]	[−0.02; 0.01]	[−0.03; −0.00]	[−0.01; 0.03]	[−0.00; 0.02]	[−0.03; −0.00]	[−0.01; [illegible]
Identity (target): Anonymous | Liberal	0.00	0.00	−0.00	0.00	0.00	0.01	0.01	−0.01	0.00	0.01	−0.00
	[−0.01; 0.01]	[−0.01; 0.01]	[−0.02; 0.02]	[−0.02; 0.03]	[−0.02; 0.02]	[−0.01; 0.03]	[−0.01; 0.02]	[−0.02; 0.01]	[−0.01; 0.01]	[−0.01; 0.03]	[−0.01; [illegible]
Identity (target): Female | Muslim	0.00	0.00	−0.01	0.01	0.00	−0.01	0.00	−0.00	0.00	−0.00	−0.00
	[−0.01; 0.01]	[−0.01; 0.01]	[−0.02; 0.01]	[−0.01; 0.03]	[−0.02; 0.02]	[−0.02; 0.01]	[−0.01; 0.01]	[−0.02; 0.01]	[−0.01; 0.01]	[−0.02; 0.01]	[−0.01; [illegible]
Identity (target): Female | Non-Muslim	0.00	−0.00	0.00	0.00	0.01	0.02*	−0.01	0.00	−0.00	0.01	−0.00
	[−0.01; 0.01]	[−0.01; 0.01]	[−0.02; 0.02]	[−0.01; 0.02]	[−0.01; 0.02]	[0.00; 0.03]	[−0.02; 0.01]	[−0.01; 0.02]	[−0.01; 0.01]	[−0.01; 0.02]	[−0.01; [illegible]
Identity (target): Male | Muslim	−0.00	−0.00	0.01	−0.00	−0.01	0.01	−0.01	−0.00	−0.00	0.01	−0.01
	[−0.01; 0.01]	[−0.01; 0.01]	[−0.01; 0.03]	[−0.02; 0.02]	[−0.02; 0.01]	[−0.00; 0.03]	[−0.02; 0.00]	[−0.02; 0.01]	[−0.01; 0.01]	[−0.01; 0.02]	[−0.01; [illegible]
Identity (target): Male | Non-Muslim	−0.01	−0.01	0.01	0.01	−0.00	0.00	−0.01	0.00	−0.00	−0.00	−0.01*
	[−0.01; 0.00]	[−0.02; 0.00]	[−0.01; 0.03]	[−0.01; 0.03]	[−0.02; 0.02]	[−0.01; 0.02]	[−0.02; 0.01]	[−0.01; 0.02]	[−0.01; 0.00]	[−0.02; 0.01]	[−0.01; [illegible]
Target message: Group support	−0.01*	−0.01*	0.01	−0.00	−0.01	0.00	−0.00	−0.00	−0.00	−0.00	0.00
	[−0.01; −0.00]	[−0.01; −0.00]	[−0.00; 0.02]	[−0.01; 0.01]	[−0.02; 0.00]	[−0.00; 0.01]	[−0.01; 0.01]	[−0.01; 0.00]	[−0.00; 0.00]	[−0.01; 0.01]	[−0.00; [illegible]
Reaction (target): Appealing to norms	−0.00	0.00	−0.01	0.00	0.01	0.00	−0.00	−0.00	−0.00	0.00	−0.00
	[−0.01; 0.01]	[−0.00; 0.01]	[−0.03; 0.00]	[−0.01; 0.02]	[−0.01; 0.02]	[−0.01; 0.01]	[−0.01; 0.01]	[−0.02; 0.01]	[−0.01; 0.01]	[−0.01; 0.01]	[−0.01; [illegible]
Reaction (target): Counter aggression	0.01*	0.01*	−0.03*	0.02*	0.01	−0.00	0.00	−0.01*	0.00	0.01	−0.00
	[0.00; 0.02]	[0.00; 0.02]	[−0.04; −0.01]	[0.01; 0.04]	[−0.01; 0.02]	[−0.01; 0.01]	[−0.01; 0.01]	[−0.02; −0.00]	[−0.01; 0.01]	[−0.00; 0.02]	[−0.01; [illegible]
Reaction (target): Platform action	0.00	0.01*	−0.04*	0.01	0.02*	0.00	0.01	−0.01	0.00	0.01	0.00
	[−0.01; 0.01]	[0.00; 0.01]	[−0.05; −0.02]	[−0.01; 0.02]	[0.01; 0.03]	[−0.01; 0.02]	[−0.00; 0.02]	[−0.02; 0.00]	[−0.00; 0.01]	[−0.00; 0.02]	[−0.01; [illegible]
Num. obs.	20760	20589	20976	20976	20976	20976	20976	20976	20976	20976	20976
Num. groups: personid	2617	2617	2622	2622	2622	2622	2622	2622	2622	2622	2622
Num. groups: deckid	768	768	768	768	768	768	768	768	768	768	768
Num. groups: country	2	2	2	2	2	2	2	2	2	2	2
Var: personid (Intercept)	0.02	0.02	0.08	0.07	0.06	0.03	0.03	0.07	0.01	0.04	0.01
Var: deckid (Intercept)	0.00	0.00	0.00	0.00	0.00	0.00	0.00	0.00	0.00	0.00	0.00
Var: country (Intercept)	0.00	0.00	0.00	0.00	0.00	0.00	0.00	0.01	0.00	0.01	0.00
Var: Residual	0.03	0.03	0.12	0.14	0.11	0.09	0.06	0.09	0.03	0.08	0.02

* Null hypothesis value outside the confidence interval.

Table S12: Average marginal component effects of content and context attributes on citizens' hate speech perceptions and preferences for action (pooled sample). Offensiveness and hatefulness scores were measured on five-point scales and rescaled to 0-1. Support for action was measured on a binary scale, 0-1. Linear mixed-effects models with person, vignette deck, and country random effects. 95% confidence intervals in parentheses.

	Offensive	Hateful	No action	Delete	Block	Temp. ban	Perm. ban	No penalties	Lose job	Fine	Prison
Type (severity): Discrimination (extreme)	0.04*	0.04*	−0.07*	0.06*	0.03*	0.02	0.01	0.01	−0.00	0.00	0.00
	[0.02; 0.05]	[0.02; 0.05]	[−0.10; −0.04]	[0.03; 0.10]	[0.00; 0.06]	[−0.01; 0.04]	[−0.01; 0.03]	[−0.02; 0.04]	[−0.02; 0.01]	[−0.03; 0.03]	[−0.01; 0.0
Type (severity): Vilification (moderate)	−0.03*	−0.06*	0.05*	−0.03	−0.03	−0.02	0.01	0.01	−0.01	−0.01	0.00
	[−0.05; −0.02]	[−0.08; −0.04]	[0.02; 0.08]	[−0.06; 0.01]	[−0.06; 0.00]	[−0.05; 0.01]	[−0.01; 0.03]	[−0.02; 0.04]	[−0.02; 0.01]	[−0.04; 0.02]	[−0.01; 0.0
Type (severity): Vilification (extreme)	0.22*	0.13*	−0.30*	0.23*	0.11*	0.08*	0.06*	−0.11*	0.00	0.10*	0.02*
	[0.20; 0.23]	[0.12; 0.15]	[−0.33; −0.27]	[0.20; 0.27]	[0.08; 0.14]	[0.05; 0.11]	[0.04; 0.09]	[−0.14; −0.08]	[−0.01; 0.02]	[0.07; 0.13]	[0.00; 0.0
Type (severity): Insult (moderate)	0.21*	0.12*	−0.29*	0.22*	0.09*	0.07*	0.04*	−0.08*	−0.01	0.09*	0.01
	[0.19; 0.23]	[0.10; 0.13]	[−0.32; −0.26]	[0.19; 0.25]	[0.06; 0.11]	[0.05; 0.10]	[0.02; 0.06]	[−0.10; −0.05]	[−0.02; 0.01]	[0.06; 0.11]	[−0.00; 0.0
Type (severity): Insult (extreme)	0.27*	0.17*	−0.39*	0.29*	0.13*	0.11*	0.08*	−0.12*	0.00	0.12*	0.01
	[0.25; 0.28]	[0.16; 0.19]	[−0.42; −0.36]	[0.26; 0.32]	[0.10; 0.16]	[0.08; 0.13]	[0.06; 0.10]	[−0.14; −0.09]	[−0.01; 0.01]	[0.09; 0.14]	[−0.00; 0.0
Type (severity): Violence (moderate)	0.27*	0.27*	−0.46*	0.30*	0.18*	0.15*	0.11*	−0.20*	0.00	0.19*	0.03*
	[0.25; 0.29]	[0.26; 0.29]	[−0.49; −0.43]	[0.27; 0.34]	[0.15; 0.20]	[0.12; 0.17]	[0.09; 0.14]	[−0.23; −0.18]	[−0.01; 0.02]	[0.17; 0.22]	[0.02; 0.0
Type (severity): Violence (extreme)	0.39*	0.43*	−0.57*	0.35*	0.20*	0.19*	0.31*	−0.44*	0.02*	0.37*	0.11*
	[0.37; 0.40]	[0.41; 0.44]	[−0.60; −0.54]	[0.32; 0.38]	[0.17; 0.22]	[0.17; 0.22]	[0.29; 0.33]	[−0.47; −0.42]	[0.01; 0.03]	[0.34; 0.40]	[0.09; 0.1
Topic: Liberals	0.02*	0.00	−0.02	0.01	0.02	−0.00	0.01	−0.02*	−0.00	0.02*	−0.00
	[0.01; 0.03]	[−0.01; 0.01]	[−0.04; 0.00]	[−0.01; 0.03]	[−0.00; 0.03]	[−0.02; 0.02]	[−0.00; 0.03]	[−0.04; −0.00]	[−0.01; 0.01]	[0.00; 0.04]	[−0.01; 0.0
Topic: Muslim immigrants	0.05*	0.07*	−0.09*	0.06*	0.04*	0.02	0.05*	−0.06*	−0.01	0.07*	0.01
	[0.04; 0.07]	[0.06; 0.08]	[−0.11; −0.07]	[0.04; 0.09]	[0.02; 0.06]	[−0.00; 0.04]	[0.03; 0.06]	[−0.08; −0.04]	[−0.01; 0.00]	[0.05; 0.08]	[−0.00; 0.0
Topic: Women	0.06*	0.03*	−0.04*	0.02	0.03*	0.01	0.05*	−0.07*	0.00	0.06*	0.02*
	[0.05; 0.07]	[0.02; 0.04]	[−0.06; −0.02]	[−0.01; 0.04]	[0.01; 0.05]	[−0.01; 0.03]	[0.04; 0.07]	[−0.09; −0.05]	[−0.01; 0.01]	[0.04; 0.08]	[0.01; 0.0
Addressing scope: Extremist	−0.01*	0.02*	0.03*	−0.01	−0.03*	−0.01	−0.00	−0.01	0.00	0.01	−0.00
	[−0.02; −0.00]	[0.00; 0.03]	[0.01; 0.05]	[−0.03; 0.01]	[−0.05; −0.01]	[−0.03; 0.01]	[−0.02; 0.01]	[−0.03; 0.01]	[−0.01; 0.01]	[−0.01; 0.03]	[−0.01; 0.0
Addressing scope: Most	0.01*	0.01	0.01	0.01	−0.02*	−0.01	−0.00	−0.01	0.00	0.01	0.00
	[0.00; 0.02]	[−0.00; 0.02]	[−0.02; 0.03]	[−0.02; 0.03]	[−0.04; −0.00]	[−0.03; 0.01]	[−0.02; 0.01]	[−0.03; 0.01]	[−0.01; 0.01]	[−0.01; 0.03]	[−0.01; 0.0
Addressing scope: All	0.02*	0.03*	−0.01	0.02	−0.02	−0.00	0.01	−0.01	0.00	0.01	0.00
	[0.01; 0.04]	[0.02; 0.04]	[−0.03; 0.01]	[−0.01; 0.04]	[−0.04; 0.00]	[−0.02; 0.01]	[−0.01; 0.02]	[−0.03; 0.01]	[−0.01; 0.01]	[−0.01; 0.03]	[−0.01; 0.0
Identity (sender): Anonymous | Liberal	−0.01	−0.01	0.02	−0.00	−0.01	−0.03	0.01	−0.01	0.01	−0.00	0.00
	[−0.02; 0.01]	[−0.02; 0.01]	[−0.01; 0.05]	[−0.04; 0.03]	[−0.04; 0.02]	[−0.05; 0.00]	[−0.01; 0.03]	[−0.04; 0.01]	[−0.00; 0.02]	[−0.03; 0.02]	[−0.01; 0.0
Identity (sender): Female | Muslim	−0.01	−0.00	0.01	−0.01	−0.01	−0.02	0.00	0.01	−0.00	−0.02	−0.00
	[−0.02; 0.01]	[−0.02; 0.01]	[−0.02; 0.04]	[−0.04; 0.03]	[−0.04; 0.01]	[−0.05; 0.00]	[−0.02; 0.02]	[−0.02; 0.03]	[−0.01; 0.01]	[−0.05; 0.00]	[−0.01; 0.0
Identity (sender): Female | Non-Muslim	−0.02*	−0.02*	0.05*	−0.02	−0.02	−0.01	−0.02*	0.03*	−0.00	−0.03*	−0.01
	[−0.04; −0.01]	[−0.03; −0.01]	[0.02; 0.07]	[−0.05; 0.01]	[−0.04; 0.01]	[−0.04; 0.01]	[−0.04; −0.00]	[0.00; 0.05]	[−0.02; 0.01]	[−0.06; −0.01]	[−0.02; 0.0
Identity (sender): Male | Muslim	−0.01	0.00	−0.00	−0.00	−0.01	−0.01	0.01	−0.00	0.01	−0.02	0.00
	[−0.02; 0.01]	[−0.01; 0.02]	[−0.03; 0.03]	[−0.03; 0.03]	[−0.03; 0.02]	[−0.04; 0.01]	[−0.01; 0.03]	[−0.03; 0.02]	[−0.00; 0.02]	[−0.04; 0.00]	[−0.01; 0.0
Identity (sender): Male | Non-Muslim	−0.01	−0.01	0.01	0.00	−0.02	−0.01	−0.01	0.01	0.01*	−0.03*	0.00
	[−0.02; 0.01]	[−0.02; 0.01]	[−0.01; 0.04]	[−0.03; 0.03]	[−0.04; 0.01]	[−0.03; 0.01]	[−0.02; 0.01]	[−0.01; 0.03]	[0.00; 0.02]	[−0.06; −0.01]	[−0.01; 0.0
Identity (target): Anonymous | Liberal	−0.00	−0.00	0.02	−0.00	−0.00	0.01	−0.00	−0.01	0.01	0.00	0.00
	[−0.02; 0.01]	[−0.02; 0.01]	[−0.01; 0.05]	[−0.03; 0.03]	[−0.03; 0.02]	[−0.02; 0.03]	[−0.02; 0.02]	[−0.04; 0.02]	[−0.00; 0.02]	[−0.02; 0.03]	[−0.01; 0.0
Identity (target): Female | Muslim	0.01	0.00	0.00	0.01	−0.00	−0.00	0.01	−0.01	0.01	−0.00	0.00
	[−0.01; 0.02]	[−0.01; 0.01]	[−0.02; 0.03]	[−0.02; 0.04]	[−0.03; 0.02]	[−0.03; 0.02]	[−0.01; 0.02]	[−0.03; 0.01]	[−0.00; 0.02]	[−0.03; 0.02]	[−0.01; 0.0
Identity (target): Female | Non-Muslim	0.00	−0.01	0.01	−0.01	0.01	0.03*	−0.01	0.01	0.01*	−0.00	−0.01
	[−0.01; 0.02]	[−0.02; 0.01]	[−0.01; 0.04]	[−0.04; 0.02]	[−0.01; 0.03]	[0.00; 0.05]	[−0.03; 0.01]	[−0.02; 0.03]	[0.00; 0.02]	[−0.02; 0.02]	[−0.02; 0.0
Identity (target): Male | Muslim	−0.01	−0.01	0.03*	−0.01	−0.01	0.02	−0.00	−0.01	0.01	0.00	0.00
	[−0.02; 0.01]	[−0.03; 0.00]	[0.00; 0.05]	[−0.04; 0.01]	[−0.03; 0.01]	[−0.01; 0.04]	[−0.02; 0.01]	[−0.03; 0.01]	[−0.00; 0.02]	[−0.02; 0.02]	[−0.01; 0.0
Identity (target): Male | Non-Muslim	−0.00	−0.01	0.01	−0.00	0.01	0.01	−0.01	−0.00	0.01	−0.00	−0.00
	[−0.01; 0.01]	[−0.02; 0.00]	[−0.01; 0.04]	[−0.03; 0.03]	[−0.02; 0.03]	[−0.01; 0.03]	[−0.03; 0.01]	[−0.03; 0.02]	[−0.00; 0.02]	[−0.03; 0.02]	[−0.02; 0.0
Target message: Group support	−0.01*	−0.01	0.01	−0.01	−0.01	0.00	−0.00	−0.01	0.01	−0.01	0.01
	[−0.02; −0.00]	[−0.01; 0.00]	[−0.01; 0.02]	[−0.02; 0.01]	[−0.02; 0.01]	[−0.01; 0.01]	[−0.01; 0.01]	[−0.02; 0.01]	[−0.00; 0.01]	[−0.02; 0.01]	[−0.00; 0.0
Reaction (target): Appealing to norms	−0.00	0.00	−0.01	0.00	0.01	−0.00	−0.01	−0.01	0.00	0.00	−0.00
	[−0.01; 0.01]	[−0.01; 0.01]	[−0.03; 0.01]	[−0.02; 0.03]	[−0.01; 0.02]	[−0.02; 0.02]	[−0.02; 0.00]	[−0.02; 0.01]	[−0.01; 0.01]	[−0.01; 0.02]	[−0.01; 0.0
Reaction (target): Counter aggression	0.02*	0.02*	−0.04*	0.03*	0.02	−0.00	0.00	−0.01	−0.00	0.01	−0.01
	[0.01; 0.03]	[0.01; 0.03]	[−0.06; −0.02]	[0.01; 0.05]	[−0.00; 0.03]	[−0.02; 0.02]	[−0.01; 0.02]	[−0.03; 0.01]	[−0.01; 0.01]	[−0.01; 0.03]	[−0.01; 0.0
Reaction (target): Platform action	0.00	0.01	−0.04*	0.02	0.02*	−0.00	0.01	−0.01	0.00	0.01	−0.00
	[−0.01; 0.01]	[−0.00; 0.02]	[−0.06; −0.03]	[−0.00; 0.04]	[0.00; 0.04]	[−0.02; 0.01]	[−0.01; 0.02]	[−0.03; 0.00]	[−0.00; 0.01]	[−0.00; 0.03]	[−0.01; 0.0
Num. obs.	10957	10849	11120	11120	11120	11120	11120	11120	11120	11120	11120
Num. groups: personid	1385	1385	1390	1390	1390	1390	1390	1390	1390	1390	1390
Num. groups: deckid	381	381	381	381	381	381	381	381	381	381	381
Var: personid (Intercept)	0.02	0.01	0.06	0.06	0.05	0.02	0.03	0.08	0.01	0.05	0.01
Var: deckid (Intercept)	0.00	0.00	0.00	0.00	0.00	0.00	0.00	0.00	0.00	0.00	0.00
Var: Residual	0.04	0.03	0.13	0.16	0.11	0.10	0.07	0.10	0.02	0.10	0.03

* Null hypothesis value outside the confidence interval.

Table S13: Average marginal component effects of content and context attributes on citizens' hate speech perceptions and preferences for action (German sample). Offensiveness and hatefulness scores were measured on five-point scales and rescaled to 0-1. Support for action was measured on a binary scale, 0-1. Linear mixed-effects models with person, vignette deck, and country random effects. 95% confidence intervals in parentheses.

	Offensive	Hateful	No action	Delete	Block	Temp. ban	Perm. ban	No penalties	Lose job	Fine	Prison
Type (severity): Discrimination (extreme)	0.06*	0.06*	−0.06*	0.02	0.01	0.03	0.02	−0.02	0.00	0.01	0.00
	[0.04; 0.07]	[0.04; 0.08]	[−0.09; −0.03]	[−0.01; 0.05]	[−0.02; 0.04]	[−0.00; 0.05]	[−0.00; 0.04]	[−0.05; 0.00]	[−0.01; 0.02]	[−0.01; 0.04]	[−0.01; 0.01]
Type (severity): Vilification (moderate)	−0.09*	−0.09*	0.12*	−0.08*	−0.08*	−0.03	−0.02	0.03*	−0.01	−0.03*	−0.00
	[−0.11; −0.08]	[−0.10; −0.07]	[0.09; 0.16]	[−0.11; −0.04]	[−0.11; −0.05]	[−0.05; 0.00]	[−0.05; 0.00]	[0.01; 0.06]	[−0.02; 0.01]	[−0.05; −0.01]	[−0.01; 0.01]
Type (severity): Vilification (extreme)	0.15*	0.15*	−0.20*	0.11*	0.09*	0.08*	0.05*	−0.06*	0.03*	0.03*	0.01
	[0.14; 0.17]	[0.13; 0.16]	[−0.23; −0.17]	[0.08; 0.15]	[0.06; 0.12]	[0.05; 0.11]	[0.03; 0.07]	[−0.08; −0.03]	[0.01; 0.04]	[0.01; 0.05]	[−0.00; 0.02]
Type (severity): Insult (moderate)	0.07*	0.06*	−0.09*	0.06*	0.01	0.03*	0.02	−0.01	0.01	0.01	0.00
	[0.05; 0.08]	[0.05; 0.08]	[−0.12; −0.06]	[0.03; 0.09]	[−0.02; 0.04]	[0.00; 0.05]	[−0.00; 0.04]	[−0.04; 0.01]	[−0.01; 0.02]	[−0.01; 0.03]	[−0.01; 0.01]
Type (severity): Insult (extreme)	0.18*	0.18*	−0.28*	0.19*	0.12*	0.10*	0.06*	−0.06*	0.02*	0.04*	0.01
	[0.16; 0.19]	[0.17; 0.20]	[−0.31; −0.25]	[0.16; 0.22]	[0.09; 0.14]	[0.08; 0.12]	[0.04; 0.08]	[−0.08; −0.03]	[0.01; 0.03]	[0.02; 0.06]	[−0.00; 0.02]
Type (severity): Violence (moderate)	0.21*	0.24*	−0.37*	0.22*	0.15*	0.13*	0.10*	−0.12*	0.04*	0.08*	0.01*
	[0.20; 0.23]	[0.22; 0.25]	[−0.40; −0.34]	[0.19; 0.25]	[0.12; 0.18]	[0.11; 0.16]	[0.07; 0.12]	[−0.14; −0.09]	[0.03; 0.05]	[0.06; 0.10]	[0.00; 0.02]
Type (severity): Violence (extreme)	0.37*	0.40*	−0.53*	0.24*	0.20*	0.17*	0.34*	−0.28*	0.09*	0.18*	0.07*
	[0.35; 0.38]	[0.39; 0.41]	[−0.56; −0.50]	[0.21; 0.27]	[0.17; 0.23]	[0.14; 0.19]	[0.32; 0.36]	[−0.30; −0.26]	[0.07; 0.10]	[0.16; 0.20]	[0.06; 0.08]
Topic: Liberals	−0.00	−0.01	0.01	−0.01	0.01	0.00	0.00	−0.01	0.01	0.01	−0.00
	[−0.01; 0.01]	[−0.02; 0.00]	[−0.01; 0.03]	[−0.04; 0.01]	[−0.01; 0.03]	[−0.02; 0.02]	[−0.01; 0.02]	[−0.03; 0.00]	[−0.00; 0.02]	[−0.00; 0.02]	[−0.01; 0.01]
Topic: Muslim immigrants	0.07*	0.07*	−0.10*	0.05*	0.06*	0.04*	0.05*	−0.05*	0.02*	0.02*	0.00
	[0.06; 0.08]	[0.06; 0.08]	[−0.12; −0.07]	[0.02; 0.07]	[0.04; 0.08]	[0.02; 0.06]	[0.04; 0.07]	[−0.07; −0.03]	[0.01; 0.03]	[0.01; 0.04]	[−0.00; 0.01]
Topic: Women	0.06*	0.04*	−0.07*	0.01	0.06*	0.02*	0.04*	−0.03*	0.02*	0.03*	0.00
	[0.05; 0.07]	[0.03; 0.06]	[−0.09; −0.05]	[−0.01; 0.04]	[0.04; 0.08]	[0.01; 0.04]	[0.02; 0.05]	[−0.05; −0.02]	[0.01; 0.03]	[0.01; 0.04]	[−0.00; 0.01]
Addressing scope: Extremist	0.01	0.01	−0.01	0.00	−0.00	−0.00	0.01	−0.01	0.00	0.00	−0.00
	[−0.00; 0.02]	[−0.01; 0.02]	[−0.03; 0.01]	[−0.02; 0.02]	[−0.02; 0.02]	[−0.02; 0.01]	[−0.00; 0.03]	[−0.02; 0.01]	[−0.01; 0.02]	[−0.01; 0.01]	[−0.01; 0.01]
Addressing scope: Most	0.03*	0.02*	−0.02	−0.00	−0.02	0.00	0.02*	−0.00	0.01*	−0.00	−0.00
	[0.02; 0.04]	[0.01; 0.03]	[−0.04; 0.01]	[−0.03; 0.02]	[−0.04; 0.00]	[−0.01; 0.02]	[0.01; 0.04]	[−0.02; 0.02]	[0.00; 0.02]	[−0.02; 0.01]	[−0.01; 0.01]
Addressing scope: All	0.03*	0.03*	−0.03*	0.02	−0.01	−0.00	0.02*	−0.00	0.01	0.00	0.00
	[0.02; 0.04]	[0.02; 0.04]	[−0.05; −0.01]	[−0.01; 0.04]	[−0.03; 0.01]	[−0.02; 0.02]	[0.01; 0.04]	[−0.02; 0.01]	[−0.00; 0.02]	[−0.01; 0.02]	[−0.01; 0.01]
Identity (sender): Anonymous | Liberal	0.01	0.00	−0.01	−0.01	0.01	0.00	0.00	−0.00	0.00	0.00	−0.00
	[−0.00; 0.03]	[−0.01; 0.02]	[−0.04; 0.02]	[−0.04; 0.03]	[−0.02; 0.03]	[−0.02; 0.03]	[−0.02; 0.03]	[−0.03; 0.02]	[−0.01; 0.02]	[−0.02; 0.02]	[−0.01; 0.01]
Identity (sender): Female | Muslim	0.01	0.01	−0.01	−0.01	0.02	−0.00	0.00	0.00	0.01	−0.01	−0.01
	[−0.00; 0.02]	[−0.01; 0.02]	[−0.04; 0.02]	[−0.04; 0.02]	[−0.01; 0.05]	[−0.03; 0.02]	[−0.02; 0.02]	[−0.02; 0.02]	[−0.00; 0.02]	[−0.02; 0.01]	[−0.02; 0.00]
Identity (sender): Female | Non-Muslim	0.02*	0.01	−0.01	0.00	0.01	0.01	−0.00	0.00	0.01	0.00	−0.01
	[0.00; 0.03]	[−0.01; 0.02]	[−0.04; 0.01]	[−0.02; 0.03]	[−0.02; 0.03]	[−0.01; 0.03]	[−0.02; 0.02]	[−0.02; 0.02]	[−0.01; 0.02]	[−0.02; 0.02]	[−0.01; 0.00]
Identity (sender): Male | Muslim	0.02*	0.01	−0.02	−0.01	0.01	0.01	0.01	−0.01	0.00	−0.00	−0.00
	[0.00; 0.03]	[−0.00; 0.02]	[−0.05; 0.01]	[−0.04; 0.02]	[−0.02; 0.03]	[−0.01; 0.04]	[−0.01; 0.02]	[−0.03; 0.02]	[−0.01; 0.02]	[−0.02; 0.01]	[−0.01; 0.01]
Identity (sender): Male | Non-Muslim	0.00	−0.01	−0.00	0.01	0.01	−0.00	−0.02*	0.01	0.00	−0.00	−0.01
	[−0.01; 0.02]	[−0.02; 0.01]	[−0.03; 0.02]	[−0.02; 0.04]	[−0.02; 0.03]	[−0.02; 0.02]	[−0.04; −0.00]	[−0.01; 0.03]	[−0.01; 0.01]	[−0.02; 0.01]	[−0.01; 0.00]
Identity (target): Anonymous | Liberal	0.00	0.01	−0.03*	0.01	0.01	0.01	0.01	−0.00	−0.01	0.02	−0.01
	[−0.01; 0.02]	[−0.00; 0.03]	[−0.06; −0.00]	[−0.02; 0.04]	[−0.02; 0.03]	[−0.01; 0.04]	[−0.01; 0.04]	[−0.02; 0.02]	[−0.02; 0.01]	[−0.00; 0.03]	[−0.01; 0.00]
Identity (target): Female | Muslim	0.01	0.01	−0.02	0.01	0.01	−0.01	−0.00	0.01	−0.01	0.00	−0.01*
	[−0.01; 0.02]	[−0.01; 0.02]	[−0.05; 0.00]	[−0.02; 0.04]	[−0.02; 0.03]	[−0.03; 0.01]	[−0.02; 0.02]	[−0.01; 0.03]	[−0.02; 0.01]	[−0.01; 0.02]	[−0.02; −0.00]
Identity (target): Female | Non-Muslim	0.00	0.01	−0.02	0.02	0.01	0.01	−0.01	−0.01	−0.01*	0.01	−0.00
	[−0.01; 0.02]	[−0.01; 0.02]	[−0.04; 0.01]	[−0.00; 0.05]	[−0.02; 0.03]	[−0.01; 0.03]	[−0.02; 0.01]	[−0.03; 0.01]	[−0.03; −0.00]	[−0.00; 0.03]	[−0.01; 0.00]
Identity (target): Male | Muslim	0.00	0.01	−0.02	0.01	−0.00	0.00	−0.01	0.00	−0.01*	0.01	−0.01*
	[−0.01; 0.01]	[−0.01; 0.02]	[−0.04; 0.01]	[−0.01; 0.04]	[−0.03; 0.02]	[−0.02; 0.02]	[−0.03; 0.01]	[−0.02; 0.02]	[−0.03; −0.00]	[−0.00; 0.03]	[−0.02; −0.00]
Identity (target): Male | Non-Muslim	−0.01	−0.01	0.01	0.01	−0.01	−0.01	−0.00	0.01	−0.01*	−0.00	−0.01*
	[−0.02; 0.00]	[−0.02; 0.01]	[−0.02; 0.03]	[−0.02; 0.04]	[−0.03; 0.02]	[−0.03; 0.02]	[−0.02; 0.01]	[−0.01; 0.03]	[−0.03; −0.00]	[−0.02; 0.02]	[−0.02; −0.00]
Target message: Group support	−0.01	−0.01*	0.01	−0.00	−0.01*	0.01	−0.00	0.00	−0.00	−0.00	0.00
	[−0.01; 0.00]	[−0.01; −0.00]	[−0.00; 0.03]	[−0.02; 0.01]	[−0.03; −0.00]	[−0.01; 0.02]	[−0.01; 0.01]	[−0.01; 0.01]	[−0.01; 0.00]	[−0.01; 0.01]	[−0.00; 0.01]
Reaction (target): Appealing to norms	−0.00	0.00	−0.01	−0.00	0.00	0.00	0.00	−0.00	−0.00	−0.00	0.00
	[−0.01; 0.01]	[−0.01; 0.01]	[−0.03; 0.01]	[−0.02; 0.02]	[−0.01; 0.02]	[−0.01; 0.02]	[−0.01; 0.02]	[−0.02; 0.01]	[−0.01; 0.01]	[−0.02; 0.01]	[−0.00; 0.01]
Reaction (target): Counter aggression	0.00	0.00	−0.01	0.01	−0.00	−0.00	0.01	−0.01	0.01	0.01	0.00
	[−0.01; 0.01]	[−0.01; 0.01]	[−0.03; 0.01]	[−0.01; 0.03]	[−0.02; 0.02]	[−0.02; 0.01]	[−0.01; 0.02]	[−0.03; 0.00]	[−0.00; 0.02]	[−0.01; 0.02]	[−0.00; 0.01]
Reaction (target): Platform action	0.00	0.01	−0.03*	0.00	0.02	0.01	0.01	0.00	0.00	−0.00	0.00
	[−0.01; 0.01]	[−0.00; 0.01]	[−0.05; −0.01]	[−0.02; 0.02]	[−0.00; 0.04]	[−0.00; 0.03]	[−0.00; 0.02]	[−0.01; 0.01]	[−0.01; 0.01]	[−0.02; 0.01]	[−0.00; 0.01]
Num. obs.	9803	9740	9856	9856	9856	9856	9856	9856	9856	9856	9856
Num. groups: personid	1232	1232	1232	1232	1232	1232	1232	1232	1232	1232	1232
Num. groups: deckid	387	387	387	387	387	387	387	387	387	387	387
Var: personid (Intercept)	0.02	0.02	0.09	0.08	0.06	0.03	0.02	0.05	0.01	0.03	0.00
Var: deckid (Intercept)	0.00	0.00	0.00	0.00	0.00	0.00	0.00	0.00	0.00	0.00	0.00
Var: Residual	0.03	0.03	0.11	0.13	0.10	0.08	0.06	0.07	0.03	0.05	0.01

* Null hypothesis value outside the confidence interval.

Table S14: Average marginal component effects of content and context attributes on citizens' hate speech perceptions and preferences for action (U.S. sample). Offensiveness and hatefulness scores were measured on five-point scales and rescaled to 0-1. Support for action was measured on a binary scale, 0-1. Linear mixed-effects models with person, vignette deck, and country random effects. 95% confidence intervals in parentheses.

	Hatefulness score	Platform sanctions	Other sanctions
Type (severity): Discrimination (extreme)	0.05*	0.07*	0.01
	[0.04; 0.06]	[0.04; 0.09]	[−0.01; 0.03]
Type (severity): Vilification (moderate)	−0.07*	−0.08*	−0.02*
	[−0.08; −0.06]	[−0.11; −0.06]	[−0.04; −0.00]
Type (severity): Vilification (extreme)	0.14*	0.26*	0.08*
	[0.13; 0.15]	[0.23; 0.28]	[0.06; 0.10]
Type (severity): Insult (moderate)	0.09*	0.20*	0.05*
	[0.08; 0.10]	[0.17; 0.22]	[0.04; 0.07]
Type (severity): Insult (extreme)	0.18*	0.34*	0.09*
	[0.17; 0.19]	[0.32; 0.36]	[0.07; 0.11]
Type (severity): Violence (moderate)	0.26*	0.42*	0.16*
	[0.25; 0.27]	[0.40; 0.44]	[0.15; 0.18]
Type (severity): Violence (extreme)	0.42*	0.55*	0.36*
	[0.40; 0.43]	[0.53; 0.57]	[0.34; 0.38]
Topic: Liberals	−0.00	0.01	0.02*
	[−0.01; 0.01]	[−0.01; 0.02]	[0.01; 0.03]
Topic: Muslim immigrants	0.07*	0.09*	0.05*
	[0.06; 0.08]	[0.08; 0.11]	[0.04; 0.07]
Topic: Women	0.04*	0.05*	0.05*
	[0.03; 0.04]	[0.04; 0.07]	[0.04; 0.07]
Addressing scope: Extremist	0.01*	−0.01	0.01
	[0.00; 0.02]	[−0.03; 0.00]	[−0.00; 0.02]
Addressing scope: Most	0.02*	−0.00	0.01
	[0.01; 0.02]	[−0.02; 0.01]	[−0.01; 0.02]
Addressing scope: All	0.03*	0.02*	0.02*
	[0.02; 0.04]	[0.00; 0.03]	[0.00; 0.03]
Identity (sender): Anonymous | Liberal	−0.00	−0.01	0.01
	[−0.01; 0.01]	[−0.03; 0.01]	[−0.01; 0.03]
Identity (sender): Female | Muslim	0.00	−0.00	−0.01
	[−0.01; 0.01]	[−0.02; 0.02]	[−0.02; 0.01]
Identity (sender): Female | Non-Muslim	−0.01	−0.02*	−0.02*
	[−0.02; 0.00]	[−0.04; −0.00]	[−0.03; −0.00]
Identity (sender): Male | Muslim	0.01	0.01	−0.00
	[−0.00; 0.02]	[−0.01; 0.02]	[−0.02; 0.01]
Identity (sender): Male | Non-Muslim	−0.01	−0.01	−0.01
	[−0.02; 0.00]	[−0.03; 0.01]	[−0.02; 0.01]
Identity (target): Anonymous | Liberal	0.00	0.00	0.01
	[−0.01; 0.01]	[−0.02; 0.02]	[−0.01; 0.02]
Identity (target): Female | Muslim	0.00	0.01	−0.00
	[−0.01; 0.01]	[−0.01; 0.02]	[−0.02; 0.01]
Identity (target): Female | Non-Muslim	−0.00	−0.00	−0.00
	[−0.01; 0.01]	[−0.02; 0.02]	[−0.02; 0.01]
Identity (target): Male | Muslim	−0.00	−0.01	−0.00
	[−0.01; 0.01]	[−0.03; 0.01]	[−0.02; 0.01]
Identity (target): Male | Non-Muslim	−0.01	−0.01	−0.01
	[−0.02; 0.00]	[−0.03; 0.01]	[−0.03; 0.00]
Target message: Group support	−0.01*	−0.01	0.00
	[−0.01; −0.00]	[−0.02; 0.00]	[−0.01; 0.01]
Reaction (target): Appealing to norms	0.00	0.01	0.00
	[−0.00; 0.01]	[−0.00; 0.03]	[−0.01; 0.01]
Reaction (target): Counter aggression	0.01*	0.03*	0.01*
	[0.00; 0.02]	[0.02; 0.04]	[0.00; 0.02]
Reaction (target): Platform action	0.01*	0.04*	0.01
	[0.00; 0.01]	[0.02; 0.05]	[−0.00; 0.02]
Num. obs.	20589	20976	20976
Num. groups: personid	2617	2622	2622
Num. groups: deckid	768	768	768
Num. groups: country	2	2	2
Var: personid (Intercept)	0.02	0.08	0.06
Var: deckid (Intercept)	0.00	0.00	0.00
Var: country (Intercept)	0.00	0.00	0.01
Var: Residual	0.03	0.12	0.08

* Null hypothesis value outside the confidence interval.

Table S15: Average marginal component effects of content and context attributes on citizens' hate speech perceptions and preferences for action (pooled sample). Hatefulness scores were measured on five-point scales and rescaled to 0-1. The choice of platform sanctions is encoded as binary decision that takes the value 1 if the respondent chose at least one of the following options: delete post, block sender, temporarily ban sender, permanently ban sender. The choice of other sanctions is encoded as binary decision that takes the value 1 if the respondent chose at least one of the following options: lose job, pay fine, face prison sentence. Linear mixed-effects models with person, vignette deck, and country random effects. 95% confidence intervals in parentheses.

	Hatefulness score	Platform sanctions	Other sanctions
Type (severity): Discrimination (extreme)	0.04*	0.07*	−0.00
	[0.02; 0.05]	[0.04; 0.10]	[−0.03; 0.03]
Type (severity): Vilification (moderate)	−0.06*	−0.05*	−0.01
	[−0.08; −0.04]	[−0.08; −0.01]	[−0.03; 0.02]
Type (severity): Vilification (extreme)	0.13*	0.31*	0.11*
	[0.12; 0.15]	[0.27; 0.34]	[0.08; 0.14]
Type (severity): Insult (moderate)	0.12*	0.29*	0.08*
	[0.10; 0.13]	[0.26; 0.32]	[0.06; 0.11]
Type (severity): Insult (extreme)	0.17*	0.40*	0.12*
	[0.16; 0.19]	[0.37; 0.42]	[0.09; 0.14]
Type (severity): Violence (moderate)	0.27*	0.47*	0.20*
	[0.26; 0.29]	[0.44; 0.49]	[0.18; 0.23]
Type (severity): Violence (extreme)	0.43*	0.58*	0.45*
	[0.41; 0.44]	[0.55; 0.60]	[0.42; 0.47]
Topic: Liberals	0.00	0.02	0.02
	[−0.01; 0.01]	[−0.00; 0.04]	[−0.00; 0.03]
Topic: Muslim immigrants	0.07*	0.09*	0.06*
	[0.06; 0.08]	[0.07; 0.12]	[0.04; 0.08]
Topic: Women	0.03*	0.04*	0.07*
	[0.02; 0.04]	[0.02; 0.06]	[0.05; 0.09]
Addressing scope: Extremist	0.02*	−0.03*	0.01
	[0.00; 0.03]	[−0.05; −0.01]	[−0.01; 0.03]
Addressing scope: Most	0.01	−0.01	0.00
	[−0.00; 0.02]	[−0.03; 0.01]	[−0.01; 0.02]
Addressing scope: All	0.03*	0.01	0.02
	[0.02; 0.04]	[−0.01; 0.03]	[−0.00; 0.04]
Identity (sender): Anonymous | Liberal	−0.01	−0.02	0.01
	[−0.02; 0.01]	[−0.05; 0.01]	[−0.02; 0.04]
Identity (sender): Female | Muslim	−0.00	−0.02	−0.01
	[−0.02; 0.01]	[−0.05; 0.01]	[−0.04; 0.01]
Identity (sender): Female | Non-Muslim	−0.02*	−0.05*	−0.04*
	[−0.03; −0.01]	[−0.08; −0.02]	[−0.06; −0.01]
Identity (sender): Male | Muslim	0.00	−0.01	−0.01
	[−0.01; 0.02]	[−0.03; 0.02]	[−0.03; 0.02]
Identity (sender): Male | Non-Muslim	−0.01	−0.02	−0.01
	[−0.02; 0.01]	[−0.04; 0.01]	[−0.04; 0.01]
Identity (target): Anonymous | Liberal	−0.00	−0.02	0.01
	[−0.02; 0.01]	[−0.05; 0.01]	[−0.02; 0.03]
Identity (target): Female | Muslim	0.00	−0.00	0.01
	[−0.01; 0.01]	[−0.03; 0.02]	[−0.02; 0.03]
Identity (target): Female | Non-Muslim	−0.01	−0.01	−0.00
	[−0.02; 0.01]	[−0.03; 0.02]	[−0.03; 0.02]
Identity (target): Male | Muslim	−0.01	−0.03*	0.00
	[−0.03; 0.00]	[−0.06; −0.01]	[−0.02; 0.03]
Identity (target): Male | Non-Muslim	−0.01	−0.01	−0.00
	[−0.02; 0.00]	[−0.03; 0.02]	[−0.03; 0.02]
Target message: Group support	−0.01	−0.01	0.00
	[−0.01; 0.00]	[−0.02; 0.01]	[−0.01; 0.01]
Reaction (target): Appealing to norms	0.00	0.01	0.00
	[−0.01; 0.01]	[−0.01; 0.03]	[−0.01; 0.02]
Reaction (target): Counter aggression	0.02*	0.04*	0.01
	[0.01; 0.03]	[0.02; 0.06]	[−0.01; 0.03]
Reaction (target): Platform action	0.01	0.04*	0.02*
	[−0.00; 0.02]	[0.03; 0.06]	[0.00; 0.03]
Num. obs.	10849	11120	11120
Num. groups: personid	1385	1390	1390
Num. groups: deckid	381	381	381
Var: personid (Intercept)	0.01	0.06	0.07
Var: deckid (Intercept)	0.00	0.00	0.00
Var: Residual	0.03	0.13	0.10

* Null hypothesis value outside the confidence interval.

Table S16: Average marginal component effects of content and context attributes on citizens' hate speech perceptions and preferences for action (German sample). Hatefulness scores were measured on five-point scales and rescaled to 0-1. The choice of platform sanctions is encoded as binary decision that takes the value 1 if the respondent chose at least one of the following options: delete post, block sender, temporarily ban sender, permanently ban sender. The choice of other sanctions is encoded as binary decision that takes the value 1 if the respondent chose at least one of the following options: lose job, pay fine, face prison sentence. Linear mixed-effects models with person, vignette deck, and country random effects. 95% confidence intervals in parentheses.

	Hatefulness score	Platform sanctions	Other sanctions
Type (severity): Discrimination (extreme)	0.06*	0.06*	0.02
	[0.04; 0.08]	[0.03; 0.09]	[−0.00; 0.04]
Type (severity): Vilification (moderate)	−0.09*	−0.13*	−0.04*
	[−0.10; −0.07]	[−0.16; −0.10]	[−0.06; −0.01]
Type (severity): Vilification (extreme)	0.15*	0.20*	0.05*
	[0.13; 0.16]	[0.17; 0.23]	[0.03; 0.08]
Type (severity): Insult (moderate)	0.06*	0.09*	0.01
	[0.05; 0.08]	[0.06; 0.11]	[−0.01; 0.04]
Type (severity): Insult (extreme)	0.18*	0.28*	0.05*
	[0.17; 0.20]	[0.25; 0.30]	[0.03; 0.07]
Type (severity): Violence (moderate)	0.24*	0.37*	0.12*
	[0.22; 0.25]	[0.34; 0.40]	[0.09; 0.14]
Type (severity): Violence (extreme)	0.40*	0.53*	0.26*
	[0.39; 0.41]	[0.50; 0.56]	[0.24; 0.28]
Topic: Liberals	−0.01	−0.01	0.02*
	[−0.02; 0.00]	[−0.03; 0.01]	[0.00; 0.03]
Topic: Muslim immigrants	0.07*	0.10*	0.05*
	[0.06; 0.08]	[0.08; 0.12]	[0.03; 0.06]
Topic: Women	0.04*	0.07*	0.04*
	[0.03; 0.06]	[0.05; 0.09]	[0.02; 0.05]
Addressing scope: Extremist	0.01	0.01	0.01
	[−0.01; 0.02]	[−0.01; 0.03]	[−0.01; 0.02]
Addressing scope: Most	0.02*	0.01	0.01
	[0.01; 0.03]	[−0.01; 0.04]	[−0.01; 0.02]
Addressing scope: All	0.03*	0.03*	0.01
	[0.02; 0.04]	[0.01; 0.05]	[−0.00; 0.03]
Identity (sender): Anonymous | Liberal	0.00	0.01	0.01
	[−0.01; 0.02]	[−0.02; 0.04]	[−0.01; 0.03]
Identity (sender): Female | Muslim	0.01	0.02	0.00
	[−0.01; 0.02]	[−0.01; 0.04]	[−0.02; 0.02]
Identity (sender): Female | Non-Muslim	0.01	0.01	0.00
	[−0.01; 0.02]	[−0.01; 0.04]	[−0.02; 0.02]
Identity (sender): Male | Muslim	0.01	0.02	0.00
	[−0.00; 0.02]	[−0.01; 0.04]	[−0.01; 0.02]
Identity (sender): Male | Non-Muslim	−0.01	0.00	0.00
	[−0.02; 0.01]	[−0.02; 0.03]	[−0.02; 0.02]
Identity (target): Anonymous | Liberal	0.01	0.03	0.00
	[−0.00; 0.03]	[−0.00; 0.05]	[−0.02; 0.02]
Identity (target): Female | Muslim	0.01	0.02	−0.01
	[−0.01; 0.02]	[−0.01; 0.05]	[−0.03; 0.01]
Identity (target): Female | Non-Muslim	0.01	0.01	−0.00
	[−0.01; 0.02]	[−0.01; 0.04]	[−0.02; 0.02]
Identity (target): Male | Muslim	0.01	0.01	−0.01
	[−0.01; 0.02]	[−0.01; 0.04]	[−0.02; 0.01]
Identity (target): Male | Non-Muslim	−0.01	−0.01	−0.02*
	[−0.02; 0.01]	[−0.03; 0.02]	[−0.04; −0.00]
Target message: Group support	−0.01*	−0.01	−0.00
	[−0.01; −0.00]	[−0.03; 0.00]	[−0.01; 0.01]
Reaction (target): Appealing to norms	0.00	0.01	−0.00
	[−0.01; 0.01]	[−0.01; 0.03]	[−0.01; 0.01]
Reaction (target): Counter aggression	0.00	0.01	0.01
	[−0.01; 0.01]	[−0.01; 0.03]	[−0.00; 0.02]
Reaction (target): Platform action	0.01	0.03*	−0.00
	[−0.00; 0.01]	[0.01; 0.05]	[−0.02; 0.01]
Num. obs.	9740	9856	9856
Num. groups: personid	1232	1232	1232
Num. groups: deckid	387	387	387
Var: personid (Intercept)	0.02	0.09	0.05
Var: deckid (Intercept)	0.00	0.00	0.00
Var: Residual	0.03	0.11	0.06

* Null hypothesis value outside the confidence interval.

Table S17: Average marginal component effects of content and context attributes on citizens' hate speech perceptions and preferences for action (U.S. sample). Hatefulness scores were measured on five-point scales and rescaled to 0-1. The choice of platform sanctions is encoded as binary decision that takes the value 1 if the respondent chose at least one of the following options: delete post, block sender, temporarily ban sender, permanently ban sender. The choice of other sanctions is encoded as binary decision that takes the value 1 if the respondent chose at least one of the following options: lose job, pay fine, face prison sentence. Linear mixed-effects models with person, vignette deck, and country random effects. 95% confidence intervals in parentheses.

	Offensive	Hateful	No action	Delete	Block	Temp. ban	Perm. ban	No penalties	Lose job	Fine	Prison
Gender: Female	0.04*	0.03*	−0.08*	0.05*	0.07*	0.02	0.02*	−0.02	−0.01	0.03*	−0.00
	[0.03; 0.06]	[0.02; 0.04]	[−0.10; −0.05]	[0.03; 0.08]	[0.05; 0.09]	[−0.00; 0.03]	[0.00; 0.03]	[−0.04; 0.01]	[−0.02; 0.00]	[0.01; 0.05]	[−0.01; 0.00]
Age: 30-49	−0.01	−0.03*	0.03	−0.01	−0.01	−0.04*	−0.00	0.03	−0.02	0.01	−0.01
	[−0.04; 0.01]	[−0.06; −0.01]	[−0.02; 0.09]	[−0.06; 0.04]	[−0.06; 0.04]	[−0.07; −0.00]	[−0.03; 0.03]	[−0.02; 0.08]	[−0.04; 0.00]	[−0.03; 0.05]	[−0.02; 0.01]
Age: 50-69	−0.00	−0.03*	0.03	0.01	−0.02	−0.04*	0.02	0.04	−0.03*	0.03	−0.01
	[−0.03; 0.02]	[−0.06; −0.01]	[−0.02; 0.09]	[−0.05; 0.06]	[−0.06; 0.03]	[−0.07; −0.00]	[−0.01; 0.05]	[−0.01; 0.09]	[−0.06; −0.01]	[−0.01; 0.07]	[−0.03; 0.00]
Age: 70+	0.03*	−0.00	0.02	0.02	−0.04	−0.04*	−0.00	0.01	−0.03*	0.04	−0.02*
	[0.00; 0.06]	[−0.03; 0.03]	[−0.04; 0.08]	[−0.04; 0.08]	[−0.09; 0.01]	[−0.08; −0.00]	[−0.04; 0.03]	[−0.05; 0.06]	[−0.06; −0.01]	[−0.00; 0.09]	[−0.04; −0.01]
Education: Intermediate	0.01	0.02	0.01	−0.01	0.01	−0.00	−0.02	0.01	0.00	0.00	−0.01
	[−0.00; 0.03]	[−0.00; 0.03]	[−0.02; 0.05]	[−0.05; 0.03]	[−0.02; 0.04]	[−0.03; 0.02]	[−0.04; 0.01]	[−0.03; 0.04]	[−0.01; 0.02]	[−0.03; 0.03]	[−0.02; 0.01]
Education: High	0.02	0.02	0.03	−0.02	0.03	0.01	−0.02*	0.04*	−0.01	−0.01	−0.02*
	[−0.00; 0.04]	[−0.00; 0.04]	[−0.01; 0.07]	[−0.06; 0.01]	[−0.00; 0.06]	[−0.01; 0.04]	[−0.05; −0.00]	[0.01; 0.08]	[−0.03; 0.01]	[−0.04; 0.01]	[−0.03; −0.00]
Political interest: Intermediate	−0.01	−0.01	0.01	0.01	0.02	0.02	−0.00	0.02	−0.02*	−0.01	−0.01
	[−0.03; 0.02]	[−0.03; 0.02]	[−0.04; 0.06]	[−0.04; 0.06]	[−0.02; 0.06]	[−0.01; 0.05]	[−0.03; 0.02]	[−0.02; 0.07]	[−0.04; −0.00]	[−0.05; 0.02]	[−0.02; 0.00]
Political interest: High	0.01	0.00	0.03	−0.01	0.01	0.01	−0.01	0.05*	−0.02*	−0.03*	−0.01
	[−0.02; 0.03]	[−0.02; 0.02]	[−0.01; 0.08]	[−0.05; 0.04]	[−0.03; 0.05]	[−0.02; 0.04]	[−0.04; 0.01]	[0.01; 0.09]	[−0.04; −0.00]	[−0.07; −0.00]	[−0.02; 0.00]
Social media user: Yes	−0.00	0.01	−0.00	0.00	−0.01	−0.00	−0.03*	0.03	0.00	−0.04*	−0.01*
	[−0.02; 0.02]	[−0.01; 0.02]	[−0.04; 0.03]	[−0.03; 0.04]	[−0.04; 0.02]	[−0.03; 0.02]	[−0.05; −0.00]	[−0.00; 0.06]	[−0.01; 0.02]	[−0.06; −0.01]	[−0.02; −0.00]
Ideology: Center	−0.01	−0.02*	0.01	−0.01	−0.01	−0.00	−0.01	−0.01	0.00	0.01	−0.00
	[−0.03; 0.00]	[−0.03; −0.01]	[−0.02; 0.04]	[−0.04; 0.02]	[−0.04; 0.01]	[−0.02; 0.01]	[−0.03; 0.01]	[−0.04; 0.01]	[−0.01; 0.02]	[−0.01; 0.03]	[−0.01; 0.01]
Ideology: Right	−0.07*	−0.06*	0.13*	−0.11*	−0.07*	−0.05*	−0.02*	0.05*	0.01	−0.04*	0.00
	[−0.09; −0.06]	[−0.08; −0.05]	[0.10; 0.17]	[−0.14; −0.07]	[−0.09; −0.04]	[−0.07; −0.03]	[−0.04; −0.00]	[0.01; 0.08]	[−0.00; 0.02]	[−0.07; −0.02]	[−0.00; 0.01]
Hate experience: Yes	−0.01	−0.00	0.04*	−0.05*	−0.02	−0.01	−0.01	−0.01	0.01	−0.01	0.01
	[−0.03; 0.01]	[−0.02; 0.02]	[0.01; 0.08]	[−0.08; −0.01]	[−0.06; 0.01]	[−0.04; 0.01]	[−0.03; 0.01]	[−0.05; 0.02]	[−0.01; 0.02]	[−0.03; 0.02]	[−0.00; 0.02]
Hate witness: Yes	0.00	0.00	0.01	−0.01	0.04*	0.02*	−0.01	0.03	0.00	0.00	−0.01
	[−0.01; 0.02]	[−0.01; 0.02]	[−0.02; 0.04]	[−0.03; 0.02]	[0.01; 0.06]	[0.01; 0.04]	[−0.03; 0.01]	[−0.00; 0.05]	[−0.01; 0.01]	[−0.02; 0.02]	[−0.02; 0.00]
Talk politics freely: Yes	−0.00	−0.01	0.01	0.01	−0.01	−0.01	−0.00	0.03*	−0.01	−0.01	−0.00
	[−0.02; 0.01]	[−0.02; 0.00]	[−0.02; 0.03]	[−0.02; 0.03]	[−0.04; 0.01]	[−0.03; 0.01]	[−0.02; 0.01]	[0.00; 0.05]	[−0.02; 0.00]	[−0.03; 0.01]	[−0.01; 0.00]
Num. obs.	17497	17343	17656	17656	17656	17656	17656	17656	17656	17656	17656
Num. groups: personid	2204	2204	2207	2207	2207	2207	2207	2207	2207	2207	2207
Num. groups: deckid	747	747	747	747	747	747	747	747	747	747	747
Num. groups: country	2	2	2	2	2	2	2	2	2	2	2
Var: personid (Intercept)	0.01	0.01	0.06	0.07	0.05	0.03	0.02	0.06	0.01	0.04	0.00
Var: deckid (Intercept)	0.00	0.00	0.00	0.00	0.00	0.00	0.00	0.00	0.00	0.00	0.00
Var: country (Intercept)	0.00	0.00	0.00	0.00	0.00	0.00	0.00	0.01	0.00	0.01	0.00
Var: Residual	0.05	0.05	0.17	0.16	0.11	0.10	0.08	0.10	0.02	0.09	0.02

* Null hypothesis value outside the confidence interval.

Table S18: Effects of respondent characteristics on hate speech perceptions and preferences for action (pooled sample). Offensiveness and hatefulness scores were measured on five-point scales and rescaled to 0-1. Support for action was measured on a binary scale, 0-1. Linear mixed-effects models with person, vignette deck, and country random effects. 95% confidence intervals in parentheses.

	Offensive	Hateful	No action	Delete	Block	Temp. ban	Perm. ban	No penalties	Lose job	Fine	Prison
Gender: Female	0.06*	0.04*	−0.07*	0.04	0.09*	0.01	0.02	−0.01	−0.01	0.02	0.00
	[0.04; 0.07]	[0.02; 0.05]	[−0.10; −0.03]	[−0.00; 0.07]	[0.05; 0.12]	[−0.01; 0.04]	[−0.01; 0.04]	[−0.05; 0.03]	[−0.02; 0.01]	[−0.01; 0.05]	[−0.01; 0.01]
Age: 30-49	0.01	−0.03	−0.01	0.01	−0.01	−0.02	0.00	0.01	−0.01	0.03	−0.01
	[−0.03; 0.04]	[−0.06; 0.00]	[−0.07; 0.06]	[−0.06; 0.07]	[−0.07; 0.05]	[−0.06; 0.02]	[−0.04; 0.05]	[−0.06; 0.08]	[−0.04; 0.01]	[−0.02; 0.09]	[−0.04; 0.01]
Age: 50-69	0.01	−0.04*	−0.01	0.00	0.00	−0.01	0.02	0.02	−0.02	0.05	−0.03*
	[−0.02; 0.04]	[−0.07; −0.00]	[−0.07; 0.06]	[−0.06; 0.07]	[−0.06; 0.06]	[−0.05; 0.04]	[−0.03; 0.06]	[−0.05; 0.09]	[−0.05; 0.00]	[−0.01; 0.10]	[−0.06; −0.01]
Age: 70+	0.03	−0.01	−0.07	0.06	0.00	−0.01	−0.01	−0.03	−0.02	0.07	−0.04*
	[−0.02; 0.07]	[−0.05; 0.04]	[−0.15; 0.02]	[−0.02; 0.15]	[−0.08; 0.08]	[−0.07; 0.05]	[−0.07; 0.05]	[−0.12; 0.07]	[−0.05; 0.02]	[−0.00; 0.14]	[−0.08; −0.01]
Education: Intermediate	0.01	0.03	−0.00	−0.00	−0.02	−0.01	0.00	−0.01	0.01	0.01	0.00
	[−0.01; 0.04]	[−0.00; 0.05]	[−0.05; 0.05]	[−0.05; 0.05]	[−0.07; 0.03]	[−0.05; 0.02]	[−0.03; 0.04]	[−0.07; 0.04]	[−0.02; 0.03]	[−0.03; 0.06]	[−0.02; 0.02]
Education: High	0.01	0.02	0.03	−0.01	−0.00	0.01	−0.01	0.03	−0.01	−0.00	−0.02
	[−0.01; 0.04]	[−0.00; 0.05]	[−0.02; 0.08]	[−0.06; 0.05]	[−0.05; 0.05]	[−0.03; 0.04]	[−0.05; 0.02]	[−0.02; 0.09]	[−0.03; 0.01]	[−0.05; 0.04]	[−0.04; 0.00]
Political interest: Intermediate	−0.01	−0.00	0.03	0.02	0.04	−0.00	−0.03	0.09*	−0.04*	−0.02	−0.01
	[−0.05; 0.03]	[−0.04; 0.03]	[−0.04; 0.10]	[−0.06; 0.10]	[−0.02; 0.11]	[−0.05; 0.05]	[−0.08; 0.01]	[0.01; 0.17]	[−0.06; −0.01]	[−0.08; 0.05]	[−0.03; 0.02]
Political interest: High	0.00	0.00	0.06	−0.01	0.03	−0.02	−0.05	0.11*	−0.03*	−0.04	−0.01
	[−0.03; 0.04]	[−0.03; 0.04]	[−0.01; 0.13]	[−0.08; 0.07]	[−0.03; 0.10]	[−0.07; 0.03]	[−0.09; 0.00]	[0.04; 0.19]	[−0.06; −0.00]	[−0.10; 0.02]	[−0.03; 0.02]
Social media user: Yes	−0.01	0.00	−0.00	0.01	−0.01	−0.01	−0.02	0.03	0.01	−0.04*	−0.02*
	[−0.03; 0.01]	[−0.02; 0.02]	[−0.04; 0.04]	[−0.03; 0.05]	[−0.05; 0.03]	[−0.04; 0.02]	[−0.05; 0.00]	[−0.01; 0.08]	[−0.00; 0.03]	[−0.07; −0.00]	[−0.04; −0.01]
Ideology: Center	0.00	−0.00	−0.02	0.01	0.01	0.03*	−0.03	−0.02	0.03*	0.00	0.00
	[−0.02; 0.02]	[−0.02; 0.02]	[−0.06; 0.02]	[−0.04; 0.05]	[−0.03; 0.04]	[0.00; 0.06]	[−0.06; 0.00]	[−0.06; 0.03]	[0.01; 0.05]	[−0.04; 0.04]	[−0.01; 0.02]
Ideology: Right	−0.04*	−0.02	0.04	−0.04	−0.01	−0.01	−0.01	0.02	0.07*	−0.05	0.01
	[−0.07; −0.00]	[−0.06; 0.01]	[−0.02; 0.10]	[−0.11; 0.02]	[−0.06; 0.05]	[−0.05; 0.03]	[−0.06; 0.03]	[−0.05; 0.08]	[0.05; 0.10]	[−0.10; 0.00]	[−0.02; 0.03]
Hate experience: Yes	−0.00	0.01	0.01	−0.04	0.03	0.01	−0.01	−0.08*	0.03*	0.01	0.02
	[−0.03; 0.03]	[−0.02; 0.04]	[−0.05; 0.07]	[−0.10; 0.03]	[−0.03; 0.08]	[−0.03; 0.05]	[−0.05; 0.03]	[−0.14; −0.02]	[0.00; 0.05]	[−0.04; 0.06]	[−0.01; 0.04]
Hate witness: Yes	0.00	0.00	−0.01	−0.00	0.04*	0.03*	−0.00	0.00	0.00	0.04	−0.01
	[−0.02; 0.02]	[−0.02; 0.02]	[−0.05; 0.03]	[−0.05; 0.04]	[0.00; 0.08]	[0.00; 0.06]	[−0.03; 0.02]	[−0.04; 0.05]	[−0.02; 0.02]	[−0.00; 0.07]	[−0.03; 0.00]
Talk politics freely: Yes	−0.01	−0.01	0.04	0.01	−0.02	−0.00	0.00	0.06*	−0.01	−0.04*	0.00
	[−0.03; 0.01]	[−0.03; 0.01]	[−0.00; 0.07]	[−0.03; 0.05]	[−0.05; 0.02]	[−0.03; 0.02]	[−0.02; 0.03]	[0.02; 0.11]	[−0.03; 0.00]	[−0.07; −0.00]	[−0.01; 0.02]
Party ID: SPD	0.00	−0.00	−0.00	−0.03	0.02	0.02	−0.00	−0.02	0.02	−0.02	0.02*
	[−0.03; 0.03]	[−0.03; 0.03]	[−0.06; 0.05]	[−0.09; 0.03]	[−0.03; 0.08]	[−0.02; 0.06]	[−0.04; 0.04]	[−0.09; 0.04]	[−0.00; 0.04]	[−0.07; 0.03]	[0.00; 0.05]
Party ID: FDP	−0.01	−0.01	−0.01	−0.01	0.00	0.03	0.01	0.02	0.01	−0.02	0.01
	[−0.04; 0.02]	[−0.04; 0.02]	[−0.07; 0.06]	[−0.08; 0.06]	[−0.06; 0.06]	[−0.02; 0.07]	[−0.04; 0.05]	[−0.05; 0.10]	[−0.02; 0.03]	[−0.08; 0.03]	[−0.02; 0.03]
Party ID: Greens	0.02	0.01	−0.03	0.03	0.05	0.06*	−0.00	−0.01	0.02	0.00	0.00
	[−0.01; 0.05]	[−0.02; 0.04]	[−0.09; 0.02]	[−0.03; 0.09]	[−0.01; 0.10]	[0.02; 0.10]	[−0.04; 0.04]	[−0.07; 0.05]	[−0.01; 0.04]	[−0.05; 0.05]	[−0.02; 0.02]
Party ID: Left	−0.00	0.01	0.02	−0.01	−0.01	0.04	−0.05*	0.02	0.02	−0.01	0.01
	[−0.03; 0.03]	[−0.02; 0.04]	[−0.04; 0.08]	[−0.08; 0.05]	[−0.07; 0.05]	[−0.00; 0.09]	[−0.09; −0.00]	[−0.05; 0.08]	[−0.00; 0.04]	[−0.07; 0.04]	[−0.01; 0.03]
Party ID: AfD	−0.05*	−0.04*	0.14*	−0.10*	−0.02	−0.01	−0.02	0.07*	−0.02	−0.06*	0.04*
	[−0.08; −0.02]	[−0.07; −0.01]	[0.08; 0.20]	[−0.16; −0.04]	[−0.07; 0.03]	[−0.05; 0.03]	[−0.06; 0.02]	[0.01; 0.14]	[−0.04; 0.00]	[−0.11; −0.01]	[0.01; 0.06]
Party ID: Others	−0.01	−0.00	0.09*	0.00	0.02	−0.03	−0.04	0.07	−0.02	−0.07	−0.01
	[−0.05; 0.04]	[−0.05; 0.04]	[0.00; 0.18]	[−0.09; 0.10]	[−0.06; 0.11]	[−0.09; 0.03]	[−0.10; 0.02]	[−0.03; 0.17]	[−0.05; 0.02]	[−0.15; 0.00]	[−0.05; 0.02]
Num. obs.	8344	8260	8432	8432	8432	8432	8432	8432	8432	8432	8432
Num. groups: personid	1051	1051	1054	1054	1054	1054	1054	1054	1054	1054	1054
Num. groups: deckid	364	364	364	364	364	364	364	364	364	364	364
Var: personid (Intercept)	0.01	0.01	0.05	0.05	0.05	0.02	0.02	0.07	0.01	0.04	0.01
Var: deckid (Intercept)	0.00	0.00	0.00	0.00	0.00	0.00	0.00	0.00	0.00	0.00	0.00
Var: Residual	0.06	0.06	0.18	0.18	0.12	0.11	0.08	0.13	0.02	0.12	0.03

* Null hypothesis value outside the confidence interval.

Table S19: Effects of respondent characteristics on hate speech perceptions and preferences for action (German sample). Offensiveness and hatefulness scores were measured on five-point scales and rescaled to 0-1. Support for action was measured on a binary scale, 0-1. Linear mixed-effects models with person, vignette deck, and country random effects. 95% confidence intervals in parentheses.

	Offensive	Hateful	No action	Delete	Block	Temp. ban	Perm. ban	No penalties	Lose job	Fine	Prison
Gender: Female	0.03*	0.02*	−0.07*	0.05*	0.06*	0.02	0.02	−0.03	−0.01	0.04*	−0.01
	[0.01; 0.05]	[0.00; 0.04]	[−0.11; −0.03]	[0.01; 0.09]	[0.02; 0.09]	[−0.01; 0.05]	[−0.01; 0.04]	[−0.06; 0.00]	[−0.03; 0.00]	[0.01; 0.06]	[−0.01; 0.00]
Age: 30-49	−0.03	−0.03	0.05	−0.03	0.03	−0.02	−0.03	0.07	−0.03	−0.02	−0.00
	[−0.09; 0.02]	[−0.08; 0.02]	[−0.06; 0.16]	[−0.14; 0.07]	[−0.06; 0.12]	[−0.09; 0.05]	[−0.09; 0.03]	[−0.02; 0.15]	[−0.07; 0.02]	[−0.09; 0.04]	[−0.03; 0.02]
Age: 50-69	−0.01	−0.01	0.04	0.03	0.02	−0.03	−0.00	0.09*	−0.04	−0.01	−0.00
	[−0.06; 0.04]	[−0.06; 0.03]	[−0.07; 0.15]	[−0.08; 0.13]	[−0.07; 0.11]	[−0.10; 0.04]	[−0.06; 0.06]	[0.00; 0.17]	[−0.09; 0.00]	[−0.07; 0.06]	[−0.02; 0.02]
Age: 70+	0.03	0.02	0.05	0.01	−0.01	−0.04	−0.02	0.05	−0.04	0.01	−0.01
	[−0.02; 0.09]	[−0.03; 0.07]	[−0.07; 0.16]	[−0.10; 0.12]	[−0.10; 0.08]	[−0.11; 0.04]	[−0.09; 0.04]	[−0.03; 0.14]	[−0.09; 0.00]	[−0.06; 0.07]	[−0.03; 0.01]
Education: Intermediate	0.02	0.01	0.03	−0.03	0.03	0.00	−0.03	0.01	−0.00	0.01	−0.01
	[−0.01; 0.05]	[−0.02; 0.04]	[−0.03; 0.09]	[−0.09; 0.03]	[−0.02; 0.08]	[−0.04; 0.04]	[−0.07; 0.01]	[−0.04; 0.06]	[−0.03; 0.02]	[−0.03; 0.04]	[−0.02; 0.00]
Education: High	0.02	0.01	0.04	−0.05	0.04	0.01	−0.03*	0.05*	−0.02	−0.02	−0.01*
	[−0.01; 0.04]	[−0.02; 0.04]	[−0.02; 0.11]	[−0.11; 0.00]	[−0.00; 0.09]	[−0.03; 0.04]	[−0.07; −0.00]	[0.00; 0.09]	[−0.04; 0.01]	[−0.05; 0.02]	[−0.02; −0.00]
Political interest: Intermediate	−0.02	−0.02	0.02	0.01	0.01	0.03	0.02	0.01	−0.02	−0.03	−0.01
	[−0.06; 0.02]	[−0.06; 0.02]	[−0.07; 0.11]	[−0.08; 0.09]	[−0.06; 0.08]	[−0.02; 0.09]	[−0.03; 0.07]	[−0.05; 0.08]	[−0.05; 0.02]	[−0.08; 0.02]	[−0.02; 0.01]
Political interest: High	0.01	0.00	0.02	0.02	0.01	0.02	0.01	0.03	−0.02	−0.05*	−0.01
	[−0.03; 0.04]	[−0.03; 0.03]	[−0.05; 0.09]	[−0.05; 0.09]	[−0.05; 0.07]	[−0.02; 0.07]	[−0.03; 0.05]	[−0.03; 0.08]	[−0.04; 0.01]	[−0.09; −0.01]	[−0.02; 0.00]
Social media user: Yes	0.03	0.03*	−0.01	−0.00	0.00	0.00	−0.03	0.04	0.01	−0.05*	−0.01
	[−0.00; 0.06]	[0.00; 0.06]	[−0.08; 0.06]	[−0.07; 0.06]	[−0.05; 0.06]	[−0.04; 0.04]	[−0.07; 0.01]	[−0.01; 0.10]	[−0.02; 0.04]	[−0.09; −0.01]	[−0.02; 0.01]
Ideology: Center	−0.02	−0.02	−0.02	0.00	−0.01	−0.02	0.02	−0.06*	−0.00	0.05*	0.01
	[−0.04; 0.01]	[−0.04; 0.01]	[−0.07; 0.04]	[−0.05; 0.06]	[−0.05; 0.04]	[−0.06; 0.02]	[−0.01; 0.06]	[−0.11; −0.02]	[−0.03; 0.02]	[0.01; 0.08]	[−0.01; 0.02]
Ideology: Right	−0.06*	−0.05*	0.12*	−0.10*	−0.08*	−0.04	−0.01	0.01	−0.02	−0.02	0.01
	[−0.10; −0.03]	[−0.09; −0.02]	[0.05; 0.20]	[−0.18; −0.03]	[−0.14; −0.02]	[−0.08; 0.01]	[−0.05; 0.04]	[−0.05; 0.07]	[−0.05; 0.01]	[−0.07; 0.02]	[−0.00; 0.03]
Hate experience: Yes	0.00	−0.00	0.04	−0.04	−0.05*	−0.01	−0.01	0.01	0.00	−0.01	0.00
	[−0.02; 0.03]	[−0.02; 0.02]	[−0.01; 0.10]	[−0.09; 0.01]	[−0.09; −0.01]	[−0.05; 0.02]	[−0.04; 0.02]	[−0.03; 0.05]	[−0.02; 0.03]	[−0.04; 0.02]	[−0.01; 0.01]
Hate witness: Yes	−0.00	−0.00	0.03	−0.01	0.03	0.02	−0.02	0.04*	−0.01	−0.03*	−0.00
	[−0.02; 0.02]	[−0.02; 0.02]	[−0.02; 0.07]	[−0.05; 0.04]	[−0.01; 0.07]	[−0.01; 0.05]	[−0.04; 0.01]	[0.01; 0.08]	[−0.02; 0.01]	[−0.05; −0.00]	[−0.01; 0.00]
Talk politics freely: Yes	−0.00	−0.01	−0.00	0.00	−0.03	−0.02	0.00	−0.01	−0.00	0.01	−0.00
	[−0.02; 0.02]	[−0.03; 0.01]	[−0.05; 0.04]	[−0.04; 0.04]	[−0.06; 0.01]	[−0.04; 0.01]	[−0.02; 0.02]	[−0.04; 0.03]	[−0.02; 0.02]	[−0.01; 0.04]	[−0.01; 0.01]
Party ID: Republican	−0.03	−0.03	0.06	−0.03	−0.02	−0.03	−0.03	0.05	−0.00	−0.01	−0.02*
	[−0.06; 0.01]	[−0.07; 0.00]	[−0.01; 0.14]	[−0.10; 0.04]	[−0.08; 0.04]	[−0.08; 0.02]	[−0.08; 0.01]	[−0.01; 0.11]	[−0.03; 0.03]	[−0.05; 0.03]	[−0.03; −0.00]
Party ID: Independent	−0.02	−0.02	0.09*	−0.07*	−0.01	−0.01	−0.03*	0.07*	−0.01	−0.03	−0.01*
	[−0.05; 0.01]	[−0.05; 0.00]	[0.04; 0.15]	[−0.13; −0.02]	[−0.06; 0.03]	[−0.05; 0.03]	[−0.07; −0.00]	[0.03; 0.11]	[−0.04; 0.01]	[−0.06; 0.00]	[−0.03; −0.00]
Num. obs.	7445	7391	7480	7480	7480	7480	7480	7480	7480	7480	7480
Num. groups: personid	935	935	935	935	935	935	935	935	935	935	935
Num. groups: deckid	370	370	370	370	370	370	370	370	370	370	370
Var: personid (Intercept)	0.01	0.01	0.08	0.08	0.05	0.03	0.02	0.05	0.01	0.03	0.00
Var: deckid (Intercept)	0.00	0.00	0.00	0.00	0.00	0.00	0.00	0.00	0.00	0.00	0.00
Var: Residual	0.05	0.05	0.16	0.14	0.11	0.09	0.07	0.08	0.03	0.05	0.01

* Null hypothesis value outside the confidence interval.

Table S20: Effects of respondent characteristics on hate speech perceptions and preferences for action (U.S. sample). Offensiveness and hatefulness scores were measured on five-point scales and rescaled to 0-1. Support for action was measured on a binary scale, 0-1. Linear mixed-effects models with person, vignette deck, and country random effects. 95% confidence intervals in parentheses.

	Hatefulness score	Platform sanctions	Other sanctions
Gender: Female	0.03*	0.08*	0.02
	[0.02; 0.04]	[0.05; 0.10]	[−0.01; 0.04]
Age: 30-49	−0.03*	−0.02	−0.01
	[−0.06; −0.01]	[−0.07; 0.03]	[−0.06; 0.03]
Age: 50-69	−0.03*	−0.02	−0.01
	[−0.06; −0.01]	[−0.08; 0.03]	[−0.06; 0.03]
Age: 70+	−0.00	−0.01	0.00
	[−0.03; 0.03]	[−0.07; 0.05]	[−0.05; 0.06]
Education: Intermediate	0.02	−0.02	−0.01
	[−0.00; 0.03]	[−0.06; 0.02]	[−0.04; 0.02]
Education: High	0.02	−0.03	−0.04*
	[−0.00; 0.04]	[−0.07; 0.00]	[−0.08; −0.01]
Political interest: Intermediate	−0.01	−0.02	−0.04
	[−0.03; 0.02]	[−0.06; 0.03]	[−0.08; 0.01]
Political interest: High	0.00	−0.03	−0.05*
	[−0.02; 0.02]	[−0.07; 0.02]	[−0.09; −0.01]
Social media user: Yes	0.01	−0.00	−0.04*
	[−0.01; 0.02]	[−0.04; 0.03]	[−0.07; −0.01]
Ideology: Center	−0.02*	−0.01	0.02
	[−0.03; −0.01]	[−0.04; 0.02]	[−0.01; 0.04]
Ideology: Right	−0.06*	−0.13*	−0.03*
	[−0.08; −0.05]	[−0.17; −0.10]	[−0.06; −0.00]
Hate experience: Yes	−0.00	−0.04*	0.02
	[−0.02; 0.02]	[−0.08; −0.01]	[−0.02; 0.05]
Hate witness: Yes	0.00	−0.01	−0.02
	[−0.01; 0.02]	[−0.04; 0.02]	[−0.04; 0.01]
Talk politics freely: Yes	−0.01	−0.01	−0.02
	[−0.02; 0.00]	[−0.03; 0.02]	[−0.04; 0.00]
Num. obs.	17343	17656	17656
Num. groups: personid	2204	2207	2207
Num. groups: deckid	747	747	747
Num. groups: country	2	2	2
Var: personid (Intercept)	0.01	0.06	0.05
Var: deckid (Intercept)	0.00	0.00	0.00
Var: country (Intercept)	0.00	0.00	0.01
Var: Residual	0.05	0.17	0.10

* Null hypothesis value outside the confidence interval.

Table S21: Effects of respondent characteristics on hate speech perceptions and preferences for action (pooled sample). Perceived hatefulness is re-scaled to a 0-100 scale scale (originally 5-point scale). The choice of platform sanctions is encoded as binary decision that takes the value 1 if the respondent chose at least one of the following options: delete post, block sender, temporarily ban sender, permanently ban sender. The choice of other sanctions is encoded as binary decision that takes the value 1 if the respondent chose at least one of the following options: lose job, pay fine, face prison sentence. Linear mixed-effects models with person, vignette deck, and country random effects. 95% confidence intervals in parentheses.

	Hatefulness score	Platform sanctions	Other sanctions
Gender: Female	0.04*	0.07*	0.02
	[0.02; 0.05]	[0.04; 0.11]	[−0.02; 0.05]
Age: 30-49	−0.03	0.01	0.01
	[−0.06; 0.00]	[−0.05; 0.07]	[−0.05; 0.07]
Age: 50-69	−0.04*	0.01	−0.00
	[−0.07; −0.00]	[−0.05; 0.07]	[−0.07; 0.06]
Age: 70+	−0.01	0.08	0.03
	[−0.05; 0.04]	[−0.01; 0.16]	[−0.06; 0.12]
Education: Intermediate	0.03	−0.01	0.01
	[−0.00; 0.05]	[−0.06; 0.04]	[−0.05; 0.06]
Education: High	0.02	−0.03	−0.03
	[−0.00; 0.05]	[−0.08; 0.02]	[−0.09; 0.02]
Political interest: Intermediate	−0.00	−0.02	−0.06
	[−0.04; 0.03]	[−0.09; 0.05]	[−0.14; 0.01]
Political interest: High	0.00	−0.04	−0.08*
	[−0.03; 0.04]	[−0.11; 0.03]	[−0.15; −0.01]
Social media user: Yes	0.00	−0.00	−0.04
	[−0.02; 0.02]	[−0.04; 0.04]	[−0.08; 0.00]
Ideology: Center	−0.00	0.02	0.03
	[−0.02; 0.02]	[−0.03; 0.06]	[−0.01; 0.07]
Ideology: Right	−0.02	−0.04	0.01
	[−0.06; 0.01]	[−0.10; 0.02]	[−0.05; 0.07]
Hate experience: Yes	0.01	−0.02	0.06*
	[−0.02; 0.04]	[−0.08; 0.04]	[0.00; 0.12]
Hate witness: Yes	0.00	0.01	0.02
	[−0.02; 0.02]	[−0.03; 0.05]	[−0.02; 0.06]
Talk politics freely: Yes	−0.01	−0.03	−0.05*
	[−0.03; 0.01]	[−0.07; 0.01]	[−0.09; −0.01]
Party ID: SPD	−0.00	0.00	0.02
	[−0.03; 0.03]	[−0.05; 0.06]	[−0.04; 0.08]
Party ID: FDP	−0.01	0.01	−0.02
	[−0.04; 0.02]	[−0.05; 0.08]	[−0.08; 0.05]
Party ID: Greens	0.01	0.03	0.01
	[−0.02; 0.04]	[−0.03; 0.08]	[−0.05; 0.07]
Party ID: Left	0.01	−0.01	0.01
	[−0.02; 0.04]	[−0.07; 0.05]	[−0.05; 0.07]
Party ID: AfD	−0.04*	−0.12*	−0.06*
	[−0.07; −0.01]	[−0.18; −0.07]	[−0.12; −0.00]
Party ID: Others	−0.00	−0.09*	−0.11*
	[−0.05; 0.04]	[−0.18; −0.01]	[−0.20; −0.01]
Num. obs.	8260	8432	8432
Num. groups: personid	1051	1054	1054
Num. groups: deckid	364	364	364
Var: personid (Intercept)	0.01	0.05	0.06
Var: deckid (Intercept)	0.00	0.00	0.00
Var: Residual	0.06	0.18	0.12

* Null hypothesis value outside the confidence interval.

Table S22: Effects of respondent characteristics on hate speech perceptions and preferences for action (German sample). Perceived hatefulness is re-scaled to a 0-100 scale scale (originally 5-point scale). The choice of platform sanctions is encoded as binary decision that takes the value 1 if the respondent chose at least one of the following options: delete post, block sender, temporarily ban sender, permanently ban sender. The choice of other sanctions is encoded as binary decision that takes the value 1 if the respondent chose at least one of the following options: lose job, pay fine, face prison sentence. Linear mixed-effects models with person, vignette deck, and country random effects. 95% confidence intervals in parentheses.

	Hatefulness score	Platform sanctions	Other sanctions
Gender: Female	0.02*	0.07*	0.02
	[0.00; 0.04]	[0.02; 0.11]	[−0.01; 0.05]
Age: 30-49	−0.03	−0.03	−0.04
	[−0.08; 0.02]	[−0.15; 0.08]	[−0.12; 0.04]
Age: 50-69	−0.01	−0.03	−0.04
	[−0.06; 0.03]	[−0.13; 0.08]	[−0.12; 0.04]
Age: 70+	0.02	−0.04	−0.03
	[−0.03; 0.07]	[−0.15; 0.08]	[−0.11; 0.05]
Education: Intermediate	0.01	−0.04	−0.01
	[−0.02; 0.04]	[−0.10; 0.03]	[−0.05; 0.04]
Education: High	0.01	−0.05	−0.05*
	[−0.02; 0.04]	[−0.11; 0.01]	[−0.09; −0.00]
Political interest: Intermediate	−0.02	−0.02	−0.03
	[−0.06; 0.02]	[−0.10; 0.07]	[−0.09; 0.03]
Political interest: High	0.00	−0.02	−0.05*
	[−0.03; 0.03]	[−0.09; 0.05]	[−0.10; −0.00]
Social media user: Yes	0.03*	0.01	−0.04
	[0.00; 0.06]	[−0.06; 0.08]	[−0.09; 0.01]
Ideology: Center	−0.02	0.02	0.05*
	[−0.04; 0.01]	[−0.04; 0.08]	[0.01; 0.09]
Ideology: Right	−0.05*	−0.13*	−0.03
	[−0.09; −0.02]	[−0.20; −0.05]	[−0.08; 0.03]
Hate experience: Yes	−0.00	−0.04	0.00
	[−0.02; 0.02]	[−0.09; 0.01]	[−0.04; 0.04]
Hate witness: Yes	−0.00	−0.03	−0.05*
	[−0.02; 0.02]	[−0.07; 0.02]	[−0.08; −0.01]
Talk politics freely: Yes	−0.01	0.00	0.01
	[−0.03; 0.01]	[−0.04; 0.04]	[−0.02; 0.04]
Party ID: Republican	−0.03	−0.06	−0.02
	[−0.07; 0.00]	[−0.13; 0.02]	[−0.08; 0.03]
Party ID: Independent	−0.02	−0.09*	−0.04*
	[−0.05; 0.00]	[−0.14; −0.03]	[−0.08; −0.00]
Num. obs.	7391	7480	7480
Num. groups: personid	935	935	935
Num. groups: deckid	370	370	370
Var: personid (Intercept)	0.01	0.08	0.04
Var: deckid (Intercept)	0.00	0.00	0.00
Var: Residual	0.05	0.16	0.07

* Null hypothesis value outside the confidence interval.

Table S23: Effects of respondent characteristics on hate speech perceptions and preferences for action (U.S. sample). Perceived hatefulness is re-scaled to a 0-100 scale scale (originally 5-point scale). The choice of platform sanctions is encoded as binary decision that takes the value 1 if the respondent chose at least one of the following options: delete post, block sender, temporarily ban sender, permanently ban sender. The choice of other sanctions is encoded as binary decision that takes the value 1 if the respondent chose at least one of the following options: lose job, pay fine, face prison sentence. Linear mixed-effects models with person, vignette deck, and country random effects. 95% confidence intervals in parentheses.

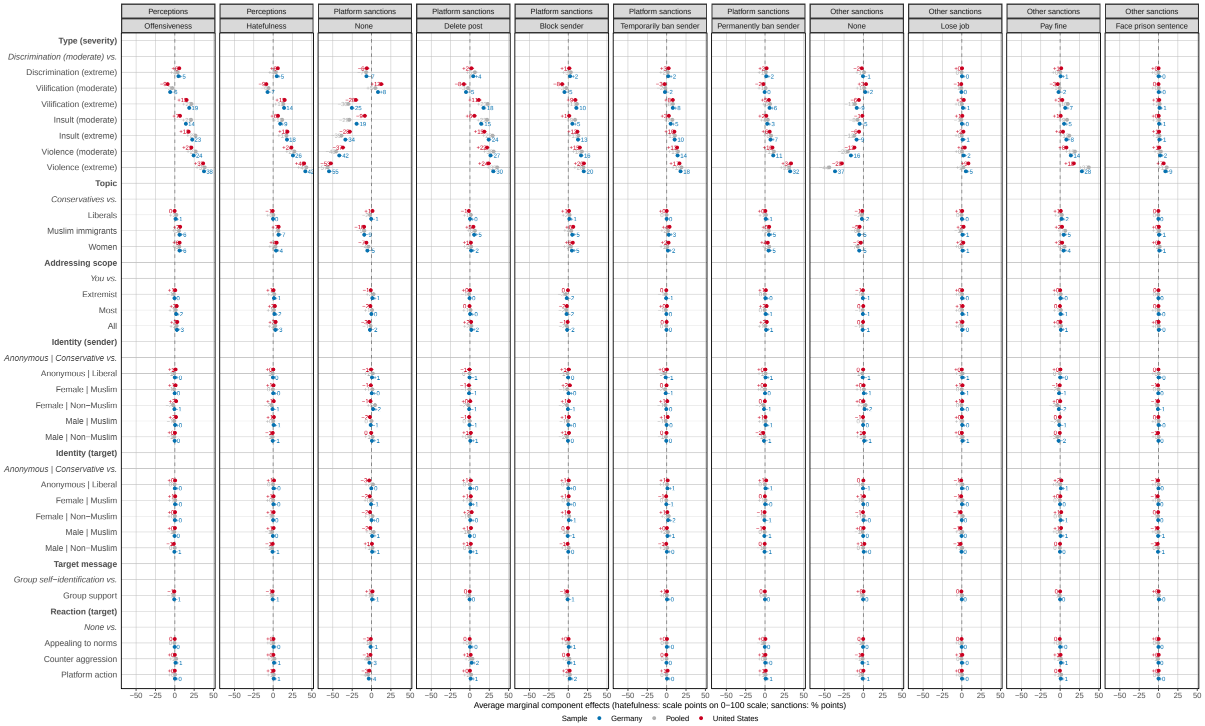

Figure S17: Estimated average marginal component effects of content and context characteristics of social media hate speech vignettes on citizens' perceptions and preferred platform-side and other action (by country). Perceived hatefulness and offensiveness are measured on 5-point scales (re-scaled to 0-1 scale), preferences for platform action and further penalties are binary measures. Error bars represent 95% confidence intervals.

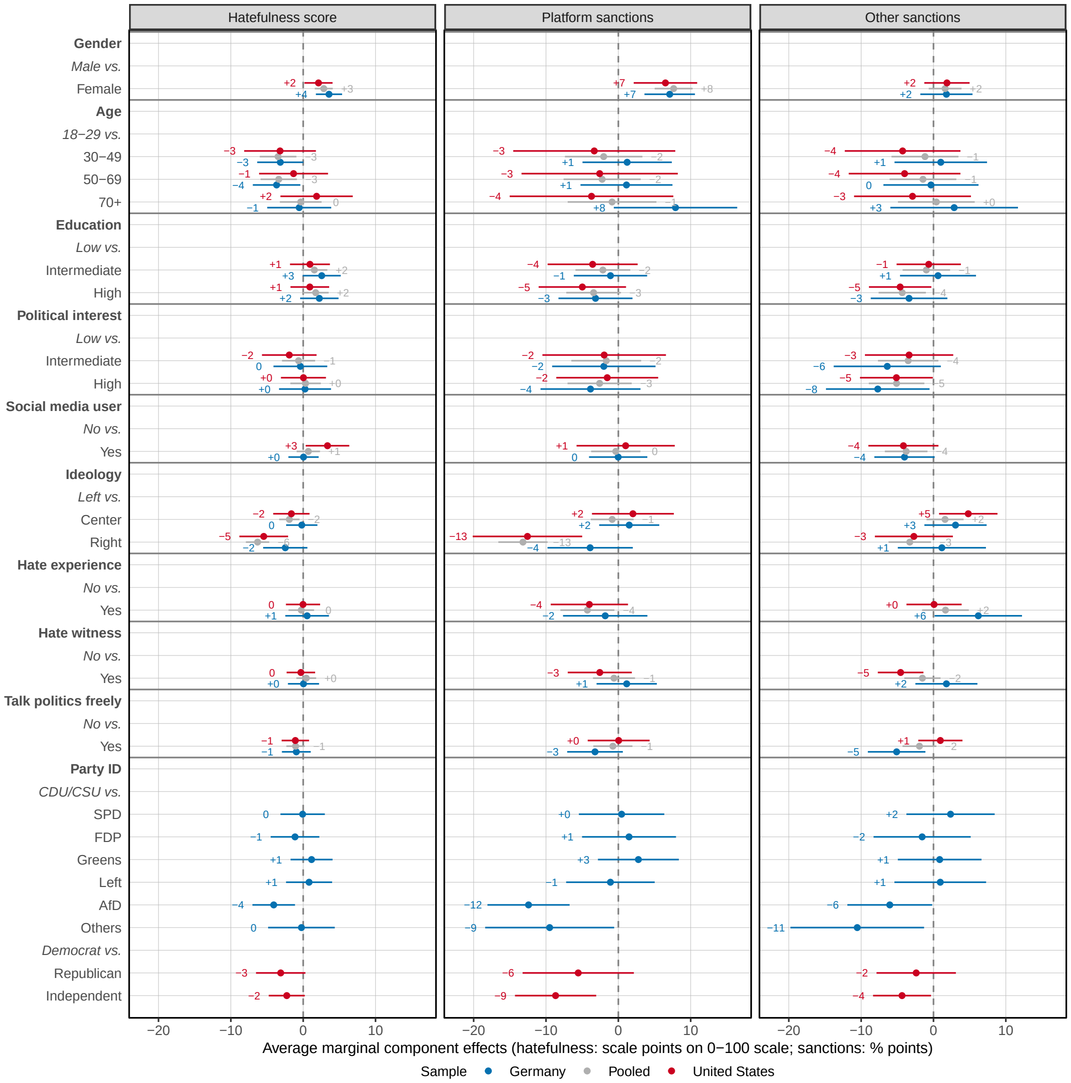

Figure S18: Estimated effects of respondent characteristics on citizens' perceptions and preferred platform-side and other action, by country. Estimates from hierarchical linear models with person, vignette deck, and country random effects. Perceived hatefulness is re-scaled to a 0-100 scale (originally 5-point scale). The choice of platform sanctions is encoded as a binary decision that takes the value 1 if the respondent chose at least one of the following options: delete post, block sender, temporarily ban sender, permanently ban sender. The choice of other sanctions is encoded as a binary decision that takes the value 1 if the respondent chose at least one of the following options: lose job, pay fine, face prison sentence. Error bars represent 95% confidence intervals. See Supplementary Table S18 for detailed regression results.

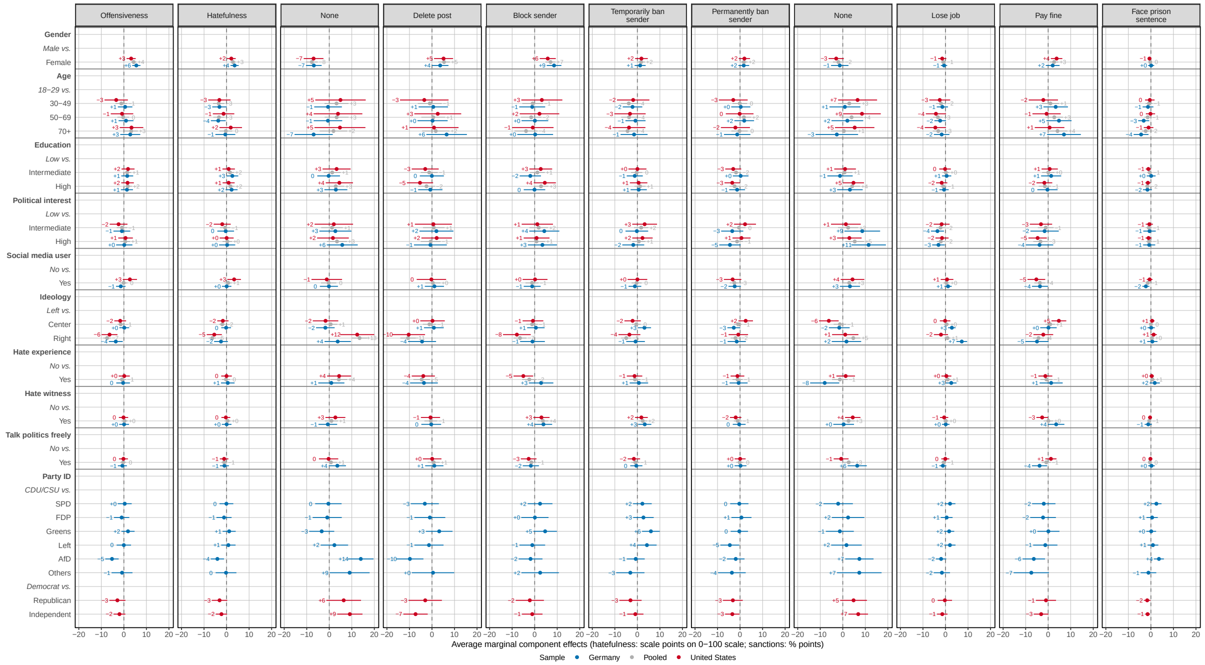

Figure S19: Estimated effects of respondent characteristics on citizens' perceptions and preferred platform-side and other action (by country). Perceived hatefulness and offensiveness are measured on 5-point scales (re-scaled to 0-1 scale), preferences for platform action and further penalties are binary measures. Error bars represent 95% confidence intervals.

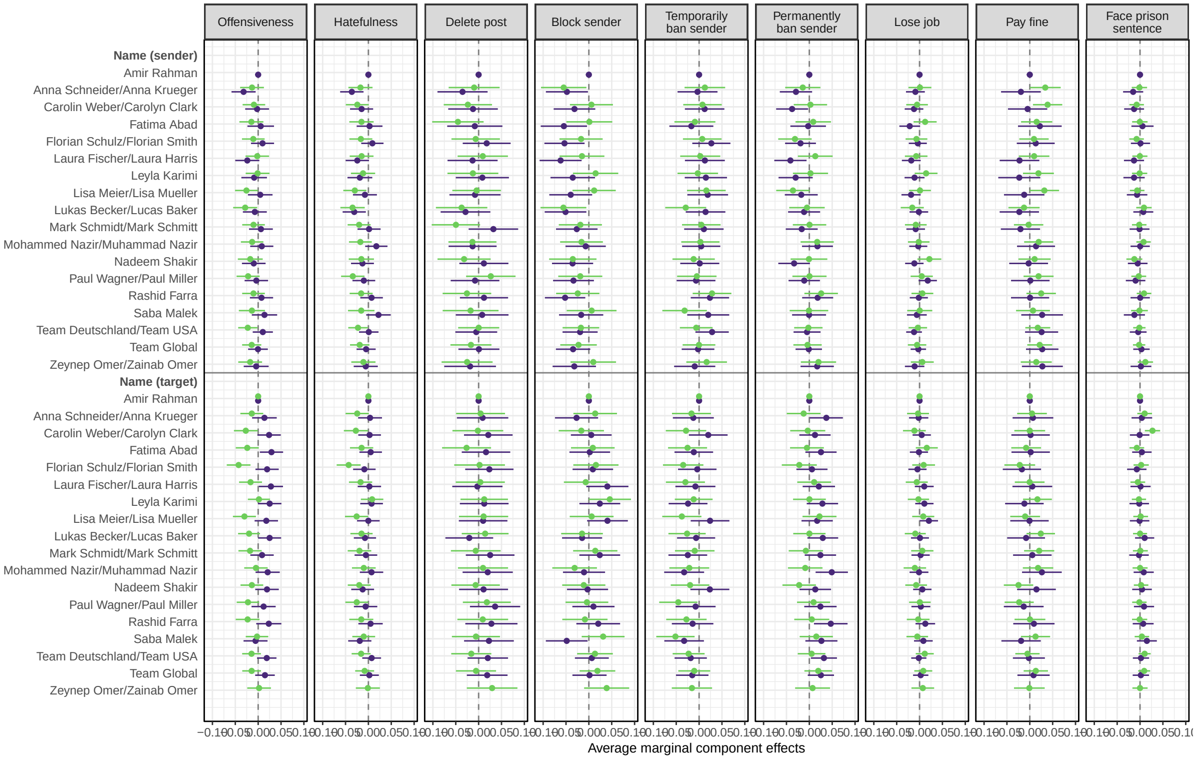

Figure S20: Estimated average marginal component effects of sender/target names (and associated profile pictures) on citizens' perceptions and preferred platform-side and other action (by country). Perceived hatefulness and offensiveness are measured on 5-point scales (re-scaled to 0-1 scale), preferences for platform action and further penalties are binary measures. Error bars represent 95% confidence intervals.

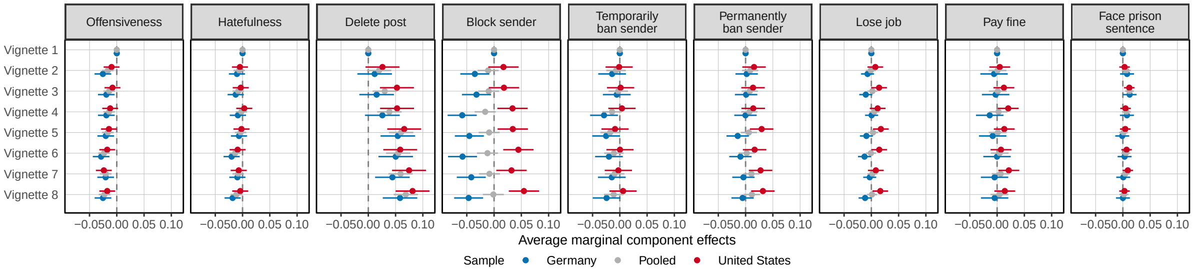

Figure S21: Estimated effect of vignette position (1 to 8) on citizens' perceptions and preferred platform-side and other action (pooled). Perceived hatefulness and offensiveness are measured on 5-point scales (re-scaled to 0-1 scale), preferences for platform action and further penalties are binary measures. Error bars represent 95% confidence intervals.

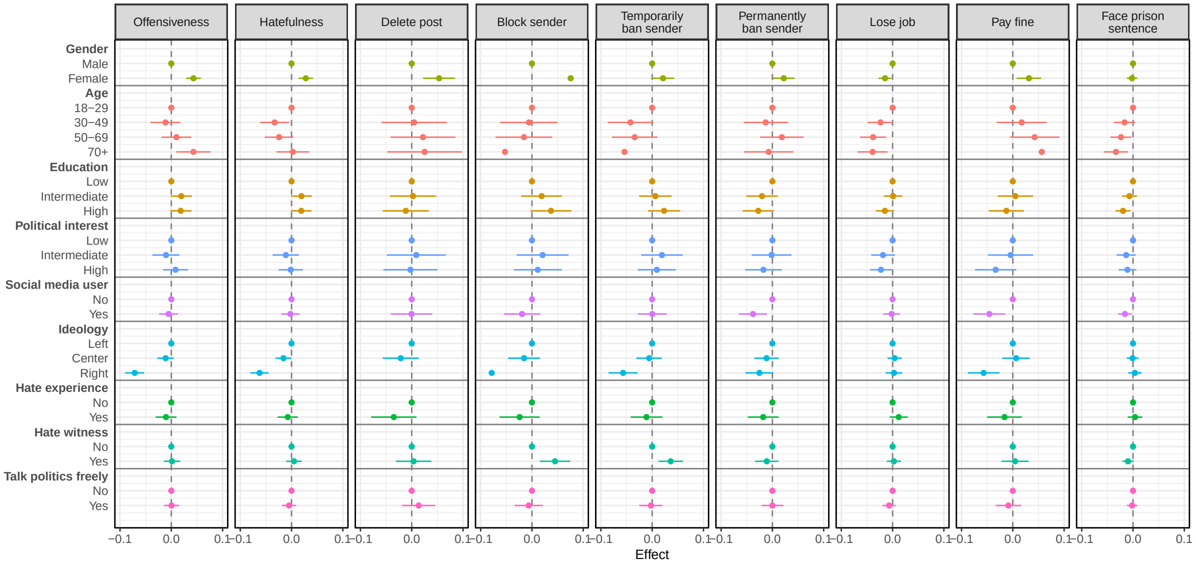

Figure S22: Estimated effects of respondent characteristics on citizens' perceptions and preferred platform-side and other action (by country; restricted to violent and insulting messages). Perceived hatefulness and offensiveness are measured on 5-point scales (re-scaled to 0-1 scale), preferences for platform action and further penalties are binary measures. Error bars represent 95% confidence intervals.

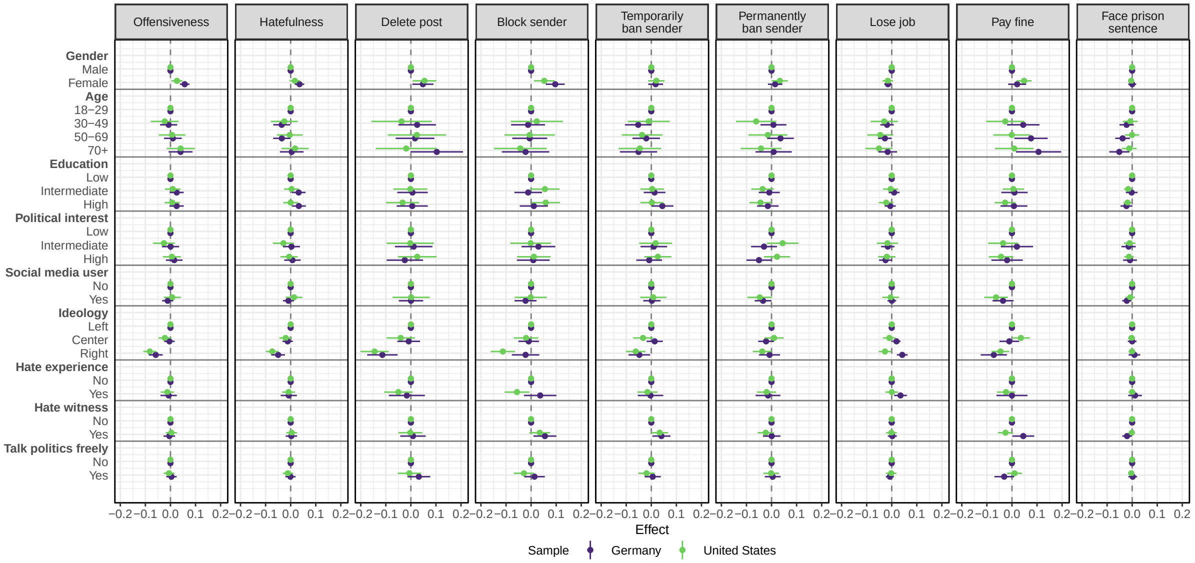

Figure S23: Estimated effects of respondent characteristics on citizens' perceptions and preferred platform-side and other action (by country; restricted to violent and insulting messages). Perceived hatefulness and offensiveness are measured on 5-point scales (re-scaled to 0-1 scale), preferences for platform action and further penalties are binary measures. Error bars represent 95% confidence intervals.

Attribute effect	Outcome: Hatefulness score	Provider action	Other action
Sender category score: Discrimination (extreme)	53	22	−9
Sender category score: Vilification (moderate)	140	42	−5
Sender category score: Vilification (extreme)	586	488	71
Sender category score: Insult (moderate)	288	332	28
Sender category score: Insult (extreme)	1,125	1,018	99
Sender category score: Violence (moderate)	2,229	1,534	350
Sender category score: Violence (extreme)	5,358	2,569	1,701
Topic: Liberals	−10	−9	−1
Topic: Muslim immigrants	301	140	62
Topic: Women	81	39	61
Target group category: Extremist	−3	−7	−8
Target group category: Most	7	−10	−9
Target group category: All	45	−4	−4
Identity sender: Anonymous | Liberal	−10	−9	−9
Identity sender: Female | Muslim	−10	−10	−9
Identity sender: Female | Non-Muslim	−7	−4	−5
Identity sender: Male | Muslim	−9	−10	−10
Identity sender: Male | Non-Muslim	−8	−9	−9
Identity target: Anonymous | Liberal	−9	−10	−9
Identity target: Female | Muslim	−10	−9	−10
Identity target: Female | Non-Muslim	−10	−10	−10
Identity target: Male | Muslim	−9	−9	−10
Identity target: Male | Non-Muslim	−7	−9	−7
Target message: Group support	−3	−7	−10
Target reply category: Appealing to norms	−8	−7	−10
Target reply category: Counter aggression	−1	8	−5
Target reply category: Platform action	−6	17	−7

Color legend: Evidence based on 2*log(Bayes Factor) (Raftery 1995)

weakly in favor of 0 | positively in favor of 0 | strongly in favor of 0 | very strongly in favor of 0

weakly against 0 | positively against 0 | strongly against 0 | very strongly against 0

Figure S24: Bayes factors ($2log$) against the Null for content and context attributes in the vignettes study (Study 1, pooled sample). The table reports twice the natural logarithm of the Bayes factor and uses Kass and Raftery's scale for interpretation of evidence for/against the Null, with scale $\{]\text{-Inf}, -10],]-10, -6],]-6, -2],]-2, 0], [0, 2[, [2, 6[, [6, 10[, [10, \text{Inf}[\}$ ranging from "very strongly in favor of the Null" to "very strongly against the Null". Bayes factors are computed by assessing the pairwise ratio of marginal likelihoods for two models, one of which is specified with the predictor of interest and the other one without (which is the baseline model).

Attribute effect	Outcome: Hatefulness score	Outcome: Provider action	Outcome: Other action
Sender category score: Discrimination (extreme)	8	10	−9
Sender category score: Vilification (moderate)	40	−1	−9
Sender category score: Vilification (extreme)	237	340	47
Sender category score: Insult (moderate)	223	372	32
Sender category score: Insult (extreme)	495	684	75
Sender category score: Violence (moderate)	1,216	933	235
Sender category score: Violence (extreme)	2,768	1,405	1,115
Topic: Liberals	−9	−6	−6
Topic: Muslim immigrants	132	65	34
Topic: Women	19	2	39
Target group category: Extremist	−2	−2	−9
Target group category: Most	−6	−9	−9
Target group category: All	14	−9	−6
Identity sender: Anonymous | Liberal	−9	−7	−9
Identity sender: Female | Muslim	−9	−8	−8
Identity sender: Female | Non-Muslim	−1	5	0
Identity sender: Male | Muslim	−9	−9	−9
Identity sender: Male | Non-Muslim	−8	−8	−8
Identity target: Anonymous | Liberal	−9	−8	−9
Identity target: Female | Muslim	−9	−9	−9
Identity target: Female | Non-Muslim	−8	−9	−9
Identity target: Male | Muslim	−6	−4	−9
Identity target: Male | Non-Muslim	−6	−9	−9
Target message: Group support	−6	−8	−9
Target reply category: Appealing to norms	−9	−8	−9
Target reply category: Counter aggression	3	11	−8
Target reply category: Platform action	−6	12	−5

Color legend: Evidence based on 2*log(Bayes Factor) (Raftery 1995)

weakly in favor of 0 | positively in favor of 0 | strongly in favor of 0 | very strongly in favor of 0

weakly against 0 | positively against 0 | strongly against 0 | very strongly against 0

Figure S25: Bayes factors ($2log$) against the Null for content and context attributes in the vignettes study (Study 1, German sample). The table reports twice the natural logarithm of the Bayes factor and uses Kass and Raftery's scale for interpretation of evidence for/against the Null, with scale $\{]\text{-Inf}, -10],]-10, -6],]-6, -2],]-2, 0], [0, 2[, [2, 6[, [6, 10[, [10, \text{Inf}[\}$ ranging from "very strongly in favor of the Null" to "very strongly against the Null". Bayes factors are computed by assessing the pairwise ratio of marginal likelihoods for two models, one of which is specified with the predictor of interest and the other one without (which is the baseline model).

Attribute effect	Outcome: Hatefulness score	Outcome: Provider action	Outcome: Other action
Sender category score: Discrimination (extreme)	47	4	−7
Sender category score: Vilification (moderate)	107	52	0
Sender category score: Vilification (extreme)	359	148	14
Sender category score: Insult (moderate)	67	24	−7
Sender category score: Insult (extreme)	650	343	17
Sender category score: Violence (moderate)	1,023	602	108
Sender category score: Violence (extreme)	2,618	1,162	572
Topic: Liberals	−8	−8	−5
Topic: Muslim immigrants	167	69	24
Topic: Women	63	33	12
Target group category: Extremist	−8	−9	−8
Target group category: Most	9	−8	−9
Target group category: All	28	−1	−6
Identity sender: Anonymous | Liberal	−9	−9	−9
Identity sender: Female | Muslim	−9	−8	−9
Identity sender: Female | Non-Muslim	−8	−8	−9
Identity sender: Male | Muslim	−7	−7	−9
Identity sender: Male | Non-Muslim	−8	−9	−9
Identity target: Anonymous | Liberal	−7	−6	−9
Identity target: Female | Muslim	−8	−7	−8
Identity target: Female | Non-Muslim	−8	−8	−9
Identity target: Male | Muslim	−8	−8	−9
Identity target: Male | Non-Muslim	−8	−9	−5
Target message: Group support	−5	−6	−9
Target reply category: Appealing to norms	−8	−8	−9
Target reply category: Counter aggression	−9	−7	−7
Target reply category: Platform action	−8	−1	−9

Color legend: Evidence based on 2*log(Bayes Factor) (Raftery 1995)

weakly in favor of 0 | positively in favor of 0 | strongly in favor of 0 | very strongly in favor of 0

weakly against 0 | positively against 0 | strongly against 0 | very strongly against 0

Figure S26: Bayes factors ($2log$) against the Null for content and context attributes in the vignettes study (Study 1, U.S. sample). The table reports twice the natural logarithm of the Bayes factor and uses Kass and Raftery's scale for interpretation of evidence for/against the Null, with scale $\{]\text{-Inf}, -10],]-10, -6],]-6, -2],]-2, 0], [0, 2[, [2, 6[, [6, 10[, [10, \text{Inf}[\}$ ranging from "very strongly in favor of the Null" to "very strongly against the Null". Bayes factors are computed by assessing the pairwise ratio of marginal likelihoods for two models, one of which is specified with the predictor of interest and the other one without (which is the baseline model).

	Outcome		
Respondent covariate	Hatefulness score	Provider action	Other action
Female: Female	11	23	−8
Age: 30-49	−3	−9	−10
Age: 50-69	−4	−9	−9
Age: 70+	−10	−10	−10
Educ: Intermediate	−7	−9	−9
Educ: High	−6	−7	−3
Polinterest: Intermediate	−9	−9	−7
Polinterest: High	−10	−8	−3
Socialmedia accounts: Yes	−9	−10	−3
Ideology: Center	−2	−9	−8
Ideology: Right	46	48	−5
Hate experience: Yes	−10	−5	−9
Hate witness: Yes	−9	−10	−8
Discuss politics free: Yes	−7	−10	−7

Color legend: Evidence based on 2*log(Bayes Factor) (Raftery 1995)

weakly in favor of 0	positively in favor of 0	strongly in favor of 0	very strongly in favor of 0
weakly against 0	positively against 0	strongly against 0	very strongly against 0

Figure S27: Bayes factors ($2log$) against the Null for respondent characteristics in the vignettes study (Study 1, pooled sample). The table reports twice the natural logarithm of the Bayes factor and uses Kass and Raftery's scale for interpretation of evidence for/against the Null, with scale $\{]\text{-Inf}, -10],]-10, -6],]-6, -2],]-2, 0], [0, 2[, [2, 6[, [6, 10[, [10, \text{Inf}[\}$ ranging from "very strongly in favor of the Null" to "very strongly against the Null". Bayes factors are computed by assessing the pairwise ratio of marginal likelihoods for two models, one of which is specified with the predictor of interest and the other one without (which is the baseline model).

Respondent covariate	Outcome		
	Hatefulness score	Provider action	Other action
Female: Female	6	7	−8
Age: 30-49	−5	−9	−9
Age: 50-69	−4	−9	−9
Age: 70+	−9	−6	−9
Educ: Intermediate	−5	−9	−9
Educ: High	−6	−8	−7
Polinterest: Intermediate	−9	−9	−6
Polinterest: High	−9	−8	−5
Socialmedia accounts: Yes	−9	−9	−5
Ideology: Center	−9	−9	−7
Ideology: Right	−6	−7	−9
Hate experience: Yes	−9	−9	−5
Hate witness: Yes	−9	−9	−8
Discuss politics free: Yes	−8	−6	−3
Party id: SPD	−9	−9	−8
Party id: FDP	−9	−9	−9
Party id: Greens	−8	−8	−9
Party id: Left	−9	−9	−9
Party id: AfD	−2	10	−5
Party id: Others	−9	−5	−4
Party id: DK	0	0	0

Color legend: Evidence based on 2*log(Bayes Factor) (Raftery 1995)

weakly in favor of 0	positively in favor of 0	strongly in favor of 0	very strongly in favor of 0
weakly against 0	positively against 0	strongly against 0	very strongly against 0

Figure S28: Bayes factors ($2log$) against the Null for respondent characteristics in the vignettes study (Study 1, German sample). The table reports twice the natural logarithm of the Bayes factor and uses Kass and Raftery's scale for interpretation of evidence for/against the Null, with scale $\{]\text{-Inf}, -10],]-10, -6],]-6, -2],]-2, 0], [0, 2[, [2, 6[, [6, 10[, [10, \text{Inf}[\}$ ranging from "very strongly in favor of the Null" to "very strongly against the Null". Bayes factors are computed by assessing the pairwise ratio of marginal likelihoods for two models, one of which is specified with the predictor of interest and the other one without (which is the baseline model).

Respondent covariate	Outcome		
	Hatefulness score	Provider action	Other action
Female: Female	−4	0	−8
Age: 30-49	−7	−9	−8
Age: 50-69	−9	−9	−8
Age: 70+	−8	−9	−8
Educ: Intermediate	−8	−8	−9
Educ: High	−8	−6	−4
Polinterest: Intermediate	−8	−9	−8
Polinterest: High	−9	−9	−5
Socialmedia accounts: Yes	−4	−9	−6
Ideology: Center	−7	−8	−3
Ideology: Right	1	2	−8
Hate experience: Yes	−9	−7	−9
Hate witness: Yes	−9	−8	−1
Discuss politics free: Yes	−8	−9	−9
Party id: Republican	−6	−7	−8
Party id: Independent	−6	0	−4

Color legend: Evidence based on 2*log(Bayes Factor) (Raftery 1995)

weakly in favor of 0	positively in favor of 0	strongly in favor of 0	very strongly in favor of 0
weakly against 0	positively against 0	strongly against 0	very strongly against 0

Figure S29: Bayes factors ($2log$) against the Null for respondent characteristics in the vignettes study (Study 1, U.S. sample). The table reports twice the natural logarithm of the Bayes factor and uses Kass and Raftery's scale for interpretation of evidence for/against the Null, with scale $\{]\text{-Inf}, -10],]-10, -6],]-6, -2],]-2, 0], [0, 2[, [2, 6[, [6, 10[, [10, \text{Inf}[\}$ ranging from "very strongly in favor of the Null" to "very strongly against the Null". Bayes factors are computed by assessing the pairwise ratio of marginal likelihoods for two models, one of which is specified with the predictor of interest and the other one without (which is the baseline model).

D.3 Vignette evaluations, interaction effects

	Hatefulness score	Platform sanctions	Other sanctions
Gender (respondent): Female	0.03*	0.09*	0.03*
	[0.02; 0.04]	[0.06; 0.12]	[0.00; 0.05]
Gender (target): Female	0.01	0.02	−0.00
	[−0.00; 0.02]	[−0.00; 0.03]	[−0.02; 0.01]
Gender (sender): Female	−0.01*	−0.03*	−0.02*
	[−0.02; −0.00]	[−0.04; −0.01]	[−0.03; −0.00]
Gender (respondent) * Gender (target): Female * Female	0.01	0.01	0.02
	[−0.00; 0.02]	[−0.02; 0.03]	[−0.00; 0.04]
Gender (respondent) * Gender (sender): Female * Female	0.00	0.01	−0.01
	[−0.01; 0.02]	[−0.01; 0.04]	[−0.03; 0.02]
Num. obs.	11583	11811	11811
Num. groups: personid	2613	2622	2622
Num. groups: deckid	768	768	768
Num. groups: country	2	2	2
Var: personid (Intercept)	0.01	0.07	0.06
Var: deckid (Intercept)	0.00	0.00	0.00
Var: country (Intercept)	0.00	0.00	0.01
Var: Residual	0.03	0.13	0.08

* Null hypothesis value outside the confidence interval.

Table S24: Effects of respondent gender X target/sender gender on hate speech perceptions and preferences for action (pooled sample). Perceived hatefulness is rescaled to a 0-100 scale scale (originally 5-point scale). The choice of platform sanctions is encoded as binary decision that takes the value 1 if the respondent chose at least one of the following options: delete post, block sender, temporarily ban sender, permanently ban sender. The choice of other sanctions is encoded as binary decision that takes the value 1 if the respondent chose at least one of the following options: lose job, pay fine, face prison sentence. Linear mixed-effects models with person, vignette deck, and country random effects. 95% confidence intervals in parentheses.

	Hatefulness score	Platform sanctions	Other sanctions
Gender (respondent): Female	0.03*	0.09*	0.01
	[0.01; 0.05]	[0.05; 0.13]	[−0.03; 0.05]
Gender (target): Female	0.00	0.02	−0.02
	[−0.01; 0.02]	[−0.01; 0.04]	[−0.04; 0.01]
Gender (sender): Female	−0.01	−0.02	−0.02*
	[−0.02; 0.01]	[−0.04; 0.01]	[−0.05; −0.00]
Gender (respondent) * Gender (target): Female * Female	0.01	0.01	0.04*
	[−0.01; 0.03]	[−0.03; 0.05]	[0.01; 0.07]
Gender (respondent) * Gender (sender): Female * Female	0.00	0.01	−0.01
	[−0.02; 0.02]	[−0.03; 0.05]	[−0.04; 0.03]
Num. obs.	6158	6310	6310
Num. groups: personid	1382	1390	1390
Num. groups: deckid	381	381	381
Var: personid (Intercept)	0.01	0.05	0.07
Var: deckid (Intercept)	0.00	0.00	0.00
Var: Residual	0.03	0.13	0.10

* Null hypothesis value outside the confidence interval.

Table S25: Effects of respondent gender X target/sender gender on hate speech perceptions and preferences for action (German sample). Perceived hatefulness is rescaled to a 0-100 scale scale (originally 5-point scale). The choice of platform sanctions is encoded as binary decision that takes the value 1 if the respondent chose at least one of the following options: delete post, block sender, temporarily ban sender, permanently ban sender. The choice of other sanctions is encoded as binary decision that takes the value 1 if the respondent chose at least one of the following options: lose job, pay fine, face prison sentence. Linear mixed-effects models with person, vignette deck, and country random effects. 95% confidence intervals in parentheses.

	Hatefulness score	Platform sanctions	Other sanctions
Gender (respondent): Female	0.03^{*}	0.10^{*}	0.05^{*}
	$[0.00; 0.05]$	$[0.05; 0.14]$	$[0.02; 0.08]$
Gender (target): Female	0.01	0.02	0.01
	$[-0.00; 0.02]$	$[-0.01; 0.05]$	$[-0.01; 0.03]$
Gender (sender): Female	-0.01	-0.03^{*}	-0.01
	$[-0.03; 0.00]$	$[-0.06; -0.00]$	$[-0.03; 0.01]$
Gender (respondent) * Gender (target): Female * Female	0.01	0.01	-0.00
	$[-0.01; 0.03]$	$[-0.03; 0.04]$	$[-0.03; 0.03]$
Gender (respondent) * Gender (sender): Female * Female	0.00	0.01	-0.01
	$[-0.02; 0.02]$	$[-0.03; 0.05]$	$[-0.03; 0.02]$
Num. obs.	5425	5501	5501
Num. groups: personid	1231	1232	1232
Num. groups: deckid	387	387	387
Var: personid (Intercept)	0.02	0.09	0.05
Var: deckid (Intercept)	0.00	0.00	0.00
Var: Residual	0.03	0.12	0.06

* Null hypothesis value outside the confidence interval.

Table S26: Effects of respondent gender X target/sender gender on hate speech perceptions and preferences for action (U.S. sample). Perceived hatefulness is re-scaled to a 0-100 scale scale (originally 5-point scale). The choice of platform sanctions is encoded as binary decision that takes the value 1 if the respondent chose at least one of the following options: delete post, block sender, temporarily ban sender, permanently ban sender. The choice of other sanctions is encoded as binary decision that takes the value 1 if the respondent chose at least one of the following options: lose job, pay fine, face prison sentence. Linear mixed-effects models with person, vignette deck, and country random effects. 95% confidence intervals in parentheses.

	Hatefulness score	Platform sanctions	Other sanctions
Ideology (respondent): Right	-0.02	-0.12^{*}	-0.02
	$[-0.07; 0.02]$	$[-0.22; -0.03]$	$[-0.09; 0.06]$
Ideology (target): Liberal	0.04	0.09^{*}	0.01
	$[-0.00; 0.09]$	$[0.00; 0.18]$	$[-0.06; 0.09]$
Ideology (respondent) * Ideology (target): Right * Liberal	-0.10^{*}	-0.13	-0.03
	$[-0.16; -0.03]$	$[-0.26; 0.00]$	$[-0.14; 0.08]$
Num. obs.	706	718	718
Num. groups: personid	666	675	675
Num. groups: deckid	316	318	318
Num. groups: country	2	2	2
Var: personid (Intercept)	0.02	0.07	0.07
Var: deckid (Intercept)	0.00	0.01	0.00
Var: country (Intercept)	0.00	0.01	0.01
Var: Residual	0.02	0.11	0.06

* Null hypothesis value outside the confidence interval.

Table S27: Effects of respondent ideology X target/sender ideology on hate speech perceptions and preferences for action **(pooled sample).** Perceived hatefulness is re-scaled to a 0-100 scale scale (originally 5-point scale). The choice of platform sanctions is encoded as binary decision that takes the value 1 if the respondent chose at least one of the following options: delete post, block sender, temporarily ban sender, permanently ban sender. The choice of other sanctions is encoded as binary decision that takes the value 1 if the respondent chose at least one of the following options: lose job, pay fine, face prison sentence. Linear mixed-effects models with person, vignette deck, and country random effects. 95% confidence intervals in parentheses.

	Hatefulness score	Platform sanctions	Other sanctions
Ideology (respondent): Right	−0.00	−0.14	0.01
	[−0.08; 0.07]	[−0.28; 0.01]	[−0.13; 0.15]
Ideology (target): Liberal	0.01	0.04	0.01
	[−0.06; 0.09]	[−0.09; 0.17]	[−0.11; 0.13]
Ideology (respondent) * Ideology (target): Right * Liberal	−0.06	−0.05	−0.08
	[−0.16; 0.05]	[−0.25; 0.15]	[−0.26; 0.11]
Num. obs.	314	319	319
Num. groups: personid	289	293	293
Num. groups: deckid	156	157	157
Var: personid (Intercept)	0.03	0.07	0.09
Var: deckid (Intercept)	0.01	0.02	0.00
Var: Residual	0.02	0.10	0.08

* Null hypothesis value outside the confidence interval.

Table S28: Effects of respondent ideology X target/sender ideology on hate speech perceptions and preferences for action (German sample). Perceived hatefulness is re-scaled to a 0-100 scale scale (originally 5-point scale). The choice of platform sanctions is encoded as binary decision that takes the value 1 if the respondent chose at least one of the following options: delete post, block sender, temporarily ban sender, permanently ban sender. The choice of other sanctions is encoded as binary decision that takes the value 1 if the respondent chose at least one of the following options: lose job, pay fine, face prison sentence. Linear mixed-effects models with person, vignette deck, and country random effects. 95% confidence intervals in parentheses.

	Hatefulness score	Platform sanctions	Other sanctions
Ideology (respondent): Right	−0.04	−0.12	−0.04
	[−0.09; 0.02]	[−0.24; 0.01]	[−0.13; 0.06]
Ideology (target): Liberal	0.05	0.11	−0.01
	[−0.00; 0.11]	[−0.02; 0.23]	[−0.10; 0.08]
Ideology (respondent) * Ideology (target): Right * Liberal	-0.13^*	−0.17	0.01
	[−0.21; −0.05]	[−0.35; 0.00]	[−0.12; 0.14]
Num. obs.	392	399	399
Num. groups: personid	377	382	382
Num. groups: deckid	160	161	161
Var: personid (Intercept)	0.02	0.07	0.06
Var: deckid (Intercept)	0.00	0.01	0.00
Var: Residual	0.02	0.11	0.05

* Null hypothesis value outside the confidence interval.

Table S29: Effects of respondent ideology X target/sender ideology on hate speech perceptions and preferences for action (U.S. sample). Perceived hatefulness is re-scaled to a 0-100 scale scale (originally 5-point scale). The choice of platform sanctions is encoded as binary decision that takes the value 1 if the respondent chose at least one of the following options: delete post, block sender, temporarily ban sender, permanently ban sender. The choice of other sanctions is encoded as binary decision that takes the value 1 if the respondent chose at least one of the following options: lose job, pay fine, face prison sentence. Linear mixed-effects models with person, vignette deck, and country random effects. 95% confidence intervals in parentheses.

	Hatefulness score	Platform sanctions	Other sanctions
Topic: Liberals	-0.01^{*}	-0.01	0.02^{*}
	$[-0.02; -0.00]$	$[-0.02; 0.01]$	$[0.00; 0.03]$
Topic: Muslim immigrants	0.06^{*}	0.09^{*}	0.05^{*}
	$[0.05; 0.07]$	$[0.07; 0.11]$	$[0.03; 0.06]$
Topic: Women	0.02^{*}	0.04^{*}	0.04^{*}
	$[0.01; 0.03]$	$[0.02; 0.06]$	$[0.02; 0.05]$
Gender: Female	0.02^{*}	0.09^{*}	0.02
	$[0.01; 0.03]$	$[0.06; 0.12]$	$[-0.00; 0.05]$
Gender * Topic: Female * Liberals	0.02^{*}	0.02	-0.00
	$[0.00; 0.03]$	$[-0.00; 0.05]$	$[-0.02; 0.02]$
Gender * Topic: Female * Muslim immigrants	0.03^{*}	0.01	0.01
	$[0.01; 0.04]$	$[-0.02; 0.04]$	$[-0.01; 0.03]$
Gender * Topic: Female * Women	0.03^{*}	0.02	0.03^{*}
	$[0.01; 0.04]$	$[-0.00; 0.05]$	$[0.01; 0.06]$
Num. obs.	20589	20976	20976
Num. groups: personid	2617	2622	2622
Num. groups: deckid	768	768	768
Num. groups: country	2	2	2
Var: personid (Intercept)	0.02	0.07	0.06
Var: deckid (Intercept)	0.00	0.00	0.00
Var: country (Intercept)	0.00	0.00	0.01
Var: Residual	0.03	0.12	0.08

* Null hypothesis value outside the confidence interval.

Table S30: Effects of respondent gender X speech topic on hate speech perceptions and preferences for action (pooled sample)**.** Perceived hatefulness is re-scaled to a 0-100 scale scale (originally 5-point scale). The choice of platform sanctions is encoded as binary decision that takes the value 1 if the respondent chose at least one of the following options: delete post, block sender, temporarily ban sender, permanently ban sender. The choice of other sanctions is encoded as binary decision that takes the value 1 if the respondent chose at least one of the following options: lose job, pay fine, face prison sentence. Linear mixed-effects models with person, vignette deck, and country random effects. 95% confidence intervals in parentheses.

	Hatefulness score	Platform sanctions	Other sanctions
Topic: Liberals	−0.01	0.00	0.01
	[−0.02; 0.01]	[−0.02; 0.03]	[−0.01; 0.04]
Topic: Muslim immigrants	0.06*	0.09*	0.05*
	[0.04; 0.07]	[0.06; 0.11]	[0.03; 0.08]
Topic: Women	0.02*	0.03*	0.05*
	[0.00; 0.03]	[0.00; 0.06]	[0.03; 0.07]
Gender: Female	0.02*	0.08*	0.00
	[0.01; 0.04]	[0.04; 0.12]	[−0.03; 0.04]
Gender * Topic: Female * Liberals	0.02	0.03	0.01
	[−0.00; 0.04]	[−0.00; 0.07]	[−0.03; 0.04]
Gender * Topic: Female * Muslim immigrants	0.02*	0.02	0.02
	[0.00; 0.04]	[−0.02; 0.05]	[−0.01; 0.05]
Gender * Topic: Female * Women	0.03*	0.02	0.04*
	[0.01; 0.05]	[−0.02; 0.06]	[0.00; 0.07]
Num. obs.	10849	11120	11120
Num. groups: personid	1385	1390	1390
Num. groups: deckid	381	381	381
Var: personid (Intercept)	0.01	0.06	0.07
Var: deckid (Intercept)	0.00	0.00	0.00
Var: Residual	0.03	0.13	0.10

* Null hypothesis value outside the confidence interval.

Table S31: Effects of respondent gender X speech topic on hate speech perceptions and preferences for action (German sample). Perceived hatefulness is re-scaled to a 0-100 scale scale (originally 5-point scale). The choice of platform sanctions is encoded as binary decision that takes the value 1 if the respondent chose at least one of the following options: delete post, block sender, temporarily ban sender, permanently ban sender. The choice of other sanctions is encoded as binary decision that takes the value 1 if the respondent chose at least one of the following options: lose job, pay fine, face prison sentence. Linear mixed-effects models with person, vignette deck, and country random effects. 95% confidence intervals in parentheses.

	Hatefulness score	Platform sanctions	Other sanctions
Topic: Liberals	-0.02^*	-0.02	0.02
	$[-0.03; -0.00]$	$[-0.05; 0.01]$	$[-0.00; 0.04]$
Topic: Muslim immigrants	0.06^*	0.09^*	0.04^*
	$[0.04; 0.07]$	$[0.06; 0.12]$	$[0.02; 0.06]$
Topic: Women	0.03^*	0.06^*	0.02
	$[0.02; 0.04]$	$[0.03; 0.09]$	$[-0.00; 0.04]$
Gender: Female	0.02	0.10^*	0.04^*
	$[-0.00; 0.04]$	$[0.05; 0.14]$	$[0.01; 0.08]$
Gender * Topic: Female * Liberals	0.02^*	0.02	-0.01
	$[0.00; 0.04]$	$[-0.02; 0.06]$	$[-0.03; 0.02]$
Gender * Topic: Female * Muslim immigrants	0.02^*	0.01	0.00
	$[0.01; 0.04]$	$[-0.03; 0.04]$	$[-0.02; 0.03]$
Gender * Topic: Female * Women	0.03^*	0.03	0.03^*
	$[0.01; 0.05]$	$[-0.01; 0.06]$	$[0.01; 0.06]$
Num. obs.	9740	9856	9856
Num. groups: personid	1232	1232	1232
Num. groups: deckid	387	387	387
Var: personid (Intercept)	0.02	0.09	0.05
Var: deckid (Intercept)	0.00	0.00	0.00
Var: Residual	0.03	0.11	0.06

* Null hypothesis value outside the confidence interval.

Table S32: Effects of respondent gender X speech topic on hate speech perceptions and preferences for action (U.S. sample). Perceived hatefulness is re-scaled to a 0-100 scale scale (originally 5-point scale). The choice of platform sanctions is encoded as binary decision that takes the value 1 if the respondent chose at least one of the following options: delete post, block sender, temporarily ban sender, permanently ban sender. The choice of other sanctions is encoded as binary decision that takes the value 1 if the respondent chose at least one of the following options: lose job, pay fine, face prison sentence. Linear mixed-effects models with person, vignette deck, and country random effects. 95% confidence intervals in parentheses.

	Hatefulness score	Platform sanctions	Other sanctions
Topic: Liberals	0.05*	0.06*	0.05*
	[0.03; 0.06]	[0.03; 0.08]	[0.03; 0.07]
Topic: Muslim immigrants	0.15*	0.18*	0.10*
	[0.14; 0.16]	[0.15; 0.20]	[0.08; 0.12]
Topic: Women	0.09*	0.11*	0.08*
	[0.07; 0.10]	[0.08; 0.13]	[0.06; 0.10]
Ideology (respondent): Right	0.03*	−0.03	0.04*
	[0.01; 0.05]	[−0.07; 0.00]	[0.01; 0.07]
Ideology (respondent) * Topic: Right * Liberals	−0.12*	−0.13*	−0.07*
	[−0.14; −0.10]	[−0.17; −0.09]	[−0.10; −0.04]
Ideology (respondent) * Topic: Right * Muslim immigrants	−0.19*	−0.18*	−0.12*
	[−0.21; −0.17]	[−0.22; −0.15]	[−0.15; −0.10]
Ideology (respondent) * Topic: Right * Women	−0.11*	−0.10*	−0.07*
	[−0.13; −0.09]	[−0.14; −0.06]	[−0.09; −0.04]
Num. obs.	11311	11488	11488
Num. groups: personid	1433	1436	1436
Num. groups: deckid	671	671	671
Num. groups: country	2	2	2
Var: personid (Intercept)	0.02	0.07	0.05
Var: deckid (Intercept)	0.00	0.00	0.00
Var: country (Intercept)	0.00	0.00	0.01
Var: Residual	0.03	0.12	0.07

* Null hypothesis value outside the confidence interval.

Table S33: Effects of respondent ideology X speech topic on hate speech perceptions and preferences for action (pooled sample). Perceived hatefulness is re-scaled to a 0-100 scale scale (originally 5-point scale). The choice of platform sanctions is encoded as binary decision that takes the value 1 if the respondent chose at least one of the following options: delete post, block sender, temporarily ban sender, permanently ban sender. The choice of other sanctions is encoded as binary decision that takes the value 1 if the respondent chose at least one of the following options: lose job, pay fine, face prison sentence. Linear mixed-effects models with person, vignette deck, and country random effects. 95% confidence intervals in parentheses.

	Hatefulness score	Platform sanctions	Other sanctions
Topic: Liberals	0.04^*	0.07^*	0.06^*
	[0.02; 0.06]	[0.03; 0.10]	[0.03; 0.10]
Topic: Muslim immigrants	0.13^*	0.17^*	0.12^*
	[0.11; 0.15]	[0.13; 0.21]	[0.08; 0.15]
Topic: Women	0.06^*	0.07^*	0.10^*
	[0.04; 0.08]	[0.03; 0.11]	[0.07; 0.14]
Ideology (respondent): Right	0.05^*	0.03	0.06^*
	[0.02; 0.08]	[−0.03; 0.09]	[0.00; 0.11]
Ideology (respondent) * Topic: Right * Liberals	-0.13^*	-0.14^*	-0.08^*
	[−0.16; −0.10]	[−0.20; −0.08]	[−0.13; −0.03]
Ideology (respondent) * Topic: Right * Muslim immigrants	-0.19^*	-0.20^*	-0.15^*
	[−0.22; −0.16]	[−0.26; −0.14]	[−0.20; −0.10]
Ideology (respondent) * Topic: Right * Women	-0.09^*	-0.11^*	−0.05
	[−0.12; −0.06]	[−0.17; −0.05]	[−0.10; 0.00]
Num. obs.	4714	4808	4808
Num. groups: personid	598	601	601
Num. groups: deckid	313	313	313
Var: personid (Intercept)	0.02	0.06	0.05
Var: deckid (Intercept)	0.00	0.00	0.00
Var: Residual	0.03	0.13	0.10

* Null hypothesis value outside the confidence interval.

Table S34: Effects of respondent ideology X speech topic on hate speech perceptions and preferences for action (German sample). Perceived hatefulness is re-scaled to a 0-100 scale scale (originally 5-point scale). The choice of platform sanctions is encoded as binary decision that takes the value 1 if the respondent chose at least one of the following options: delete post, block sender, temporarily ban sender, permanently ban sender. The choice of other sanctions is encoded as binary decision that takes the value 1 if the respondent chose at least one of the following options: lose job, pay fine, face prison sentence. Linear mixed-effects models with person, vignette deck, and country random effects. 95% confidence intervals in parentheses.

	Hatefulness score	Platform sanctions	Other sanctions
Topic: Liberals	0.05*	0.05*	0.04*
	[0.04; 0.07]	[0.02; 0.09]	[0.02; 0.07]
Topic: Muslim immigrants	0.16*	0.19*	0.09*
	[0.14; 0.18]	[0.16; 0.23]	[0.07; 0.12]
Topic: Women	0.11*	0.13*	0.05*
	[0.09; 0.12]	[0.10; 0.17]	[0.03; 0.08]
Ideology (respondent): Right	0.02	−0.07*	0.03
	[−0.00; 0.04]	[−0.12; −0.02]	[−0.01; 0.07]
Ideology (respondent) * Topic: Right * Liberals	−0.12*	−0.12*	−0.06*
	[−0.14; −0.10]	[−0.17; −0.08]	[−0.09; −0.03]
Ideology (respondent) * Topic: Right * Muslim immigrants	−0.19*	−0.18*	−0.11*
	[−0.21; −0.17]	[−0.23; −0.13]	[−0.14; −0.08]
Ideology (respondent) * Topic: Right * Women	−0.13*	−0.10*	−0.07*
	[−0.15; −0.10]	[−0.15; −0.06]	[−0.10; −0.03]
Num. obs.	6597	6680	6680
Num. groups: personid	835	835	835
Num. groups: deckid	358	358	358
Var: personid (Intercept)	0.02	0.08	0.04
Var: deckid (Intercept)	0.00	0.00	0.00
Var: Residual	0.03	0.12	0.06

* Null hypothesis value outside the confidence interval.

Table S35: Effects of respondent ideology X speech topic on hate speech perceptions and preferences for action (U.S. sample). Perceived hatefulness is re-scaled to a 0-100 scale scale (originally 5-point scale). The choice of platform sanctions is encoded as binary decision that takes the value 1 if the respondent chose at least one of the following options: delete post, block sender, temporarily ban sender, permanently ban sender. The choice of other sanctions is encoded as binary decision that takes the value 1 if the respondent chose at least one of the following options: lose job, pay fine, face prison sentence. Linear mixed-effects models with person, vignette deck, and country random effects. 95% confidence intervals in parentheses.

	Hatefulness score	Platform sanctions	Other sanctions
Topic: Liberals	0.01	−0.01	0.04*
	[−0.01; 0.03]	[−0.06; 0.03]	[0.01; 0.07]
Topic: Muslim immigrants	0.10*	0.10*	0.09*
	[0.08; 0.13]	[0.05; 0.15]	[0.06; 0.12]
Topic: Women	0.07*	0.08*	0.08*
	[0.05; 0.09]	[0.04; 0.13]	[0.04; 0.11]
Ethnicity: White	0.00	−0.07*	−0.04*
	[−0.02; 0.03]	[−0.13; −0.01]	[−0.08; −0.00]
Ethnicity * Topic: white * Liberals	−0.02	0.00	−0.03
	[−0.04; 0.01]	[−0.04; 0.05]	[−0.07; 0.00]
Ethnicity * Topic: white * Muslim immigrants	−0.04*	−0.00	−0.06*
	[−0.07; −0.02]	[−0.05; 0.05]	[−0.09; −0.02]
Ethnicity * Topic: white * Women	−0.03*	−0.01	−0.05*
	[−0.05; −0.00]	[−0.06; 0.04]	[−0.09; −0.01]
Num. obs.	9740	9856	9856
Num. groups: personid	1232	1232	1232
Num. groups: deckid	387	387	387
Var: personid (Intercept)	0.02	0.09	0.05
Var: deckid (Intercept)	0.00	0.00	0.00
Var: Residual	0.03	0.11	0.06

* Null hypothesis value outside the confidence interval.

Table S36: Effects of respondent race X speech topic on hate speech perceptions and preferences for action (U.S. sample). Perceived hatefulness is re-scaled to a 0-100 scale scale (originally 5-point scale). The choice of platform sanctions is encoded as binary decision that takes the value 1 if the respondent chose at least one of the following options: delete post, block sender, temporarily ban sender, permanently ban sender. The choice of other sanctions is encoded as binary decision that takes the value 1 if the respondent chose at least one of the following options: lose job, pay fine, face prison sentence. Linear mixed-effects models with person, vignette deck, and country random effects. 95% confidence intervals in parentheses.

(a) Marginal means by respondent gender

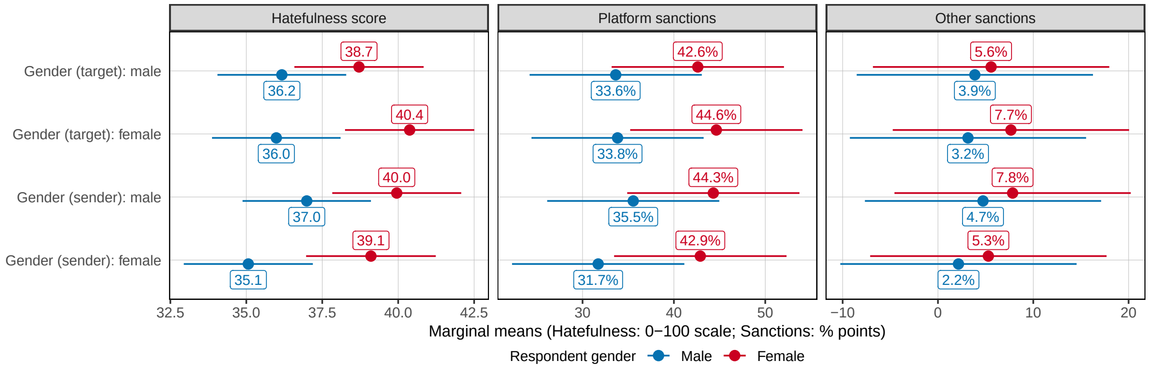

(b) Difference in marginal means by respondent gender

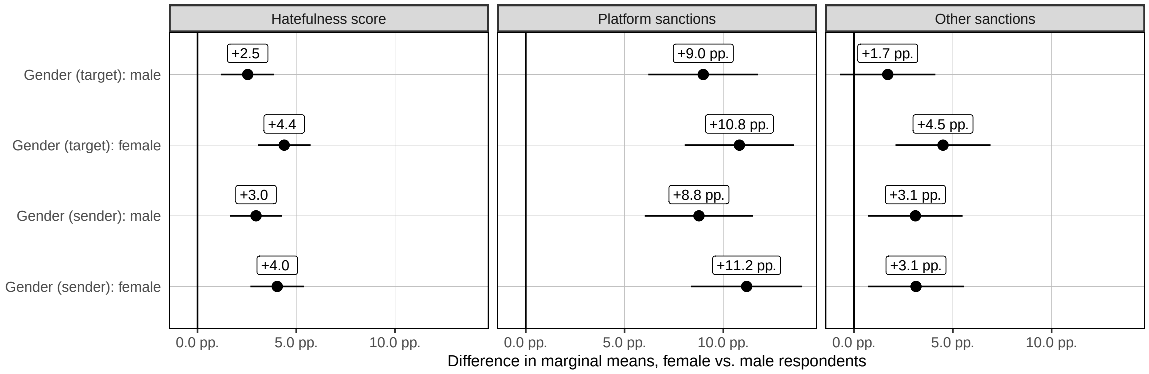

Figure S30: Estimated marginal means based on the interaction of respondent's and sender/target's gender on citizens' perceptions and preferred platform-side and other action (pooled sample). Error bars represent 95% confidence intervals. For the differences in marginal means, positive differences imply that female respondents score higher on the outcome than male respondents.

(a) Marginal means by respondent gender

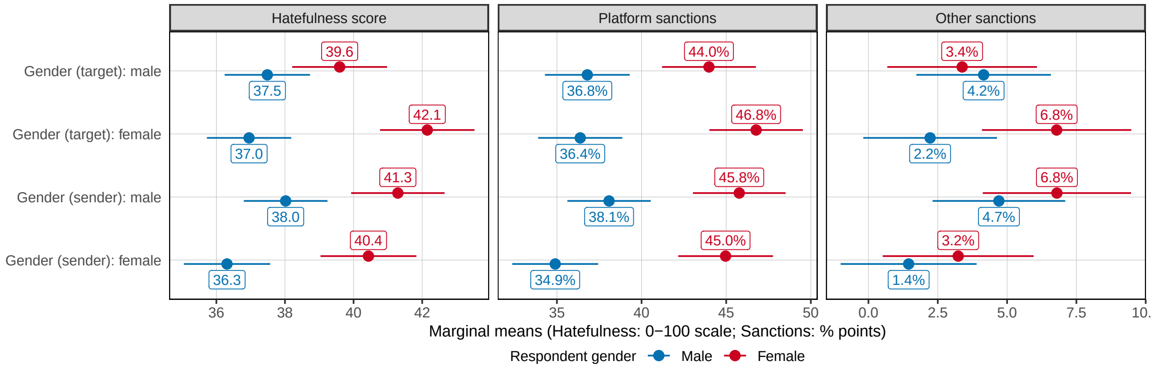

(b) Difference in marginal means by respondent gender

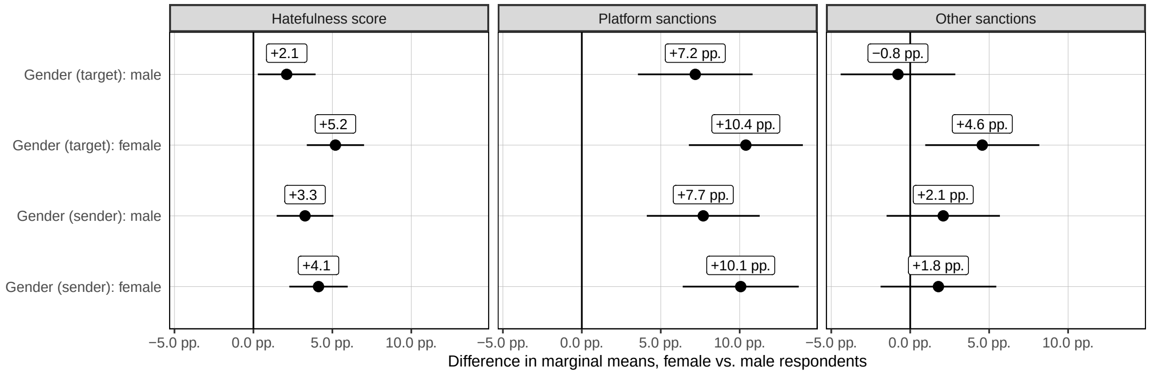

Figure S31: Estimated marginal means based on the interaction of respondent's and sender/target's gender on citizens' perceptions and preferred platform-side and other action (German sample). Error bars represent 95% confidence intervals. For the differences in marginal means, positive differences imply that female respondents score higher on the outcome than male respondents.

(a) Marginal means by respondent gender

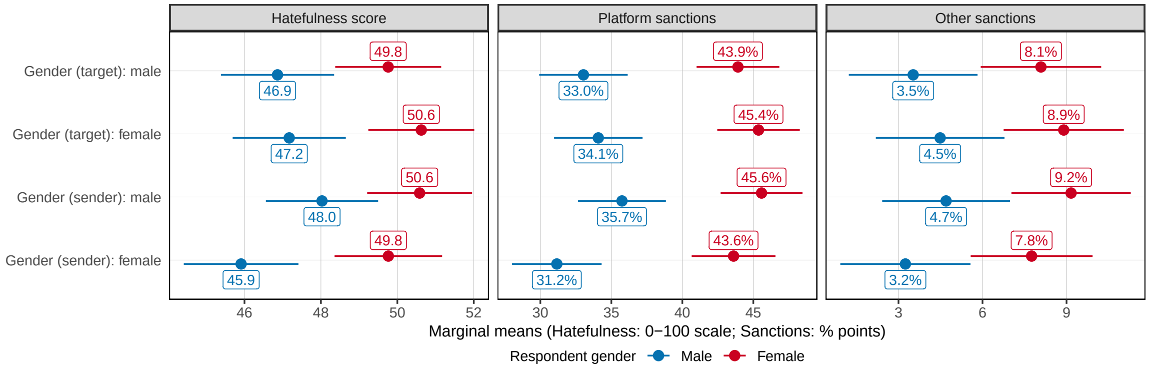

(b) Difference in marginal means by respondent gender

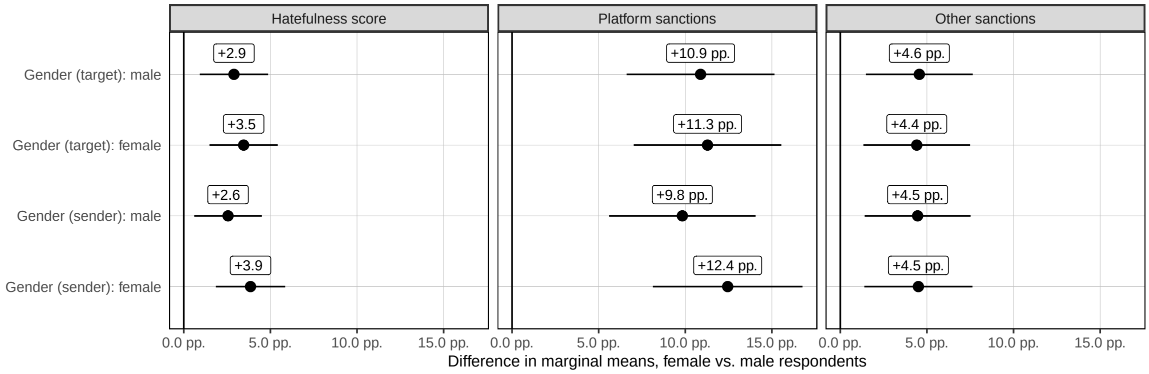

Figure S32: Estimated marginal means based on the interaction of respondent's and sender/target's gender on citizens' perceptions and preferred platform-side and other action (U.S. sample). Error bars represent 95% confidence intervals. For the differences in marginal means, positive differences imply that female respondents score higher on the outcome than male respondents.

(a) Marginal means by respondent ideology

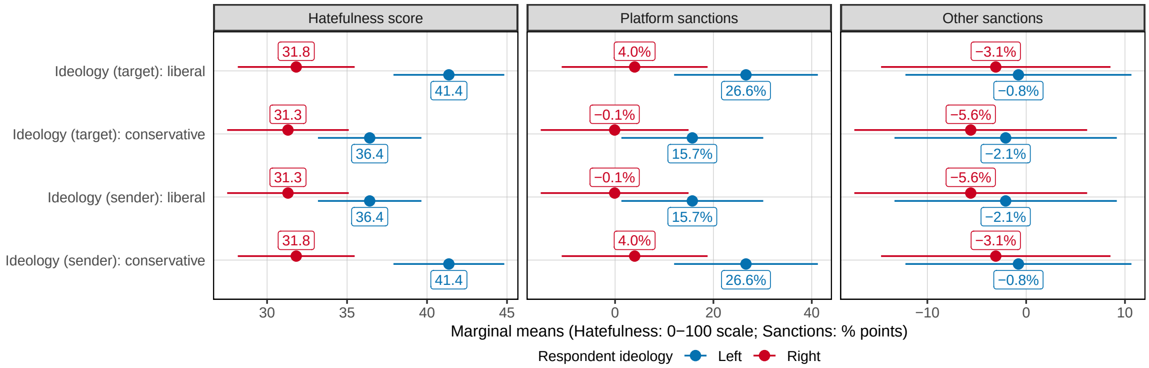

(b) Difference in marginal means by respondent ideology

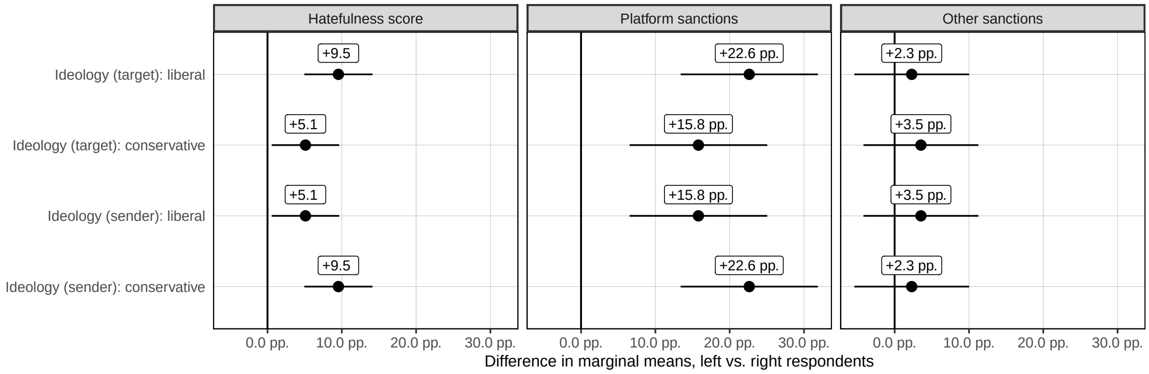

Figure S33: Estimated marginal means based on the interaction of respondent's and sender/target's ideology on citizens' perceptions and preferred platform-side and other action (pooled sample). Error bars represent 95% confidence intervals. For the differences in marginal means, positive differences imply that female respondents score higher on the outcome than male respondents.

(a) Marginal means by respondent ideology

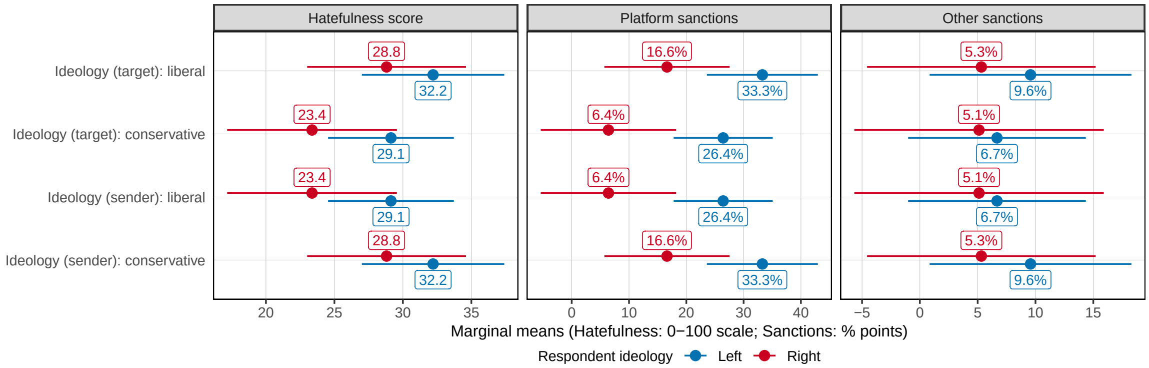

(b) Difference in marginal means by respondent ideology

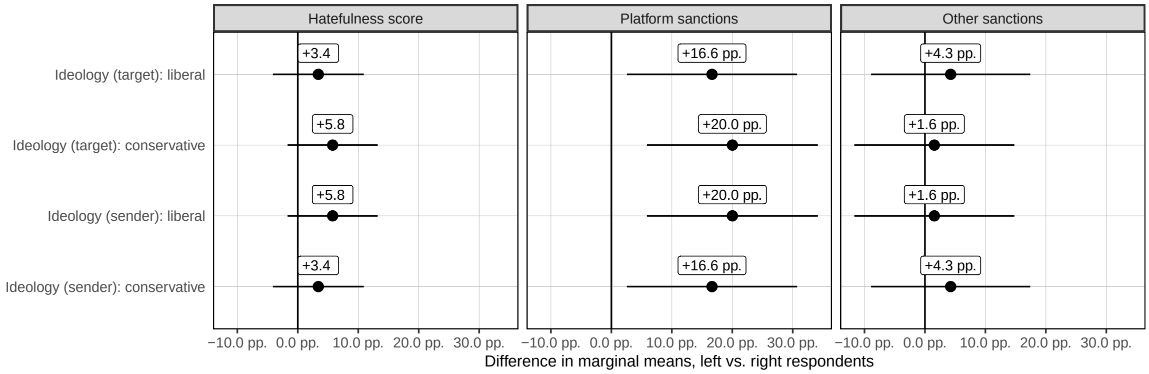

Figure S34: Estimated marginal means based on the interaction of respondent's and sender/target's ideology on citizens' perceptions and preferred platform-side and other action (German sample). Error bars represent 95% confidence intervals. For the differences in marginal means, positive differences imply that female respondents score higher on the outcome than male respondents.

(a) Marginal means by respondent ideology

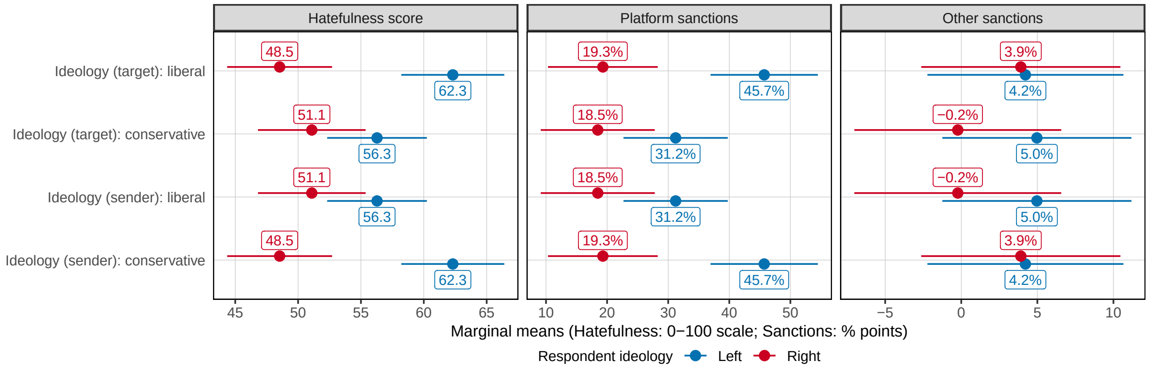

(b) Difference in marginal means by respondent ideology

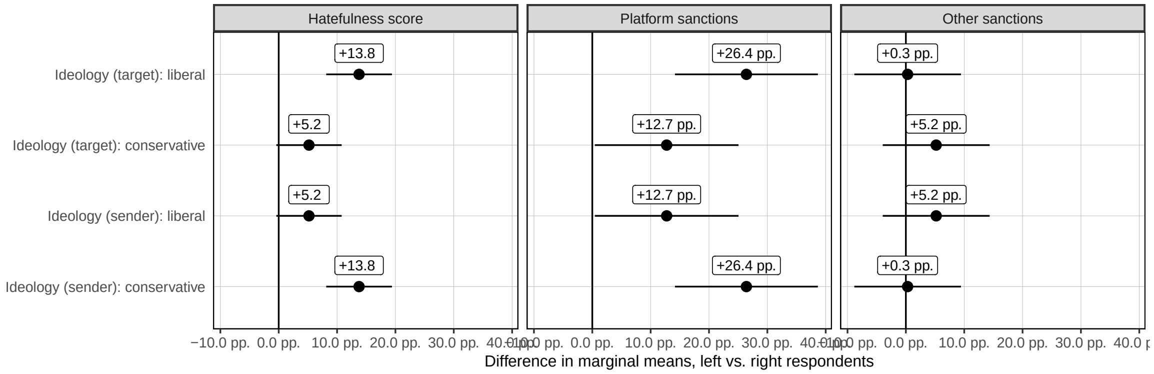

Figure S35: Estimated marginal means based on the interaction of respondent's and sender/target's ideology on citizens' perceptions and preferred platform-side and other action (U.S. sample). Error bars represent 95% confidence intervals. For the differences in marginal means, positive differences imply that female respondents score higher on the outcome than male respondents.

(a) Marginal means by respondent gender

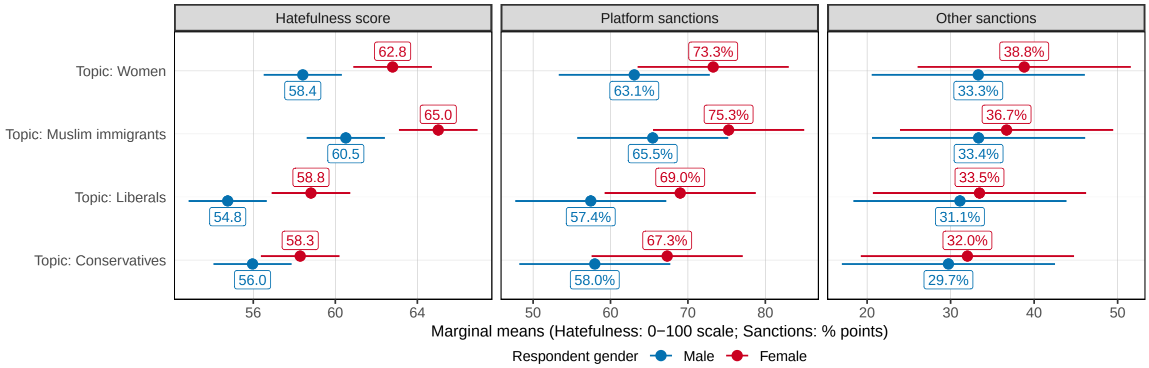

(b) Difference in marginal means by respondent gender

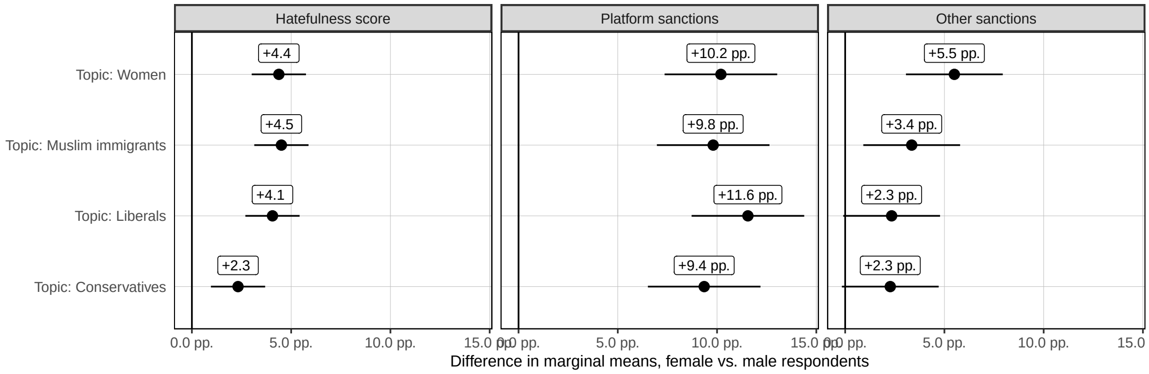

Figure S36: Estimated marginal means based on the interaction of respondent's gender and the topic of the message on citizens' perceptions and preferred platform-side and other action (pooled sample). Error bars represent 95% confidence intervals. For the differences in marginal means, positive differences imply that female respondents score higher on the outcome than male respondents.

(a) Marginal means by respondent gender

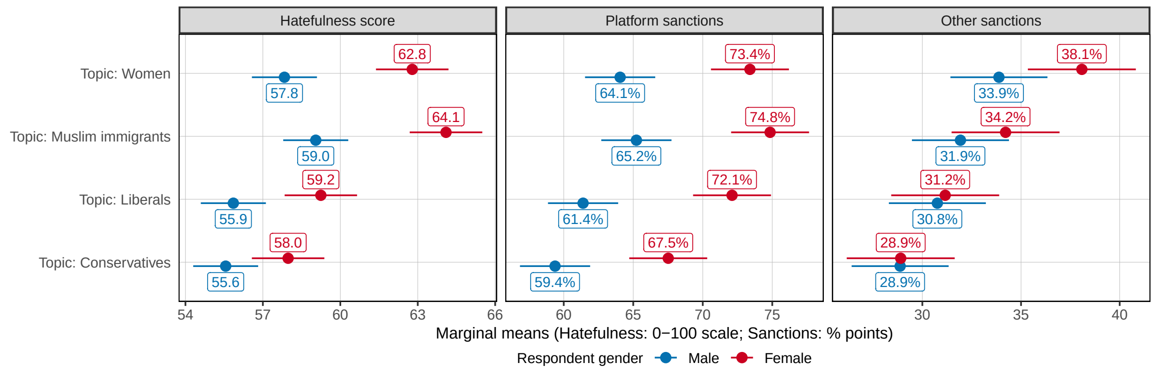

(b) Difference in marginal means by respondent gender

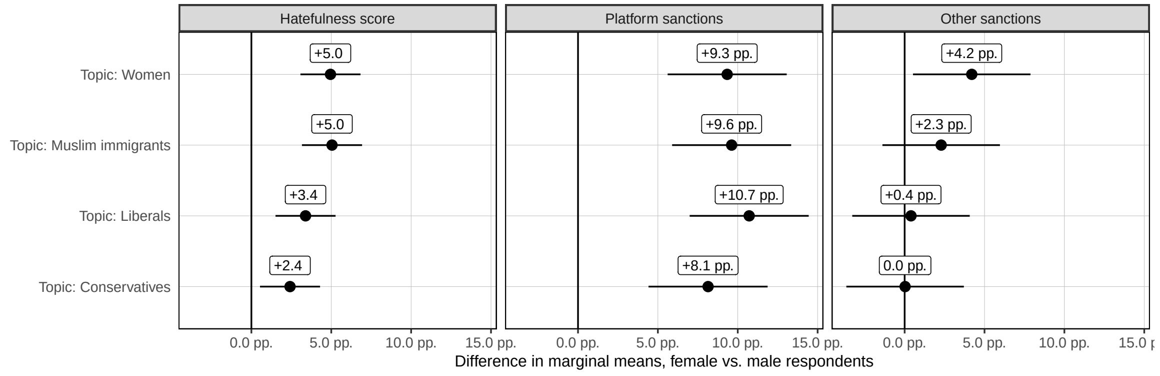

Figure S37: Estimated marginal means based on the interaction of respondent's gender and the topic of the message on citizens' perceptions and preferred platform-side and other action (German sample). Error bars represent 95% confidence intervals. For the differences in marginal means, positive differences imply that female respondents score higher on the outcome than male respondents.

(a) Marginal means by respondent gender

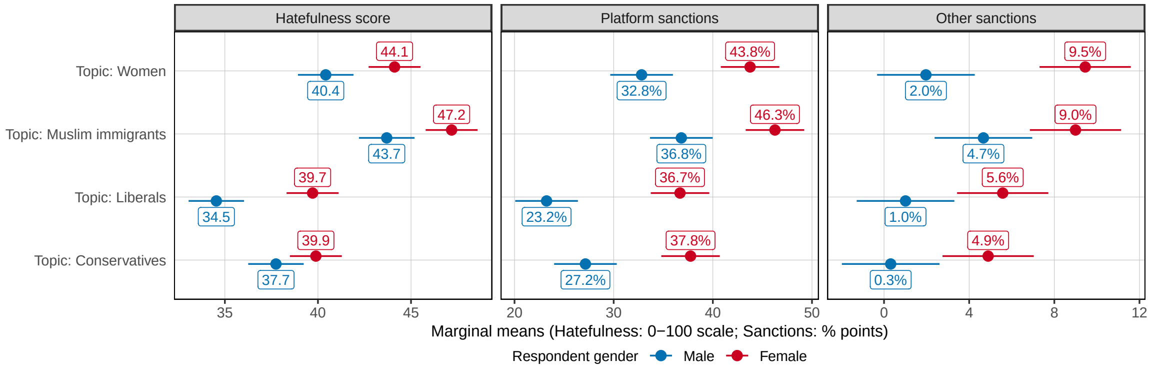

(b) Difference in marginal means by respondent gender

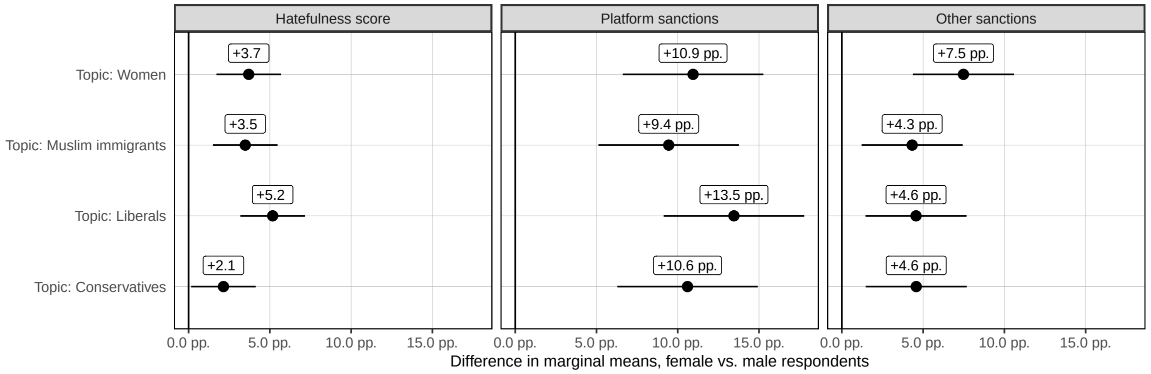

Figure S38: Estimated marginal means based on the interaction of respondent's gender and the topic of the message on citizens' perceptions and preferred platform-side and other action (U.S. sample). Error bars represent 95% confidence intervals. For the differences in marginal means, positive differences imply that female respondents score higher on the outcome than male respondents.

(a) Marginal means by respondent ideology

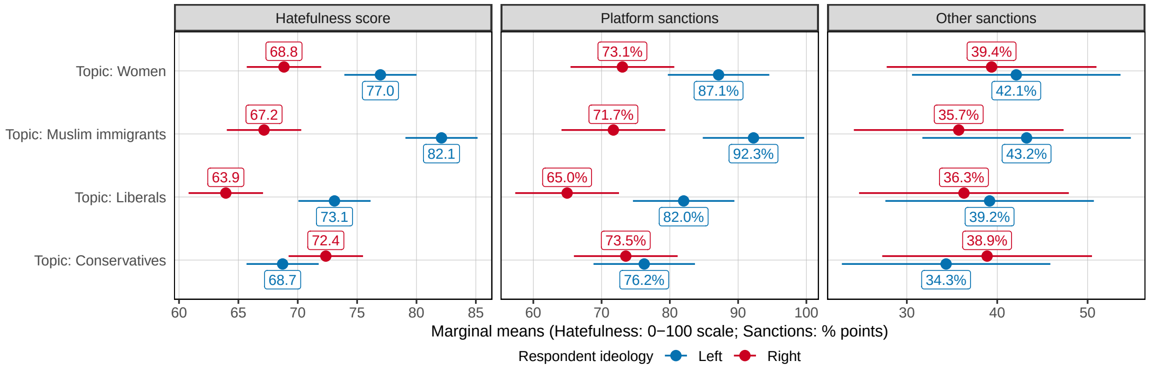

(b) Difference in marginal means by respondent ideology

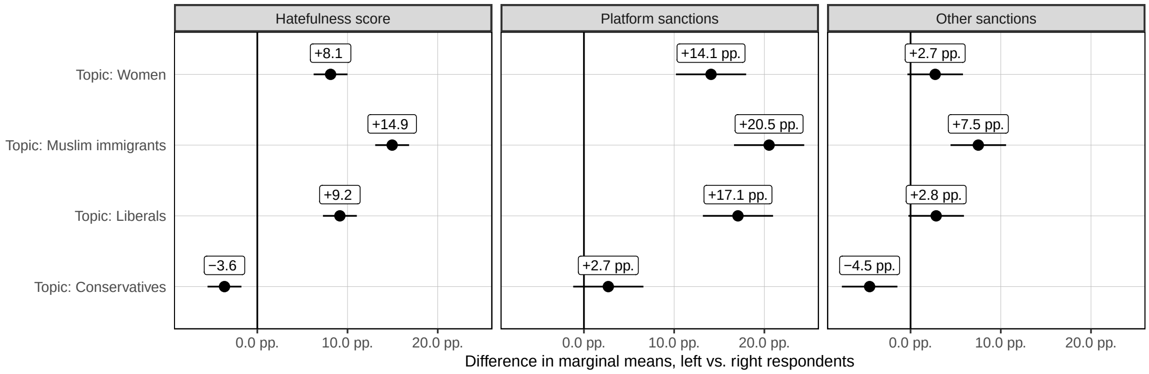

Figure S39: Estimated marginal means based on the interaction of respondent's ideology and the topic of the message on citizens' perceptions and preferred platform-side and other action (pooled sample). Error bars represent 95% confidence intervals. For the differences in marginal means, positive differences imply that female respondents score higher on the outcome than male respondents.

(a) Marginal means by respondent ideology

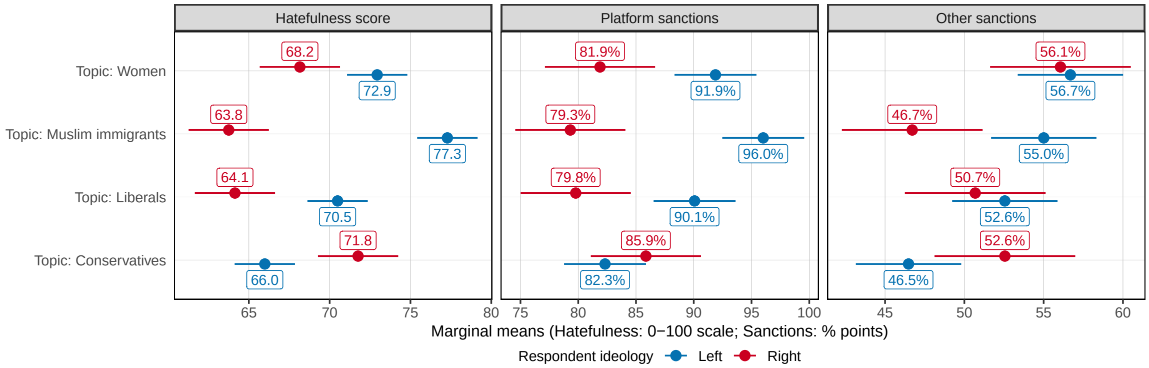

(b) Difference in marginal means by respondent ideology

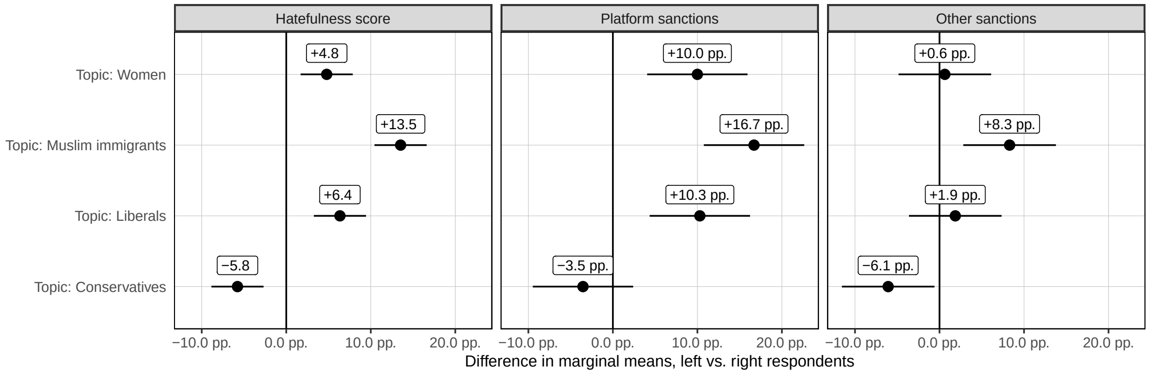

Figure S40: Estimated marginal means based on the interaction of respondent's ideology and the topic of the message on citizens' perceptions and preferred platform-side and other action (German sample). Error bars represent 95% confidence intervals. For the differences in marginal means, positive differences imply that female respondents score higher on the outcome than male respondents.

(a) Marginal means by respondent ideology

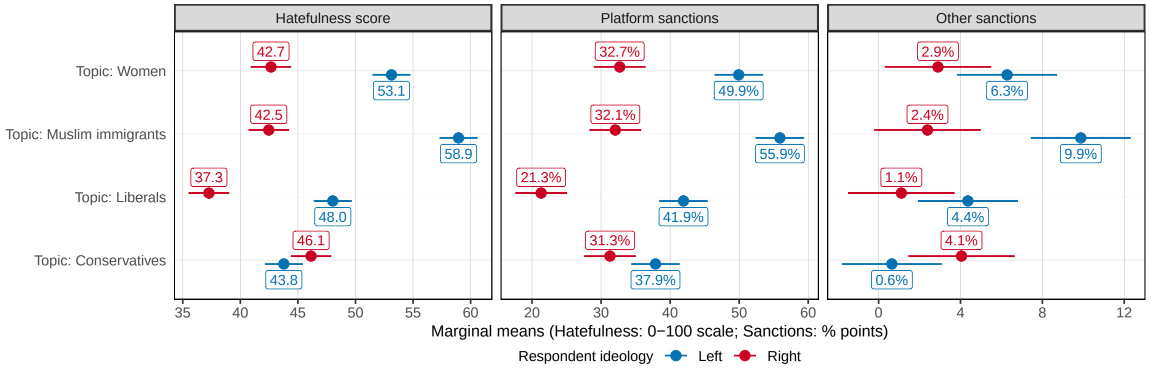

(b) Difference in marginal means by respondent ideology

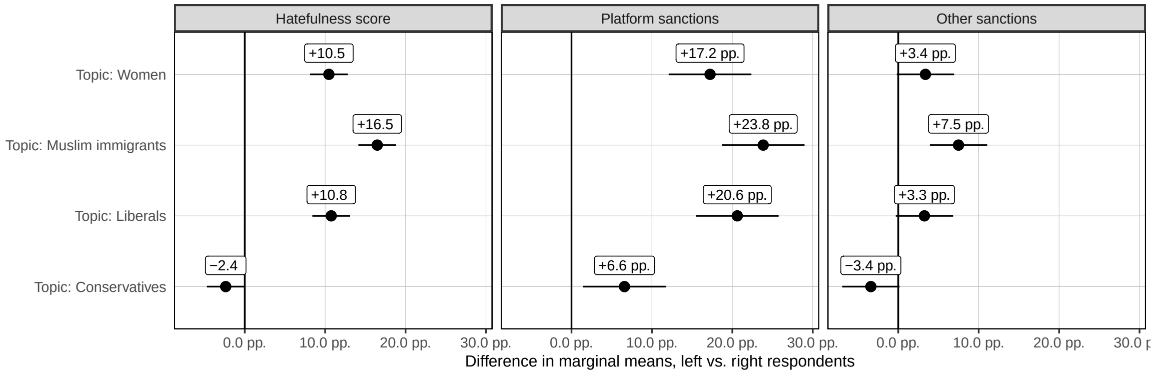

Figure S41: Estimated marginal means based on the interaction of respondent's ideology and the topic of the message on citizens' perceptions and preferred platform-side and other action (U.S. sample). Error bars represent 95% confidence intervals. For the differences in marginal means, positive differences imply that female respondents score higher on the outcome than male respondents.

(a) Marginal means by respondent race

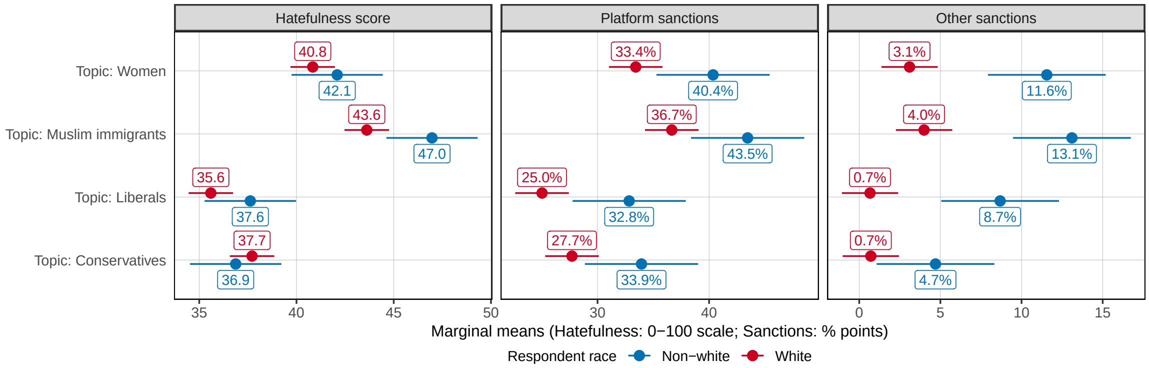

(b) Difference in marginal means by respondent race

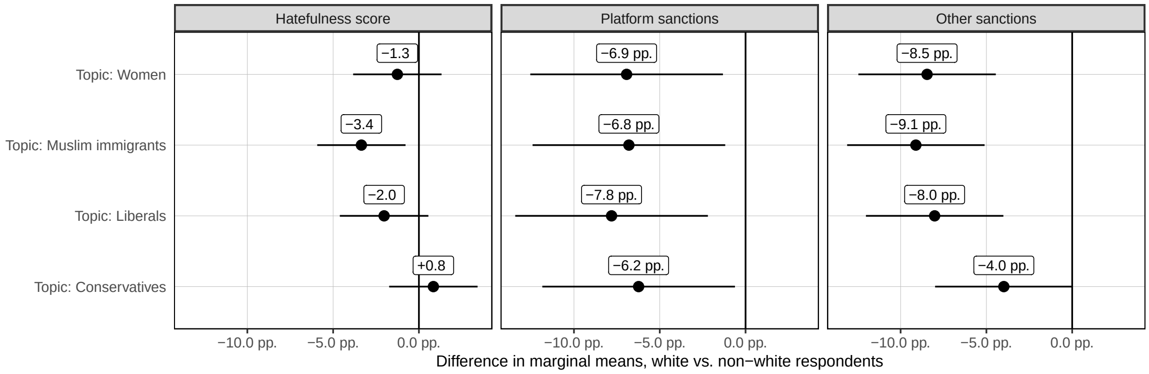

Figure S42: Estimated marginal means based on the interaction of respondent's race and the topic of the message on citizens' perceptions and preferred platform-side and other action (U.S. sample). Error bars represent 95% confidence intervals. For the differences in marginal means, positive differences imply that female respondents score higher on the outcome than male respondents.

D.4 Framing experiment

Table S37: Descriptive statistics of respondent characteristics, by treatment group status (framing experiment)

		Neutral (N=870)		Gov. reg. (N=877)		Free speech (N=875)	
		N	Pct.	N	Pct.	N	Pct.
Gender	Male	437	50.2	440	50.2	466	53.3
	Female	433	49.8	437	49.8	409	46.7
Age	18-29	44	5.1	73	8.3	68	7.8
	30-49	233	26.8	272	31.0	267	30.5
	50-69	424	48.7	374	42.6	381	43.5
	70+	120	13.8	113	12.9	111	12.7
Education	Low	140	16.1	147	16.8	153	17.5
	Intermediate	341	39.2	343	39.1	341	39.0
	High	388	44.6	386	44.0	379	43.3
Political interest	Low	88	10.1	103	11.7	131	15.0
	Intermediate	182	20.9	192	21.9	188	21.5
	High	547	62.9	538	61.3	506	57.8
Social media user	No	155	17.8	135	15.4	163	18.6
	Yes	675	77.6	710	81.0	681	77.8
Ideology	Left	270	31.0	297	33.9	264	30.2
	Center	336	38.6	336	38.3	341	39.0
	Right	207	23.8	193	22.0	205	23.4
Hate experience	No	707	81.3	711	81.1	717	81.9
	Yes	114	13.1	121	13.8	110	12.6
Hate witness	No	562	64.6	557	63.5	551	63.0
	Yes	259	29.8	275	31.4	276	31.5
Talk politics freely	No	363	41.7	356	40.6	362	41.4
	Yes	460	52.9	486	55.4	475	54.3

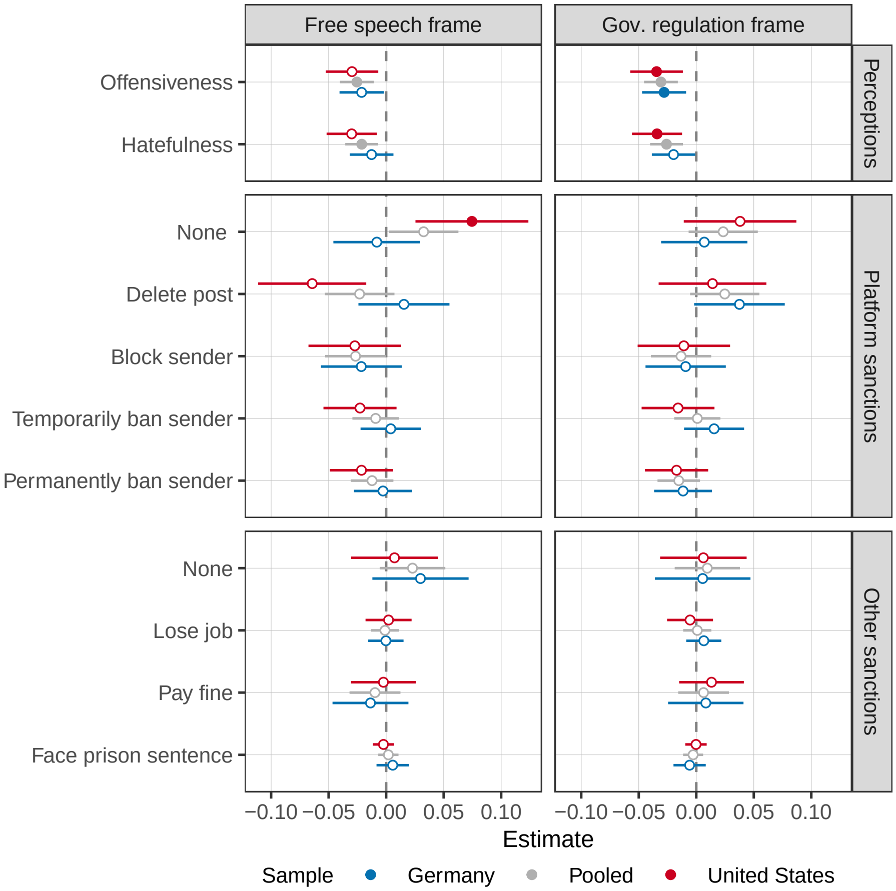

Figure S43: Estimated effects of free speech advocacy and government regulation frames (vs. neutral frame) on respondents' perceptions and preferred platform-side and other action, by country. Perceived hatefulness and offensiveness are measured on 5-point scales (re-scaled to 0-1), preferences for platform action and further penalties are binary measures. Error bars represent 95% confidence intervals. Filled circles indicate that the effect remains significant after *p*-value adjustment by controlling the false discovery rate. See Supplementary Tables S38 to S40 for detailed regression results.

	Offensive	Hateful	No action	Delete	Block	Temp. ban	Perm. ban	No penalties	Lose job	Fine	Prison
Intercept	0.43***	0.45***	0.62***	0.19***	0.12**	0.10**	0.06*	0.78***	0.06**	0.07	0.05**
	(0.02)	(0.02)	(0.06)	(0.05)	(0.04)	(0.03)	(0.03)	(0.07)	(0.02)	(0.06)	(0.02)
Government regulation	−0.03***	−0.03***	0.02	0.02	−0.01	0.00	−0.02	0.01	0.00	0.01	−0.00
	(0.01)	(0.01)	(0.02)	(0.02)	(0.01)	(0.01)	(0.01)	(0.01)	(0.01)	(0.01)	(0.00)
Free speech advocacy	−0.03***	−0.02**	0.03*	−0.02	−0.03*	−0.01	−0.01	0.02	−0.00	−0.01	0.00
	(0.01)	(0.01)	(0.02)	(0.02)	(0.01)	(0.01)	(0.01)	(0.01)	(0.01)	(0.01)	(0.00)
Num. obs.	17497	17343	17656	17656	17656	17656	17656	17656	17656	17656	17656
Num. groups: personid	2204	2204	2207	2207	2207	2207	2207	2207	2207	2207	2207
Num. groups: deckid	747	747	747	747	747	747	747	747	747	747	747
Num. groups: country	2	2	2	2	2	2	2	2	2	2	2
Var: personid (Intercept)	0.02	0.01	0.07	0.07	0.05	0.03	0.02	0.07	0.01	0.04	0.00
Var: deckid (Intercept)	0.00	0.00	0.00	0.00	0.00	0.00	0.00	0.00	0.00	0.00	0.00
Var: country (Intercept)	0.00	0.00	0.00	0.00	0.00	0.00	0.00	0.01	0.00	0.01	0.00
Var: Residual	0.03	0.03	0.12	0.14	0.11	0.09	0.06	0.09	0.02	0.08	0.02

$^{***}p < 0.001$; $^{**}p < 0.01$; $^{*}p < 0.05$

Table S38: Effects of vignette task frame on citizens' hate speech perceptions and preferences for action (pooled sample, with covariates). Offensiveness and hatefulness scores were measured on five-point scales and rescaled to 0-1. Support for action was measured on a binary scale, 0-1. Linear mixed-effects models with person, vignette deck, and country random effects. Standard error in parentheses.

	Offensive	Hateful	No action	Delete	Block	Temp. ban	Perm. ban	No penalties	Lose job	Fine	Prison
Intercept	0.41^{***}	0.42^{***}	0.70^{***}	0.10	0.10	0.08^{*}	0.05	0.77^{***}	0.04	0.06	0.05^{*}
	(0.03)	(0.03)	(0.06)	(0.06)	(0.05)	(0.04)	(0.04)	(0.06)	(0.02)	(0.05)	(0.02)
Government regulation	-0.03^{**}	-0.02^{*}	0.01	0.04	-0.01	0.02	-0.01	0.01	0.01	0.01	-0.01
	(0.01)	(0.01)	(0.02)	(0.02)	(0.02)	(0.01)	(0.01)	(0.02)	(0.01)	(0.02)	(0.01)
Free speech advocacy	-0.02^{*}	-0.01	-0.01	0.02	-0.02	0.00	-0.00	0.03	-0.00	-0.01	0.01
	(0.01)	(0.01)	(0.02)	(0.02)	(0.02)	(0.01)	(0.01)	(0.02)	(0.01)	(0.02)	(0.01)
Num. obs.	9601	9502	9720	9720	9720	9720	9720	9720	9720	9720	9720
Num. groups: personid	1212	1212	1215	1215	1215	1215	1215	1215	1215	1215	1215
Num. groups: deckid	375	375	375	375	375	375	375	375	375	375	375
Var: personid (Intercept)	0.01	0.01	0.06	0.06	0.05	0.02	0.02	0.08	0.01	0.04	0.01
Var: deckid (Intercept)	0.00	0.00	0.00	0.00	0.00	0.00	0.00	0.00	0.00	0.00	0.00
Var: Residual	0.04	0.03	0.13	0.16	0.11	0.10	0.07	0.10	0.02	0.10	0.03

$^{***}p < 0.001$; $^{**}p < 0.01$; $^{*}p < 0.05$

Table S39: Effects of vignette task frame on citizens' hate speech perceptions and preferences for action (German sample, with covariates). Offensiveness and hatefulness scores were measured on five-point scales and rescaled to 0-1. Support for action was measured on a binary scale, 0-1. Linear mixed-effects models with person, vignette deck, and country random effects. Standard error in parentheses.

	Offensive	Hateful	No action	Delete	Block	Temp. ban	Perm. ban	No penalties	Lose job	Fine	Prison
Intercept	0.46***	0.46***	0.54***	0.29***	0.11	0.10	0.08	0.78***	0.08*	0.11*	0.04*
	(0.04)	(0.03)	(0.08)	(0.07)	(0.06)	(0.05)	(0.04)	(0.06)	(0.03)	(0.04)	(0.02)
Government regulation	−0.03**	−0.03**	0.04	0.01	−0.01	−0.02	−0.02	0.01	−0.01	0.01	−0.00
	(0.01)	(0.01)	(0.02)	(0.02)	(0.02)	(0.02)	(0.01)	(0.02)	(0.01)	(0.01)	(0.00)
Free speech advocacy	−0.03*	−0.03**	0.07**	−0.06**	−0.03	−0.02	−0.02	0.01	0.00	−0.00	−0.00
	(0.01)	(0.01)	(0.03)	(0.02)	(0.02)	(0.02)	(0.01)	(0.02)	(0.01)	(0.01)	(0.00)
Num. obs.	7896	7841	7936	7936	7936	7936	7936	7936	7936	7936	7936
Num. groups: personid	992	992	992	992	992	992	992	992	992	992	992
Num. groups: deckid	372	372	372	372	372	372	372	372	372	372	372
Var: personid (Intercept)	0.02	0.01	0.09	0.08	0.05	0.03	0.02	0.05	0.01	0.03	0.00
Var: deckid (Intercept)	0.00	0.00	0.00	0.00	0.00	0.00	0.00	0.00	0.00	0.00	0.00
Var: Residual	0.03	0.03	0.11	0.12	0.10	0.08	0.06	0.07	0.03	0.05	0.01

$^{***}p < 0.001$; $^{**}p < 0.01$; $^{*}p < 0.05$

Table S40: Effects of vignette task frame on citizens' hate speech perceptions and preferences for action (U.S. sample, with covariates). Offensiveness and hatefulness scores were measured on five-point scales and rescaled to 0-1. Support for action was measured on a binary scale, 0-1. Linear mixed-effects models with person, vignette deck, and country random effects. Standard error in parentheses.

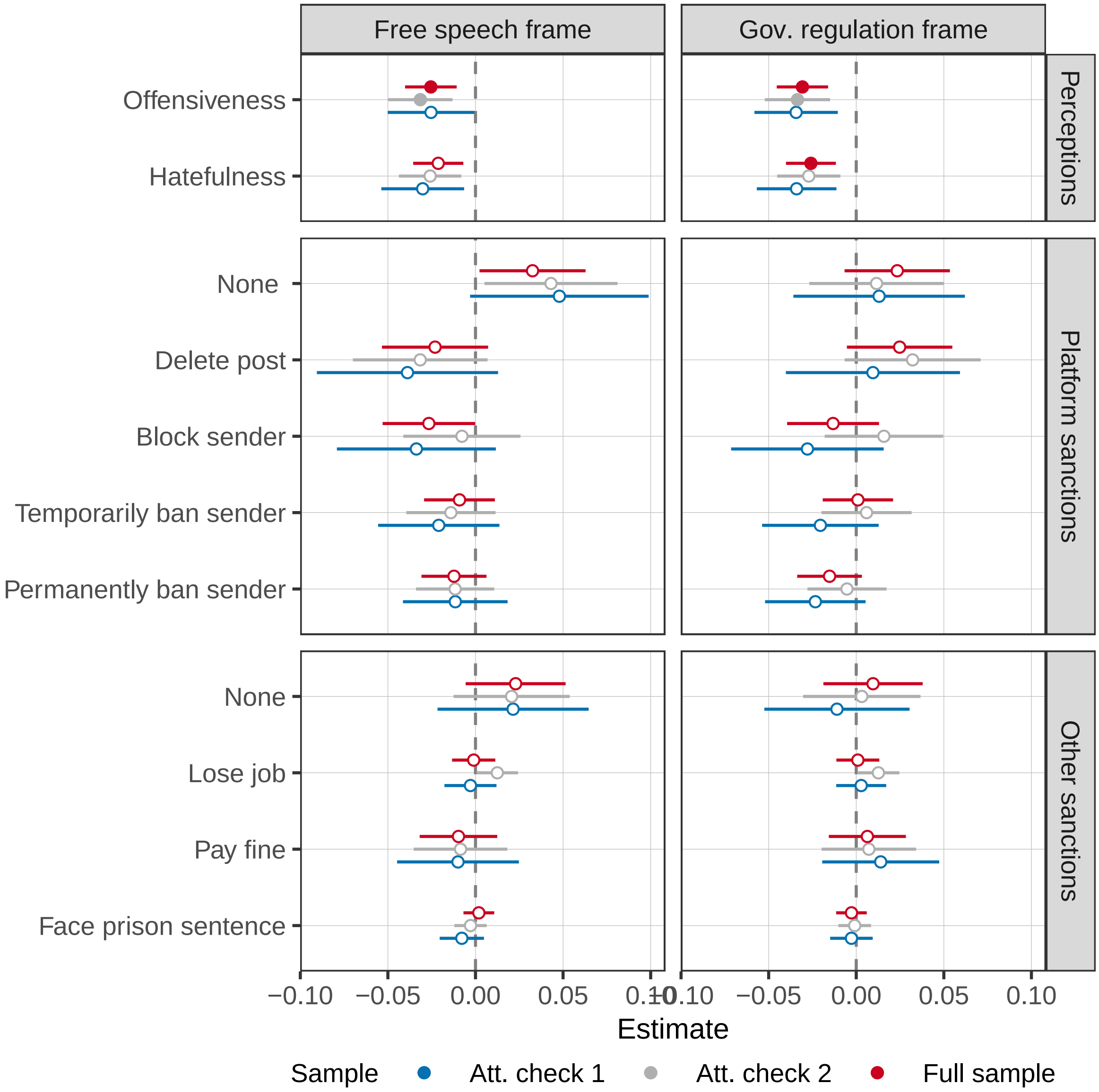

Figure S44: Estimated effects of free speech advocacy and government regulation frames (vs. neutral frame) on citizens' perceptions and preferred platform-side and other action (pooled sample; by attention check success). Attention check 1 refers to a question about smartphone ownership that was asked just before the framing was shown to respondents and that required respondents to type 'read' into the open text field to pass successfully. The attention check question was presented to all respondents irrespective of treatment status. Attention check 2 (not pre-registered) is based on the time spent on the vignette introduction. Passing this check was defined as spending at least 10sec on the neutral condition or at least 15sec on one of the frame treatment conditions. Perceived hatefulness and offensiveness are measured on 5-point scales (re-scaled to 0-1 scale), preferences for platform action and further penalties are binary measures. Error bars represent 95% confidence intervals.

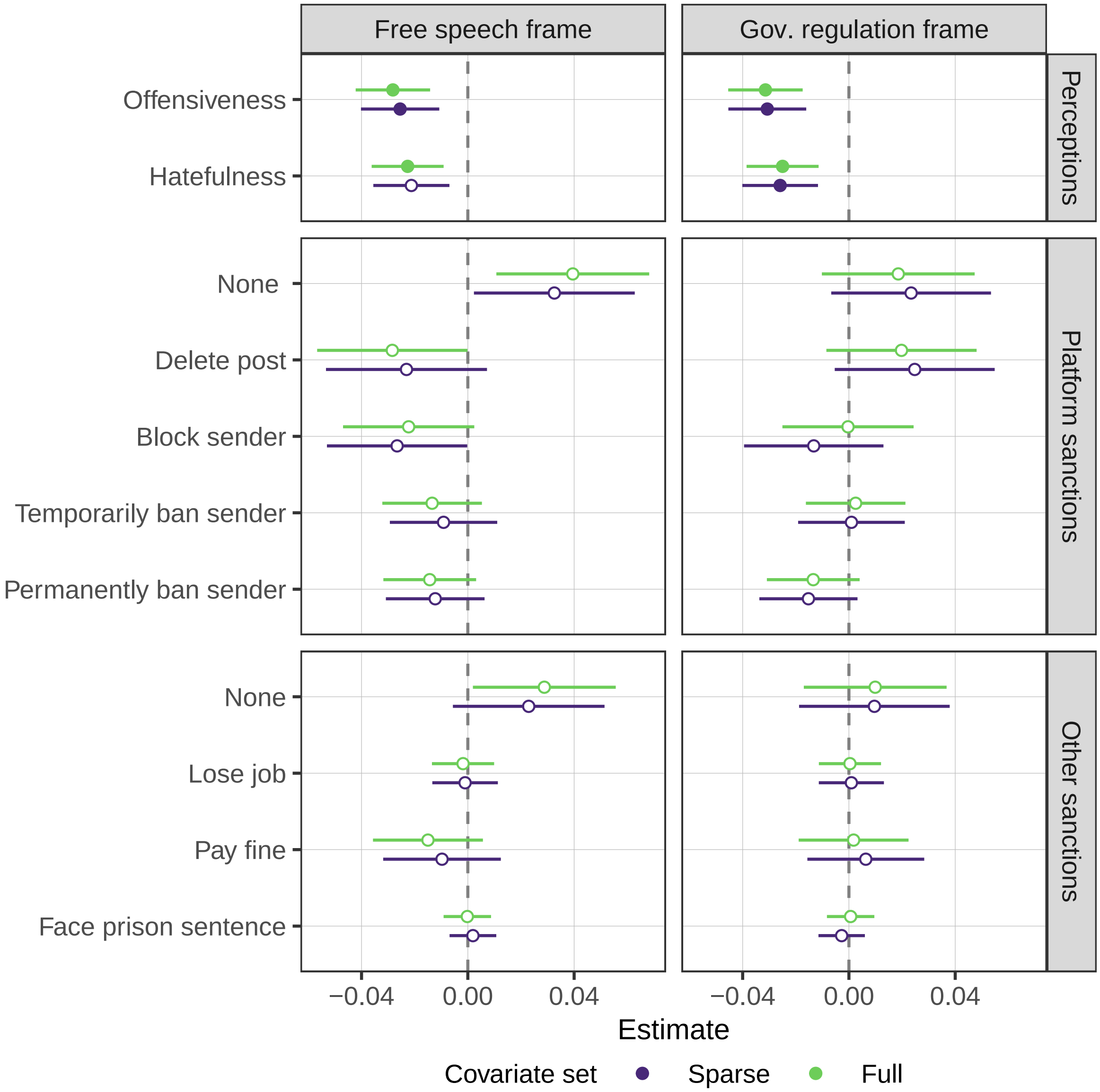

Figure S45: Estimated effects of free speech advocacy and government regulation frames (vs. neutral frame) on citizens' perceptions and preferred platform-side and other action (pooled sample; by covariate set; no subsetting based on attention check). The parse covariate setup only included the treatment indicator as fixed effects in the model; the full covariate setup included vignette attributes as well as respondent characteristics as fixed effects in the model. Perceived hatefulness and offensiveness are measured on 5-point scales (re-scaled to 0-1 scale), preferences for platform action and further penalties are binary measures. Error bars represent 95% confidence intervals.

Outcome	Gov. regulation frame	Free speech frame
Offensiveness	10	6
Hatefulness	3	1
None	−8	−3
Delete post	−8	−6
Block sender	−10	−7
Temporarily ban sender	−10	−8
Permanently ban sender	−8	−7
None	−9	−6
Lose job	−10	−10
Pay fine	−10	−8
Face prison sentence	−10	−10

Color legend: Evidence based on 2*log(Bayes Factor) (Raftery 1995)

weakly in favor of 0	positively in favor of 0	strongly in favor of 0	very strongly in favor of 0
weakly against 0	positively against 0	strongly against 0	very strongly against 0

Figure S46: Bayes factors ($2log$) against the Null for treatments in the framing experiment (Study 2, pooled sample). The table reports twice the natural logarithm of the Bayes factor and uses Kass and Raftery's scale for interpretation of evidence for/against the Null, with scale $\{]\text{-Inf}, -10],]-10, -6],]-6, -2],]-2, 0], [0, 2[, [2, 6[, [6, 10[, [10, \text{Inf}[\}$ ranging from "very strongly in favor of the Null" to "very strongly against the Null". Bayes factors are computed by assessing the pairwise ratio of marginal likelihoods for two models, one of which is specified with the predictor of interest and the other one without (which is the baseline model).

Outcome	Gov. regulation frame	Free speech frame
Offensiveness	1	−2
Hatefulness	−4	−7
None	−9	−9
Delete post	−8	−9
Block sender	−9	−7
Temporarily ban sender	−9	−9
Permanently ban sender	−9	−9
None	−9	−6
Lose job	−9	−9
Pay fine	−9	−8
Face prison sentence	−9	−9

Color legend: Evidence based on 2*log(Bayes Factor) (Raftery 1995)

weakly in favor of 0	positively in favor of 0	strongly in favor of 0	very strongly in favor of 0
weakly against 0	positively against 0	strongly against 0	very strongly against 0

Figure S47: Bayes factors ($2log$) against the Null for treatments in the framing experiment (Study 2, German sample). The table reports twice the natural logarithm of the Bayes factor and uses Kass and Raftery's scale for interpretation of evidence for/against the Null, with scale {]-Inf, -10], $]-10,-6]$, $]-6,-2]$, $]-2,0]$, $[0,2[$, $[2,6[$, $[6,10[$, [10, Inf[} ranging from "very strongly in favor of the Null" to "very strongly against the Null". Bayes factors are computed by assessing the pairwise ratio of marginal likelihoods for two models, one of which is specified with the predictor of interest and the other one without (which is the baseline model).

Outcome	Gov. regulation frame	Free speech frame
Offensiveness	0	−1
Hatefulness	−1	0
None	−8	3
Delete post	−9	0
Block sender	−9	−8
Temporarily ban sender	−9	−5
Permanently ban sender	−6	−7
None	−8	−8
Lose job	−9	−9
Pay fine	−9	−9
Face prison sentence	−9	−9

Color legend: Evidence based on 2*log(Bayes Factor) (Raftery 1995)

weakly in favor of 0	positively in favor of 0	strongly in favor of 0	very strongly in favor of 0
weakly against 0	positively against 0	strongly against 0	very strongly against 0

Figure S48: Bayes factors ($2log$) against the Null for treatments in the framing experiment (Study 2, U.S. sample). The table reports twice the natural logarithm of the Bayes factor and uses Kass and Raftery's scale for interpretation of evidence for/against the Null, with scale $\{]\text{-Inf}, -10],]-10, -6],]-6, -2],]-2, 0], [0, 2[, [2, 6[, [6, 10[, [10, \text{Inf}[\}$ ranging from "very strongly in favor of the Null" to "very strongly against the Null". Bayes factors are computed by assessing the pairwise ratio of marginal likelihoods for two models, one of which is specified with the predictor of interest and the other one without (which is the baseline model).

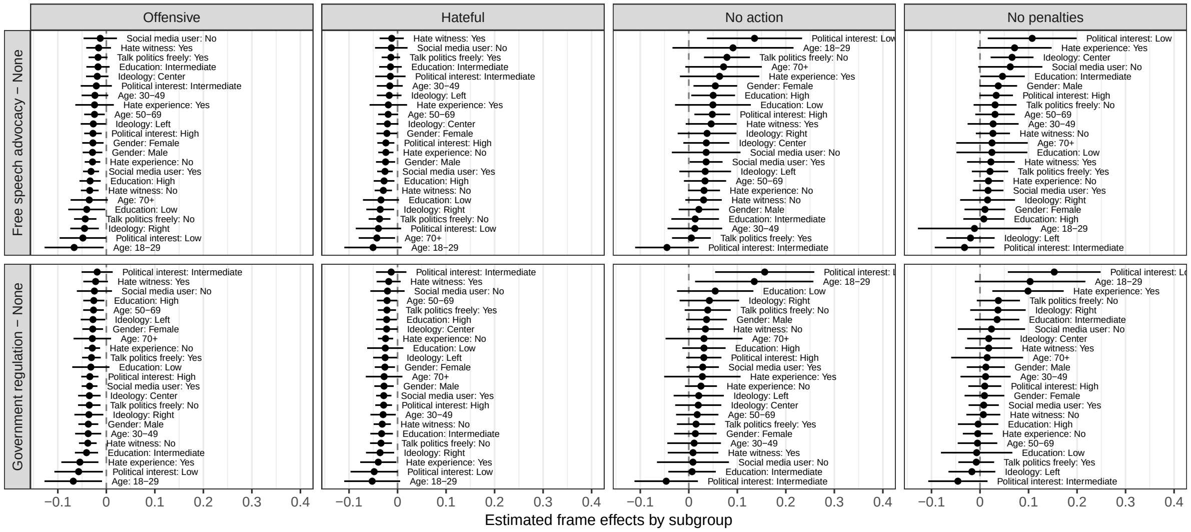

Figure S49: Estimated effects of free speech advocacy and government regulation frames (vs. neutral frame) on citizens' perceptions and preferred platform-side and other action (by subgroup). Perceived hatefulness and offensiveness are measured on 5-point scales (re-scaled to 0-1 scale), preferences for no platform action and no further penalties are binary measures. Error bars represent 95% confidence intervals.

D.5 Exposure experiment

Table S41: Descriptive statistics of respondent characteristics, by treatment group status (exposure experiment)

		Before (N=1223)		After (N=1197)	
		N	Pct.	N	Pct.
Gender	Male	630	51.5	605	50.5
	Female	593	48.5	592	49.5
Age	18-29	80	6.5	63	5.3
	30-49	350	28.6	347	29.0
	50-69	554	45.3	559	46.7
	70+	173	14.1	166	13.9
Education	Low	204	16.7	207	17.3
	Intermediate	484	39.6	460	38.4
	High	533	43.6	530	44.3
Political interest	Low	138	11.3	150	12.5
	Intermediate	261	21.3	236	19.7
	High	755	61.7	747	62.4
Social media user	No	208	17.0	199	16.6
	Yes	964	78.8	946	79.0
Ideology	Left	399	32.6	377	31.5
	Center	453	37.0	474	39.6
	Right	304	24.9	268	22.4
Hate experience	No	995	81.4	973	81.3
	Yes	162	13.2	162	13.5
Hate witness	No	760	62.1	771	64.4
	Yes	397	32.5	364	30.4
Talk politics freely	No	527	43.1	479	40.0
	Yes	651	53.2	651	54.4

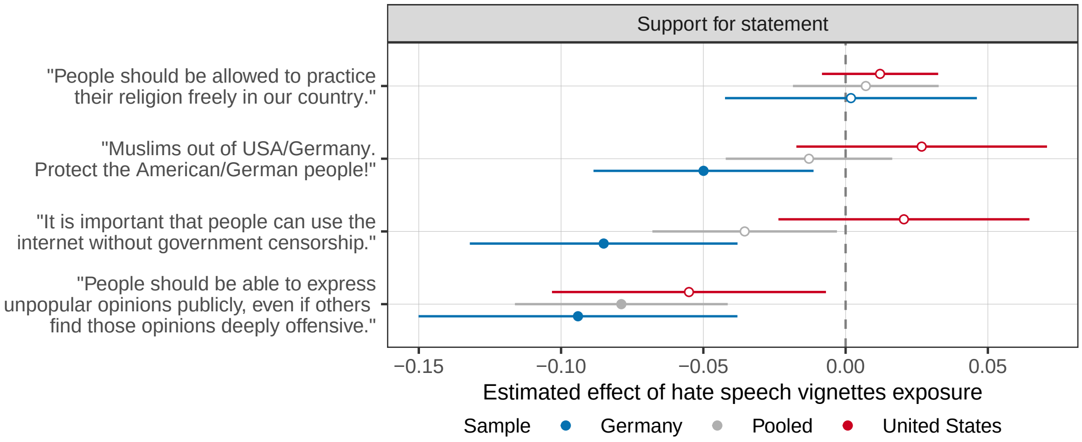

Figure S50: Estimated effects of speech vignette exposure on reported support for various statements, by country. Support is measured on binary scales. Error bars represent 95% confidence intervals. Filled circles indicate that the effect remains significant after *p*-value adjustment by controlling the false discovery rate. See Supplementary Tables S42 to S44 for detailed regression results.

	"Practice religion freely"		"Muslims out of country"		"Internet without censorship"		"Express unpopular opinions"	
	OLS	OLS w/covs	OLS	OLS w/covs	OLS	OLS w/covs	OLS	OLS w/covs
Intercept	0.84***	0.54***	0.25***	0.13*	0.83***	0.45***	0.74***	0.31***
	(0.01)	(0.06)	(0.01)	(0.06)	(0.01)	(0.07)	(0.01)	(0.08)
After vignettes	0.01	0.01	−0.04*	−0.01	−0.03*	−0.04*	−0.08***	−0.08***
	(0.01)	(0.01)	(0.02)	(0.01)	(0.02)	(0.02)	(0.02)	(0.02)
R^2	0.00	0.38	0.00	0.41	0.00	0.11	0.01	0.16
Adj. R^2	0.00	0.37	0.00	0.40	0.00	0.10	0.01	0.15
Num. obs.	2419	1929	2418	1927	2420	1929	2420	1929
RMSE	0.36	0.29	0.42	0.32	0.39	0.36	0.46	0.42

$^{***}p < 0.001$; $^{**}p < 0.01$; $^{*}p < 0.05$

Table S42: Effects of vignette positioning experiment on support for various direct statements (pooled sample). Support for direct items was measured on a binary scale, 0-1. OLS models without and with respondent covariates. Standard error in parentheses.

	"Practice religion freely"		"Muslims out of country"		"Internet without censorship"		"Express unpopular opinions"	
	OLS	OLS w/covs	OLS	OLS w/covs	OLS	OLS w/covs	OLS	OLS w/covs
Intercept	0.71***	0.64***	0.29***	0.20**	0.84***	0.47***	0.71***	0.59***
	(0.02)	(0.09)	(0.02)	(0.08)	(0.02)	(0.10)	(0.02)	(0.11)
After vignettes	0.01	0.00	−0.09***	−0.05*	−0.09***	−0.09***	−0.12***	−0.09**
	(0.03)	(0.02)	(0.02)	(0.02)	(0.02)	(0.02)	(0.03)	(0.03)
R^2	0.00	0.38	0.01	0.48	0.01	0.12	0.01	0.11
Adj. R^2	−0.00	0.37	0.01	0.47	0.01	0.10	0.01	0.10
Num. obs.	1188	1000	1188	1000	1188	1000	1188	1000
RMSE	0.45	0.35	0.43	0.31	0.40	0.37	0.47	0.45

$^{***}p < 0.001$; $^{**}p < 0.01$; $^{*}p < 0.05$

Table S43: Effects of vignette positioning experiment on support for various direct statements (German sample). Support for direct items was measured on a binary scale, 0-1. OLS models without and with respondent covariates. Standard error in parentheses.

	"Practice religion freely"		"Muslims out of country"		"Internet without censorship"		"Express unpopular opinions"	
	OLS	OLS w/covs	OLS	OLS w/covs	OLS	OLS w/covs	OLS	OLS w/covs
Intercept	0.95***	0.81***	0.22***	0.05	0.83***	0.48***	0.77***	0.15
	(0.01)	(0.08)	(0.02)	(0.12)	(0.01)	(0.13)	(0.02)	(0.12)
After vignettes	0.02	0.01	0.01	0.03	0.02	0.02	−0.05*	−0.06*
	(0.01)	(0.01)	(0.02)	(0.02)	(0.02)	(0.02)	(0.02)	(0.02)
R^2	0.00	0.08	0.00	0.37	0.00	0.12	0.00	0.25
Adj. R^2	0.00	0.06	−0.00	0.35	−0.00	0.10	0.00	0.24
Num. obs.	1231	929	1230	927	1232	929	1232	929
RMSE	0.19	0.16	0.42	0.34	0.37	0.35	0.44	0.37

$^{***}p < 0.001$; $^{**}p < 0.01$; $^{*}p < 0.05$

Table S44: Effects of vignette positioning experiment on support for various direct statements (U.S. sample). Support for direct items was measured on a binary scale, 0-1. OLS models without and with respondent covariates. Standard error in parentheses.

Outcome	After vignettes exposure
"Practice religion freely"	−7
"Muslims out of country"	−3
"Internet without censorship"	−3
"Express unpopular opinions"	12

Color legend: Evidence based on 2*log(Bayes Factor) (Raftery 1995)

weakly in favor of 0	positively in favor of 0	strongly in favor of 0	very strongly in favor of 0
weakly against 0	positively against 0	strongly against 0	very strongly against 0

Figure S51: Bayes factors ($2log$) against the Null for treatment exposure experiment (Study 3, pooled sample). The table reports twice the natural logarithm of the Bayes factor and uses Kass and Raftery's scale for interpretation of evidence for/against the Null, with scale $\{]\text{-Inf}, -10],]-10, -6],]-6, -2],]-2, 0], [0, 2[, [2, 6[, [6, 10[, [10, \text{Inf}[\}$ ranging from "very strongly in favor of the Null" to "very strongly against the Null". Bayes factors are computed by assessing the pairwise ratio of marginal likelihoods for two models, one of which is specified with the predictor of interest and the other one without (which is the baseline model).

Outcome	After vignettes exposure
"Practice religion freely"	−7
"Muslims out of country"	6
"Internet without censorship"	6
"Express unpopular opinions"	11

Color legend: Evidence based on 2*log(Bayes Factor) (Raftery 1995)

weakly in favor of 0	positively in favor of 0	strongly in favor of 0	very strongly in favor of 0
weakly against 0	positively against 0	strongly against 0	very strongly against 0

Figure S52: Bayes factors ($2log$) against the Null for treatment exposure experiment (Study 3, German sample). The table reports twice the natural logarithm of the Bayes factor and uses Kass and Raftery's scale for interpretation of evidence for/against the Null, with scale $\{]\text{-Inf}, -10],]-10, -6],]-6, -2],]-2, 0], [0, 2[, [2, 6[, [6, 10[, [10, \text{Inf}[\}$ ranging from "very strongly in favor of the Null" to "very strongly against the Null". Bayes factors are computed by assessing the pairwise ratio of marginal likelihoods for two models, one of which is specified with the predictor of interest and the other one without (which is the baseline model).

Outcome	After vignettes exposure
"Practice religion freely"	−4
"Muslims out of country"	−7
"Internet without censorship"	−6
"Express unpopular opinions"	−3

Color legend: Evidence based on 2*log(Bayes Factor) (Raftery 1995)

weakly in favor of 0	positively in favor of 0	strongly in favor of 0	very strongly in favor of 0
weakly against 0	positively against 0	strongly against 0	very strongly against 0

Figure S53: Bayes factors ($2log$) against the Null for treatment exposure experiment (Study 3, U.S. sample). The table reports twice the natural logarithm of the Bayes factor and uses Kass and Raftery's scale for interpretation of evidence for/against the Null, with scale $\{]\text{-Inf}, -10],]-10, -6],]-6, -2],]-2, 0], [0, 2[, [2, 6[, [6, 10[, [10, \text{Inf}[\}$ ranging from "very strongly in favor of the Null" to "very strongly against the Null". Bayes factors are computed by assessing the pairwise ratio of marginal likelihoods for two models, one of which is specified with the predictor of interest and the other one without (which is the baseline model).

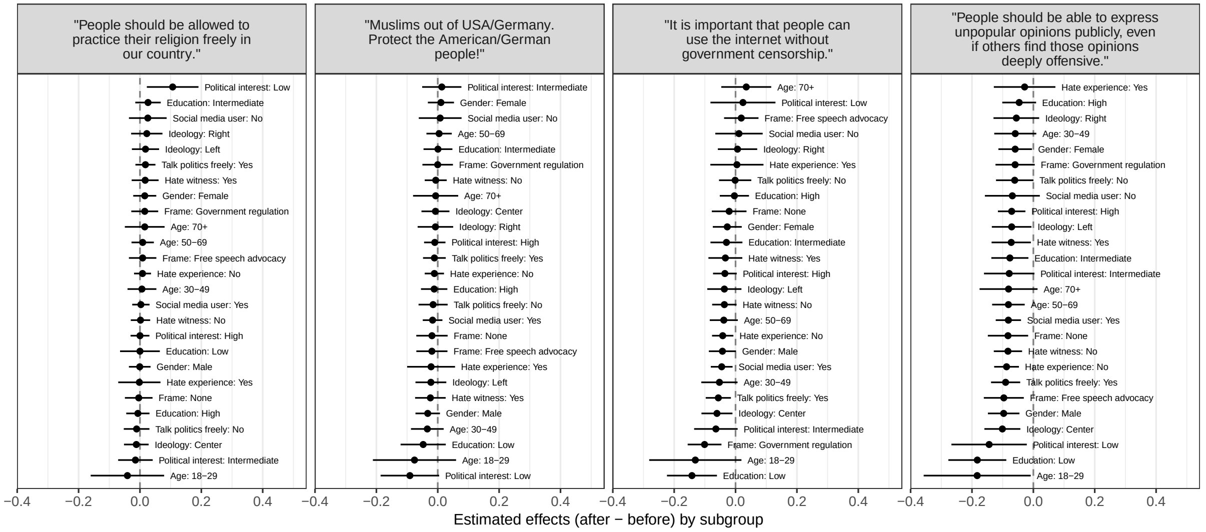

Figure S54: Estimated effects of hate speech vignettes exposure on reported support of various statements (by subgroup). Support is measured on binary scales. Error bars represent 95% confidence intervals.

E Survey questionnaire

This section shows the specific wordings we used for all the survey items used as part of this study. In square brackets we indicate the wave in which the questions were asked.

E.1 Sociodemographic variables

GENDER [USA W1, GER W1]

USA: Please indicate your gender

GER: Bitte geben Sie Ihr Geschlecht an.

1. Male *(GER: Männlich)*
2. Female *(GER: Weiblich)*

YEAR OF BIRTH [USA W1, GER W1]

USA: In which year were you born?

GER: In welchem Jahr sind Sie geboren?

[Open text box]

HIGHEST LEVEL OF EDUCATION [USA W1, GER W1]

USA: What is your highest level of education?

GER: Welchen höchsten Schulabschluss haben Sie?

1. No HS
2. High school graduate
3. Some college
4. 2-year
5. 4-year
6. Post-grad

GER:

1. Still in school education
2. Secondary (elementary) school leaving certificate
3. Realschule or equivalent qualification (POS, Mittlere Reife)
4. High school diploma, entrance qualification for a university of applied sciences
5. No school-leaving qualification

E.2 Behaviors and attitudes related to hate speech

HATE SPEECH EXPERIENCE [USA W4, GER W6]

USA: "Hate Speech" describes when someone is verbally attacked because of personal attributes, such as religion, ethnic origin, nationality, sex, or opinions. Please select all of the following that apply to you.

GER: Als "Hate Speech" wird bezeichnet, wenn jemand wegen persönlicher Eigenschaften wie Religion, ethnischen Zugehörigkeit, Nationalität, Geschlecht oder Meinung verbal angegriffen wird. Bitte wählen Sie diejenigen der folgenden Aussagen aus, die auf Sie zutreffen.

(A) I have personally been verbally attacked with hate speech online. *(GER: Ich bin bereits persönlich online mit Hate Speech angegriffen worden.)*

(B) I have experienced how others have been verbally attacked with hate speech online. *(GER:] Ich habe selbst schon erlebt, wie andere online mit Hate Speech angegriffen worden sind.)*

(C) None of the above. *(GER: Keines der genannten.)*

GROUP-SPECIFIC HATE SPEECH REGULATION [USA W4, GER W6]

USA: Would you support or oppose a law that would make it illegal to make insulting or hateful statements about...

GER: Sollte es Ihrer Meinung nach verboten oder erlaubt werden, in der Öffentlichkeit beleidigende oder hasserfüllte Äußerungen über die folgenden Gruppen zu tätigen?

(A) Americans? *(GER: Deutsche?)*

(B) Muslims? *(GER: Muslime?)*

(C) Jews? *(GER: Juden?)*

(D) Women? *(GER: Frauen?)*

(E) Christians? *(GER: Christen?)*

(F) Immigrants? *(GER: Einwandere?)*

(G) Neo Nazis? *(GER: Neonazis?)*

(H) The Government? *(GER: Die Regierung?)*

1. Definitely make it illegal *(GER: Würde Gesetz total zustimmen)*
2. Possibly make it illegal *(GER: Würde Gesetz eher zustimmen)*
3. Possibly allow *(GER: Würde Gesetz eher ablehnen)*
4. Definitely allow *(GER: Würde Gesetz total)*

– Don't know

HATE SPEECH DECLARATION [USA W4, GER W6]

USA: Which of the following would you label as hate speech?

GER: Welche der folgenden Fälle würden Sie als "Hate Speech" bezeichnen? Eine Person, die...

(A) A person calling an ethnic minority a racial slur. *(GER: eine ethnische Minderheit rassistisch beleidigt.)*

(B) A person calling a woman a vulgar name. *(GER: eine Frau vulgär beleidigt.)*

(C) A person who says that illegal immigrants should be deported. *(GER: sagt, dass illegale Migranten abgeschoben werden sollten.)*

(D) A person who says Germany is an evil country. *(GER: Deutschland als bösartiges Land bezeichnet.)*

(E) A person who says that all white people are racist. *(GER: sagt, dass alle weißen Menschen Rassisten seien.)*

(F) A person who says Islam is taking over Europe. *(GER: sagt, dass der Islam Europa erobere.)*

(G) A person calling another person with conservative views a Nazi. *(GER: eine andere Person mit konservativen Ansichten als Nazi bezeichnet.)*

1. Hate speech *(GER: Hate speech)*
2. No hate speech *(GER: Kein hate speech)*

– Don't know

HATE SPEECH RESPONSIBILITY [USA W4, GER W6]

USA: To what extent, if at all, do you think each of the following groups should take responsibility in taking steps against online hate speech?

GER: Inwiefern sollten die folgenden Akteure Ihrer Meinung nach verantwortlich dafür sein, gegen Hate Speech im Internet vorzugehen?

(A) People who are victims of online hate speech *(GER: Menschen, die im Internet Opfer von Hate Speech werden)*

(B) Other users who witness the behavior *(GER: Andere Menschen, die Zeuge von Hate Speech im Internet werden)*

(C) Online services such as social media platforms or other websites *(GER: Onlineplattformen wie Social-Media-Seiten oder andere Webseiten)*

(D) Policymakers *(GER: Politiker)*

(E) Law enforcement *(GER: Justiz und Polizei)*

(F) Employers of distributors of hate speech *(GER: Arbeitgeber von Hate-Speech-Verbreitern)*

1. No responsibility at all *(GER: Überhaupt nicht verantwortlich)*
2. Almost no responsibility *(GER: Eher nicht verantwortlich)*
3. Some responsibility *(GER: Eher verantwortlich)*
4. Very much responsibility *(GER: In hohem Maße verantwortlich)*

– Don't know

E.3 Instruments for vignette evaluations

PERCEIVED OFFENSIVENESS [USA W5/6, GER W7/9]

Looking at the post marked with a red arrow, what do you think, how offensive is this post?

GER: Für wie beleidigend halten Sie die Nachricht, die mit dem roten Pfeil markiert ist?

1. Extremely offensive *(GER: Sehr beleidigend)*
2. Very offensive *(GER: Ziemlich beleidigend)*
3. Somewhat offensive *(GER: Eher beleidigend)*
4. Not very offensive *(GER: Nicht sehr beleidigend)*
5. Not offensive at all *(GER: Überhaupt nicht beleidigend)*

PERCEIVED HATEFULNESS [USA W5/6, GER W7/9]

And what do you think, how hateful is this post?

GER: Für wie hasserfüllt halten Sie diese Nachricht?

1. Extremely hateful *(GER: Sehr hasserfüllt)*
2. Very hateful *(GER: Ziemlich hasserfüllt)*
3. Somewhat hateful *(GER: Eher hasserfüllt)*
4. Not very hateful *(GER: Nicht sehr hasserfüllt)*
5. Not hateful at all *(GER: Überhaupt nicht hasserfüllt)*

ACTIONS BY PLATFORM PROVIDER [USA W5/6, GER W7/9]

What actions should be taken by the platform providers? Select all that you find appropriate in this case.

GER: Welche Maßnahmen sollt der Plattformanbieter treffen? Wählen Sie alles aus, was Sie in diesem Fall für angemessen halten.

1. No action should be taken. *(GER: Der Plattformanbieter sollte keine Maßnahmen treffen.)*
2. The post should be deleted. *(GER: Die Nachricht sollte gelöscht werden.)*
3. The sender of the message should be blocked from posting to the target of this message. *(GER: Dem Sender der Nachricht sollte es nicht möglich sein, weitere Nachrichten an das Ziel dieser Nachricht zu senden.)*
4. The sender of the message should be temporarily banned from the platform. *(GER: Der Sender der Nachricht sollte zeitweise von der Plattform ausgesperrt werden.)*
5. The sender of the message should be permanently banned from the platform. *(GER: Der Sender der Nachricht sollte dauerhaft von der Plattform ausgesperrt werden.)*

OTHER ACTIONS [USA W5/6, GER W7/9]

What other actions should be taken? Select all that you find appropriate in this case.

GER: Welche anderen Maßnahmen sollten getroffen werden? Wählen Sie alles aus, was Sie in diesem Fall für angemessen halten.

1. No further action should be taken. *(GER: Es sollten keine weiteren Maßnahmen getroffen werden.)*
2. The sender of the message should lose his/her job. *(GER: Der Sender der Nachricht sollte seine Arbeit verlieren.)*
3. A fine should be forced on the sender of the message. *(GER: Der Sender der Nachricht sollte eine Geldstrafe zahlen.)*
4. A prison sentence should be forced on the sender of the message. *(GER: Der Sender der Nachricht sollte eine Haftstrafe erhalten.)*

E.4 Instruments for framing experiment

ATTENTION CHECK BEFORE FRAMING EXPERIMENT [USA W5/6, GER W7]

USA: Many people own smart phones nowadays. How about you: Do you own one, and if yes, what type of smartphone? Specifically, we want to know whether you actually take your time to read the questions and follow our instructions. To demonstrate that you read this far, skip this question and just type ”read” in the text field below.

GER: Viele Leute besitzen heutzutage ein Smartphone. Wie ist das mit Ihnen? Besitzen Sie ein Smartphone, und wenn ja, welches? Genauer gesagt möchten wir von Ihnen wissen, ob Sie sich eigentlich die Zeit nehmen, die Fragen zu lesen und den Anweisungen zu folgen. Um zu zeigen, dass Sie bis hierhin gelesen haben, tragen Sie bitte ”gelesen” in das Feld ”Anderes, und zwar” unten ein.

1. Apple iPhone
2. Samsung Galaxy
3. Huawei Mate
4. Google Pixel
5. LG V40
6. Sony Xperia
7. Other (please specify): ______________________
8. I do not own a smart phone.

VIGNETTE TASK INTRODUCTION, NEUTRAL FRAME [USA W7, GER W7/9]

In the following, you will see a couple of messages posted online by social media users. Some of these messages, marked with a red arrow, are potentially problematic. We want you to take a close look at these messages and then answer a few questions. We also want to point out that the contents of some of these messages may be unpleasant or repugnant to you. If you do not want to see any more such messages, you can skip these questions without answering.
(GER: Im Folgenden sehen Sie Nachrichten, die von Social-Media-Nutzern gepostet wurden. Einige dieser Nachrichten, markiert mit einem roten Pfeil, sind potentiell problematisch. Wir möchten, dass Sie sich diese Nachrichten genau ansehen und anschließend einige Fragen dazu beantworten. Wir möchten Sie außerdem darauf hinweisen, dass die Inhalte einiger dieser Nachrichten möglicherweise unangenehm oder abstoßend auf Sie wirken könnten. Wenn Sie deshalb keine weiteren solchen Nachrichten sehen möchten, können Sie diese Fragen ohne zu antworten überspringen.)

VIGNETTE TASK INTRODUCTION, GOVERNMENT REGULATION FRAME [USA W7, GER W7/9]

As you may have heard, the government is serious about tackling online hate speech. Potential victims of online hate speech should be protected. This means that a large number of social media messages containing offensive or hateful content will be deleted and prosecuted. In the following, you will see a couple of messages posted online by social media users. Some of these messages, marked with a red arrow, are potentially problematic. We want you to take a close look at these messages and then answer a few questions. We also want to point out that the contents of some of these messages may be unpleasant or repugnant to you. If you do not want to see any more such messages, you can skip these questions without answering.
(GER: Wie Sie vielleicht gehört haben, bemüht sich die Regierung sehr ernsthaft Online-Hassrede zu bekämpfen. Potentielle Opfer von Online-Hassrede sollen so geschützt werden. Das bedeutet, dass eine große Anzahl an Social-Media-Nachrichten mit beleidigenden oder hasserfüllten Inhalten gelöscht und strafrechtlich verfolgt werden. Im Folgenden sehen Sie Nachrichten, die von Social-Media-Nutzern gepostet wurden. Einige dieser Nachrichten, markiert mit einem roten Pfeil, sind potentiell problematisch. Wir möchten, dass Sie sich diese Nachrichten genau ansehen und anschließend einige Fragen dazu beantworten. Wirr möchten Sie außerdem darauf hinweisen, dass die Inhalte einiger dieser Nachrichten möglicherweise unangenehm oder abstoßend auf Sie wirken könnten. Wenn Sie deshalb keine weiteren solchen Nachrichten sehen möchten, können Sie diese Fragen ohne zu antworten überspringen.)

VIGNETTE TASK INTRODUCTION, FREE SPEECH ADVOCACY FRAME [USA W7, GER W7/9]

As you may have heard, civil society organizations are struggling to counter censorship of content on the Net. The right to freedom of expression should be protected. This means that social media messages containing offensive or hateful content should not be deleted or prosecuted. In the following, you will see a couple of messages posted online by social media users. Some of these messages, marked with a red arrow, are potentially problematic. We want you to take a close look at these messages and then answer a few questions. We also want to point out that the contents of some of these messages may be unpleasant or repugnant to you. If you do not want to see any more such messages, you can skip these questions without answering.
(GER: Wie Sie vielleicht gehört haben, bemühen sich Bürgerrechtsorganisationen darum, der Zensur von Inhalten im Netz entgegenzutreten. Das Recht auf freie Meinungsäußerung soll so geschützt werden. Das bedeutet, dass Social-Media-Nachrichten mit beleidigenden oder hasserfüllten Inhalten nicht gelöscht oder strafrechtlich verfolgt werden sollten. Im Folgenden sehen Sie Nachrichten, die von Social-Media-Nutzern gepostet wurden. Einige dieser Nachrichten, markiert mit einem roten Pfeil, sind potentiell problematisch. Wir möchten, dass Sie sich diese Nachrichten genau ansehen und anschließend einige Fragen dazu beantworten. Wir möchten Sie außerdem darauf hinweisen, dass die Inhalte einiger dieser Nachrichten möglicherweise unangenehm oder abstoßend auf Sie wirken könnten. Wenn Sie deshalb keine weiteren solchen Nachrichten sehen möchten, können Sie diese Fragen ohne zu antworten überspringen.)

E.5 Instruments for exposure experiment

SELECTED OPINIONS [USA W5/6, GER W7/9]

Note: This item was randomly placed. Half of the respondents received the item before the attention check, the other half after finishing the vignettes. USA: Here you can find several statements made on social media that some people support while others oppose. Do you support or oppose these statements?

GER: Hier sind einige Aussagen, die in den sozialen Medien gemacht wurden und die manche Leute unterstützen, andere ablehnen. Unterstützen Sie diese Aussagen oder lehnen Sie sie ab?

(A) People should be allowed to practice their religion freely in our country. *(GER: Die Leute sollten ihre Religion in unserem Land frei ausüben dürfen.)*

(B) Muslims out of USA. Protect the American people! *(GER: Muslime raus aus Deutschland. Schützt das Deutsche Volk!)*

(C) It is important that people can use the Internet without government censorship. *(GER: Es ist wichtig, dass die Leute das Internet ohne Zensur durch die Regierung nutzen können.)*

(D) People should be allowed to express unpopular opinions publicly, even if others find those opinions deeply offensive. *(GER: Die Leute sollten unbeliebte Meinungen öffentlich äußern dürfen, selbst wenn andere diese Meinungen zutiefst anstößig finden.)*

1. Support *(GER: Unterstütze)*
2. Oppose *(GER: Lehne ab)*

E.6 Other covariates

INTEREST IN POLITICS [USA W1, GER W1]

USA: In general, how interested are you in politics?

GER: Wie stark interessieren Sie sich im Allgemeinen für Politik?

1. Not interested at all (*GER: überhaupt nicht*)
2. Slightly interested (*GER: weniger stark*)
3. Moderately interested (*GER: mittelmäßig*)
4. Interested (*GER: stark*)
5. Very interested (*GER: sehr stark*)
6. Don't know (*GER: weiß nicht*)

POLITICAL IDEOLOGY, 5-PTS SCALE [USA W1]

USA: In general, how would you describe your own political viewpoint?

1. Very liberal
2. Liberal
3. Moderate
4. Conservative
5. Very conservative
6. Not sure

POLITICAL IDEOLOGY, 11-PTS SCALE [GER W1]

USA: In politics people often talk about "left" and "right". On a scale between 1 and 11, where would you rate yourself?

GER: In der Politik reden die Leute häufig von "links" und "rechts". Wo würden Sie sich auf einer Skala von 1 bis 11 einordnen?

[Scale 1-11]

PARTY IDENTIFICATION [USA W1]

USA: Generally speaking, do you think of yourself as a ...?

1. Democrat
2. Republican
3. Independent
4. Other
5. Not sure

PARTY IDENTIFICATION [GER W1]

GER: Bei der Bundestagswahl können Sie ja zwei Stimmen vergeben. Die Erststimme für einen Kandidaten aus Ihrem Wahlkreis und die Zweitstimme für eine Partei. Was werden Sie auf Ihrem Stimmzettel ankreuzen?

(A) Erststimme

(B) Zweitstimme

1. CDU/CSU
2. SPD
3. FDP
4. Bündnis 90/Die Grünen
5. Die Linke
6. AfD
7. Andere Partei, und zwar: ______________________
8. Weiß ich noch nicht

FEELING ABOUT DISCUSSING POLITICS [USA W5, GER W7]

When you discuss politics with others, how free or unrestricted do you feel?

GER: Wenn sie über mit anderen über Politik diskutieren, wie frei fühlen Sie sich dabei?

1. I don't feel free to discuss politics with anyone (*GER: Ich fühle mich bei niemandem frei über Politik zu diskutieren*)
2. I feel free to discuss politics with only a few people (*GER: Ich fühle mich bei den meisten Leuten nicht frei über Politik zu diskutieren*)
3. I feel free to discuss politics with most people (*GER: Ich fühle mich bei den meisten Leuten frei über Politik zu diskutieren*)
4. I feel free to discuss politics with anyone (*GER: Ich fühle mich bei allen frei über Politik zu diskutieren*)
5. I never discuss politics with other people (*GER: Ich diskutiere nie mit anderen über Politik*)

SOCIAL MEDIA ACCOUNTS [USA W1, GER W1]

Do you have accounts on any of the following social media sites? (check all that apply)

GER: Haben Sie einen Account bei den folgenden Social-Media-Seiten? (bitte kreuzen Sie alle an, auf die dies zutrifft)

1. Twitter
2. Facebook
3. Instagram
4. LinkedIn
5. Snapchat
6. Whatsapp
7. Reddit
8. None of the above

References

1. J. Schuessler, M. Freitag, Power analysis for conjoint experiments. (2020).
